# Supplementary material for: Spinal mechanisms and feasibility of Dry Needling versus Botulinum Toxin Type A in post-stroke lower limb spasticity: A proof-of-concept randomized clinical trial protocol (STROKE-POC)
Source: PLoS One. 2026 May 20;21(5):e0334571. doi: 10.1371/journal.pone.0334571 (PMC13189288; doi:10.1371/journal.pone.0334571)
Supplement: S1 File — (PDF) [file pone.0334571.s001.pdf]

SPIRIT 2013 Checklist: Recommended items to address in a clinical trial protocol and related documents*

| Section/item | Item No | Description | Addressed on page number |
|---|---|---|---|
| **Administrative information** | | | |
| Title | 1 | Descriptive title identifying the study design, population, interventions, and, if applicable, trial acronym | Pag 3 |
| Trial registration | 2a | Trial identifier and registry name. If not yet registered, name of intended registry | Pag 3 |
| | 2b | All items from the World Health Organization Trial Registration Data Set | Attached to SPIRIT checklist doc. |
| Protocol version | 3 | Date and version identifier | Pag. 3 |
| Funding | 4 | Sources and types of financial, material, and other support | Pag.8 |
| Roles and responsibilities | 5a | Names, affiliations, and roles of protocol contributors | Pag 1-2 |
| | 5b | Name and contact information for the trial sponsor | Pag 1 of the World Health Organization Trial Registration Data Set attached to the SPIRIT checklist doc. |
| | 5c | Role of study sponsor and funders, if any, in study design; collection, management, analysis, and interpretation of data; writing of the report; and the decision to submit the report for publication, including whether they will have ultimate authority over any of these activities | Pag 8 |
| | 5d | Composition, roles, and responsibilities of the coordinating centre, steering committee, endpoint adjudication committee, data management team, and other individuals or groups overseeing the trial, if applicable (see Item 21a for data monitoring committee) | Pag 6-7 |

**Introduction**

| | | | |
|---|---|---|---|
| Background and rationale | 6a | Description of research question and justification for undertaking the trial, including summary of relevant studies (published and unpublished) examining benefits and harms for each intervention | Pag 5 and 8 |
| | 6b | Explanation for choice of comparators | Pag 5-6 |
| Objectives | 7 | Specific objectives or hypotheses | Pag 3-4 |
| Trial design | 8 | Description of trial design including type of trial (eg, parallel group, crossover, factorial, single group), allocation ratio, and framework (eg, superiority, equivalence, noninferiority, exploratory) | Pag 4 |

**Methods: Participants, interventions, and outcomes**

| | | | |
|---|---|---|---|
| Study setting | 9 | Description of study settings (eg, community clinic, academic hospital) and list of countries where data will be collected. Reference to where list of study sites can be obtained | Pag 5 |
| Eligibility criteria | 10 | Inclusion and exclusion criteria for participants. If applicable, eligibility criteria for study centres and individuals who will perform the interventions (eg, surgeons, psychotherapists) | Pag 4-5 |
| Interventions | 11a | Interventions for each group with sufficient detail to allow replication, including how and when they will be administered | Pag 5-6 |
| | 11b | Criteria for discontinuing or modifying allocated interventions for a given trial participant (eg, drug dose change in response to harms, participant request, or improving/worsening disease) | Main document pag 5; Ethics document |
| | 11c | Strategies to improve adherence to intervention protocols, and any procedures for monitoring adherence (eg, drug tablet return, laboratory tests) | Main doc. pag 6 |
| | 11d | Relevant concomitant care and interventions that are permitted or prohibited during the trial | Main doc. pag 5-6; Ethics document |
| Outcomes | 12 | Primary, secondary, and other outcomes, including the specific measurement variable (eg, systolic blood pressure), analysis metric (eg, change from baseline, final value, time to event), method of aggregation (eg, median, proportion), and time point for each outcome. Explanation of the clinical relevance of chosen efficacy and harm outcomes is strongly recommended | Main doc. pag 6 |
| Participant timeline | 13 | Time schedule of enrolment, interventions (including any run-ins and washouts), assessments, and visits for participants. A schematic diagram is highly recommended (see Figure) | Main doc. pag 6 |

| | | | |
|---|---|---|---|
| Sample size | 14 | Estimated number of participants needed to achieve study objectives and how it was determined, including clinical and statistical assumptions supporting any sample size calculations | Main doc. pag 8 |
| Recruitment | 15 | Strategies for achieving adequate participant enrolment to reach target sample size | Main doc. pag 6 |

**Methods: Assignment of interventions (for controlled trials)**

| | | | |
|---|---|---|---|
| Allocation: | | | |
| Sequence generation | 16a | Method of generating the allocation sequence (eg, computer-generated random numbers), and list of any factors for stratification. To reduce predictability of a random sequence, details of any planned restriction (eg, blocking) should be provided in a separate document that is unavailable to those who enrol participants or assign interventions | Main doc. pag 5 |
| Allocation concealment mechanism | 16b | Mechanism of implementing the allocation sequence (eg, central telephone; sequentially numbered, opaque, sealed envelopes), describing any steps to conceal the sequence until interventions are assigned | Main doc. pag 5 |
| Implementation | 16c | Who will generate the allocation sequence, who will enrol participants, and who will assign participants to interventions | Main doc. pag 5 |
| Blinding (masking) | 17a | Who will be blinded after assignment to interventions (eg, trial participants, care providers, outcome assessors, data analysts), and how | Main doc. pag 5 |
| | 17b | If blinded, circumstances under which unblinding is permissible, and procedure for revealing a participant's allocated intervention during the trial | Main doc. pag 5 |

**Methods: Data collection, management, and analysis**

| | | | |
|---|---|---|---|
| Data collection methods | 18a | Plans for assessment and collection of outcome, baseline, and other trial data, including any related processes to promote data quality (eg, duplicate measurements, training of assessors) and a description of study instruments (eg, questionnaires, laboratory tests) along with their reliability and validity, if known. Reference to where data collection forms can be found, if not in the protocol | Main doc. pag 6 and 9 |
| | 18b | Plans to promote participant retention and complete follow-up, including list of any outcome data to be collected for participants who discontinue or deviate from intervention protocols | Main doc. pag 6 and 9 |

| | | | |
|---|---|---|---|
| Data management | 19 | Plans for data entry, coding, security, and storage, including any related processes to promote data quality (eg, double data entry; range checks for data values). Reference to where details of data management procedures can be found, if not in the protocol | Main doc. pag 5 and 9 |
| Statistical methods | 20a | Statistical methods for analysing primary and secondary outcomes. Reference to where other details of the statistical analysis plan can be found, if not in the protocol | Main doc. pag 7 |
| | 20b | Methods for any additional analyses (eg, subgroup and adjusted analyses) | Main doc. pag 6 and 7 |
| | 20c | Definition of analysis population relating to protocol non-adherence (eg, as randomised analysis), and any statistical methods to handle missing data (eg, multiple imputation) | Main doc. pag 7 |
| **Methods: Monitoring** | | | |
| Data monitoring | 21a | Composition of data monitoring committee (DMC); summary of its role and reporting structure; statement of whether it is independent from the sponsor and competing interests; and reference to where further details about its charter can be found, if not in the protocol. Alternatively, an explanation of why a DMC is not needed | Main doc. pag 6 |
| | 21b | Description of any interim analyses and stopping guidelines, including who will have access to these interim results and make the final decision to terminate the trial | Main doc. pag 9 |
| Harms | 22 | Plans for collecting, assessing, reporting, and managing solicited and spontaneously reported adverse events and other unintended effects of trial interventions or trial conduct | Main doc. pag 7 |
| Auditing | 23 | Frequency and procedures for auditing trial conduct, if any, and whether the process will be independent from investigators and the sponsor | Main do. pag 6 |
| **Ethics and dissemination** | | | |
| Research ethics approval | 24 | Plans for seeking research ethics committee/institutional review board (REC/IRB) approval | Section 21 of the World Health Organization Trial Registration Data Set attached to SPIRIT checklist |
| Protocol amendments | 25 | Plans for communicating important protocol modifications (eg, changes to eligibility criteria, outcomes, analyses) to relevant parties (eg, investigators, REC/IRBs, trial participants, trial registries, journals, regulators) | Main doc. pag 7 |

| | | | |
|---|---|---|---|
| Consent or assent | 26a | Who will obtain informed consent or assent from potential trial participants or authorised surrogates, and how (see Item 32) | Main doc. pag 5 |
| | 26b | Additional consent provisions for collection and use of participant data and biological specimens in ancillary studies, if applicable | Not applicable |
| Confidentiality | 27 | How personal information about potential and enrolled participants will be collected, shared, and maintained in order to protect confidentiality before, during, and after the trial | Main doc. pag 9 |
| Declaration of interests | 28 | Financial and other competing interests for principal investigators for the overall trial and each study site | Main doc. pag 7 |
| Access to data | 29 | Statement of who will have access to the final trial dataset, and disclosure of contractual agreements that limit such access for investigators | Ethics documents |
| Ancillary and post-trial care | 30 | Provisions, if any, for ancillary and post-trial care, and for compensation to those who suffer harm from trial participation | Not applicable |
| Dissemination policy | 31a | Plans for investigators and sponsor to communicate trial results to participants, healthcare professionals, the public, and other relevant groups (eg, via publication, reporting in results databases, or other data sharing arrangements), including any publication restrictions | Ethics documents |
| | 31b | Authorship eligibility guidelines and any intended use of professional writers | Main doc. pag 1 to 3 |
| | 31c | Plans, if any, for granting public access to the full protocol, participant-level dataset, and statistical code | Ethics documents |
| **Appendices** | | | |
| Informed consent materials | 32 | Model consent form and other related documentation given to participants and authorised surrogates | Informed consent doument (ethics) |
| Biological specimens | 33 | Plans for collection, laboratory evaluation, and storage of biological specimens for genetic or molecular analysis in the current trial and for future use in ancillary studies, if applicable | Not applicable |

*It is strongly recommended that this checklist be read in conjunction with the SPIRIT 2013 Explanation & Elaboration for important clarification on the items. Amendments to the protocol should be tracked and dated. The SPIRIT checklist is copyrighted by the SPIRIT Group under the Creative Commons "Attribution-NonCommercial-NoDerivs 3.0 Unported" license.

**Item 2b SPIRIT checklist 2013:**

**World Health Organization Trial Registration Data Set**

| Item | | |
|---|---|---|
| 1 | **Primary Registry and Trial Identifying Number**<br>**Name of Primary Registry, and the unique ID number assigned by the Primary Registry to this trial.** | **Clinical Trials:** NCT06296082 |
| 2 | **Date of Registration in Primary Registry** | 6 March 2024 |
| 3 | **Secondary Identifying Numbers** | |
| 4 | **Source(s) of Monetary or Material Support** | The research project is funded by the NEURON ERA-NET 2022 call, supported by the European Union's Horizon 2020 program (GA 964215) and the funding partner organisations: Instituto de Salud Carlos III (ISCIII) through the project "AC22/00016" and co-funded by the European Union-Next Generation EU/PRTR; Fonds de recherche du Québec - Santé (FRQS) #324226; Fonds Wetenschappelijk Onderzoek. |
| 5 | **Primary Sponsor** | University Antwerp, Campus Drie Eiken, Building S Universiteitsplein 1, 2610 WILRIJK (Antwerpen), Belgium. |

| | | |
|---|---|---|
| **6** | **Secondary Sponsor(s)** | Instituto de Investigación Sanitaria de Aragón (IISA)<br>McGill University |
| **7** | **Contact for Public Queries** | pherrero@unizar.es<br>+34646168248, Faculty of Health Sciences, University of Zaragoza |
| **8** | **Contact for Scientific Queries** | PI: Pablo Herrero Gallego, PhD.<br>pherrero@unizar.es<br>+34646168248, Faculty of Health Sciences, University of Zaragoza. |
| **9** | **Public Title** | Comparative study of Dry Needling and Botulinum Toxin for lower limb post-stroke spasticity |
| **10** | **Scientific Title** | Comparative study of the mechanism of action of Dry Needling and Botulinum Toxin type A as a treatment for lower limb post-stroke spasticity: a proof of concept controlled trial (STROKE-POC): |
| **11** | **Countries of Recruitment** | Spain, Canada and Belgium |
| **12** | **Health Condition(s) or Problem(s) Studied** | Lower Limb Post-Stroke Spasticity |
| **13** | **Intervention(s)** | **Arm 1:** Botulinum Toxin type A (BTX A). A (Botox®) Allergan. Reconstitute with: 1 vial of 100 BTX A Units with 4 ml of sterile unpreserved normal saline (i.e., 25 BTX A units per 1 ml normal saline). Normal saline is 0.9% sodium chloride solution. |

| | | |
|---|---|---|
| | | **Arm 2:** Dry Needling (DN). The caliber will be always 0.30 mm and needle length should be determined based on muscle location and depth. The length of the needles will variate from 30,40,50,60 or 75 mm,<br><br>Administration guidance: Localization of the involved muscles for BTX A will be done guided by either ultrasound imaging and/or EMG, according to the clinician´s preference. Localization of the involved muscles for DN, will be done with ultrasound imaging. |
| **14** | **Key Inclusion and Exclusion Criteria** | Inclusion Criteria: 1) aged 18-75 years old, 2) having lower limb post-stroke spasticity in ankle plantar flexors (MAS scores of 1, 1+ and 2); 3) having had a first stroke; 4) have 0 to 12 months evolution since stroke; 5) have no previous Dry Needling (DN) or Botulinum Toxin type A (BTX A) treatment for spasticity; 6) have ankle PROM ≥ 20° (approx.) when the knee is supported in ~30° flexion; 7) be able to ambulate independently with or without aids.<br><br>Exclusion Criteria: 1) medical conditions interfering with data interpretation; 2) any contraindication to receiving BTX A or PS treatment; 3) if taking anti-spasticity drugs, participants should be on stable anti-spasticity medications for at least 3 months without dosage changes during the trial; 4) Pregnancy or breastfeeding. |

**Item 2b SPIRIT checklist 2013:**

**World Health Organization Trial Registration Data Set**

| | | |
|---|---|---|
| **15** | **Study Type** | Observational, Randomized Clinical Trial comparing Botulinum Toxin type A and Dry Needling effectiveness for post-stroke spasticity. Assessors of both arms will be masked. Parallel assignment. |
| **16** | **Date of First Enrollment** | June 2024 |
| **17** | **Sample Size** | • **Number of participants that the trial plans to enroll in total:** 90 (30 per country)<br>• **Number of participants that the trial has enrolled:** 0 |
| **18** | **Recruitment Status** | • **Pending:** participants are not yet being recruited or enrolled at any site. |
| **19** | **Primary Outcome(s)** | **Outcome Name: Spasticity**<br>**Metric/method of measurement:** Tonic Stretch Reflex Threshold and its velocity sensitivity.<br>**Timepoint:** From week 1st to 15th and on week 19th. |
| **20** | **Key Secondary Outcomes** | **Outcome Name and Metric/method of measurement:** : a) muscular architecture and echotexture by ultrasound imaging; b) muscle tone/resistance using Modified Ashworth Scale (MAS); c) gait by instrumented gait analysis, Timed Up and Go and 10 Meter Walk tests; d) Muscle strength with dynamometry; e) quality of life with the EuroQoL questionnaire; and f) cost-effectiveness through an analytic model.<br><br>In addition, we will estimate sample size, recruitment, and consent rates for future |

| | | |
|---|---|---|
| | | randomized controlled trials, as well as treatment safety and feasibility.<br><br>Lastly, to contribute to the improvement of evidence-based practice through a Public and Patient Involvement (PPI) approach, the study will also take into account the patient's preferences and expectations (patient-centered approach).<br><br>**Timepoint:** a) From week 1st to 15th and on week 19th; b) from week 1st to 15th and on week 19th ; c) week 1st,2nd, 9th, 15th and 19th ; d) from week 1st to 15th and on week 19th; e) week 1st,15th and 19th; f) at the end of the study analyzing the cost forms collected from week 3rd to 15th .<br><br>Information related to sample size, recruitment, and consent rates for future randomized controlled trials, as well as treatment safety, feasibility, and Public and Patient Involvement (PPI) approach, will be summarized at the end of the study. |
| **21** | **Ethics Review** | • Status Approved<br>• Date of approval<br>• Name and contact details of Ethics committee(s)<br><br>Spain and Belgium - Commissie Medische Ethiek GZA Ziekenhuizen, Oosterveldlaan 22, 2610 Wilrijk, tel.: 03 443 45 56 or 03 443 38; |

| | | |
|---|---|---|
| | | Canada – Research Ethics Committee of Rehabilitation and Physical Disability (RDP) of the CCSMTL (Integrated university health and social services center for South-Central Montreal). MP-50-2023-1784. Date: 2023-05-30 |
| **22** | **Completion date** | The estimated date to finish the study is in 2026 |
| **23** | **Summary Results** | As this is a protocol, the dates are estimated and will be adjusted according to the progress of the study.<br>• **Date of posting of results summaries:** December 2025.<br>• **Date of the first journal publication of results:** June 2026.<br>• **URL hyperlink(s) related to results and publications:** www.itonuss.eu<br>• **Baseline Characteristics:** age, sex, estudies, weight, height, marital status, living alone, menopause, employment status, tobacco, alcohol, exercise habits, date of stroke, hemiparetic side, use of walking aid, use of antispastic medication, advice on health-related lifestyle changes, visit to a nutritionist after stroke, attendance of physiotherapy sessions for rehabilitation, continuation of treatment or rehabilitation, increase in |

| | | |
|---|---|---|
| | | spasticity in arms, hands, and/or legs since stroke, more pain since stroke, more difficulty controlling bladder or bowel since stroke, if the relationship with family has become more challenging or stressful since stroke.<br><br>Related to communication, if finding it harder to communicate with others since stroke. And lastly, related to cognition if finding it harder to think, concentrate, or remember things since stroke, and if this interferes with activity or participation.<br>● **Participant flow: Information to document the progress and numbers of research participants through each stage of a study in a flow diagram or tabular format:**<br>● **Adverse events: An unfavorable change in the health of a participant, including abnormal laboratory findings, and all serious adverse events and deaths that happen during a clinical study or within a certain time period after the study has ended. This change may or may not be caused by the intervention being studied:** It will be reported at the end of the study.<br>● **Outcome measures: A table of data for each primary and secondary outcome measure and their respective measurement of** |

| | | |
|---|---|---|
| | | **precision (eg a 95% confidence interval) by arm (that is, initial assignment of participants to arms or groups) or comparison group (that is, analysis groups), including the result(s) of scientifically appropriate statistical analyses that were performed on the outcome measure data, if any:** It will be provided at the end of the study.<br>• **Brief summary:** This is a Controlled Randomized Clinical Trial Protocol study to compare the Effectiveness of Dry Needling versus Botulinum Toxin Type A for the Management of Lower Limb Post-Stroke Spasticity. It will be done in 3 different countries. |
| **24** | **IPD sharing statement** | Yes. The data from the STROKE-POC project will be stored in public repositories; the European infrastructure EBRAINS for data management and tools, and the scientific data generated within the project at the "ELIXIR Core Data Resources. |
