# Supplementary material for: Spinal mechanisms and feasibility of Dry Needling versus Botulinum Toxin Type A in post-stroke lower limb spasticity: A proof-of-concept randomized clinical trial protocol (STROKE-POC)
Source: PLoS One. 2026 May 20;21(5):e0334571. doi: 10.1371/journal.pone.0334571 (PMC13189288; doi:10.1371/journal.pone.0334571)
Supplement: S3 File — (PDF) [file pone.0334571.s003.pdf]

Guideline and Full Proposal Application Form

1. Background

Under the umbrella of NEURON, the Network of European Funding for Neuroscience Research established under the ERA-NET scheme of the European Commission (www.neuron-eranet.eu), a joint transnational call (JTC-2022) is now launched. The aim of the call is to facilitate multinational, collaborative research projects that will address translational approaches to **Cerebrovascular Diseases including Small Vessel Disease and Brain Barrier Dysfunction** (see Call Text for further specifications).

2. Proposal submission

Full proposals must be written in English and must be submitted to the Joint Call Secretariat (JCS) by the coordinator through the electronic submitting system exclusively (https://ptoutline.eu/app/neuron_cv)

Full proposals must be submitted by the project coordinator before **the 28th of June 2022 at 14:00 CEST**. Please note that full proposals will only be accepted from applicants explicitly invited by the JCS to submit them.
Please use the template below, and delete the guiding instructions in *italic* font.
Call deadlines are final and will be strictly enforced. The electronic system will not allow submissions after call deadlines. Please take into account that the online data entry may be overloaded by the day of the deadline. It is therefore recommended to upload all the required material well beforehand.

For further information, please contact the NEURON Joint Call Secretariat:

Dr Rajaa Aouache
Agence Nationale de La Recherche (ANR), FRANCE
Phone: + 33 1 72 73 06 73
E-Mail: NeuronCalls@agencerecherche.fr

Checklist for the Coordinator:

In order to make sure that your proposal will be eligible for this call, please collect the information required to tick all the sections below before starting to complete this application form.

- **General conditions:**

☑ The project proposal addresses and is in conformity with national/international regulations regarding human or animal experimentation.

☑ The content of the proposal has not been submitted elsewhere (double funding is not allowed!).

☑ **I declare that I addressed all the detailed information required in the full proposal form**

- **The composition of the consortium:**

☑ The project proposal involves at least 3 eligible project partners from at least 3 different countries participating in the call

☑ The project proposal involves a maximum of 5 eligible research partners (asking for funding as well as participating with own contribution), up to 6 if one of the underrepresented countries listed in Call Text is included.

☑ The project proposal does not include more than two partners from the same country participating in the call.

☑ The coordinator and the partners in the consortium requesting budget are eligible for funding, including new partners added during the widening process.

☑ Changes from pre-proposal were validated by the JCS and respective funding agencies

- **Eligibility of consortium partners:**

☑ I have made sure that each partner involved in the project proposal has checked its eligibility to receive funding by its funding agency (see Country-specific information here: https://www.neuron-eranet.eu/wp-content/uploads/NEURON_JTC2022_All_national_regulations.pdf

Please note:

- Proposals that **do not meet the national eligibility criteria and requirements may be declined without further review**.
- The information given in the pre-proposal is binding. Thus, **any fundamental change between pre- and full proposal stages** concerning the composition of the consortia, objectives of the project or requested budget must be communicated to the Joint Call Secretariat and respective funding agencies with detailed justifications and will only be allowed in exceptional cases by the Call Steering Committee.
- All fields must be completed using **DIN-A4; font: Arial, 10pt; single-spaced, page limits.** Incomplete proposals, proposals using a different format or exceeding length limitations of any sections will be rejected without further review.
- Once completed the proposal must be converted into a **single PDF document** before being uploaded to the submission website.
- Letters of commitment/intent by research partners participating with own budget can be uploaded as pdf on the submission website, if applicable.

In case of inconsistency between the information registered in the electronic submission tool and the information included in the PDF of this application form, the **information registered in the electronic submission tool shall prevail.**

Full Proposal Application Form
Basic Project Data

Acronym (7-10 characters): STROKE-POC

Project Title: Comparative study of the mechanism of action of Dry Needling and Botulinum Toxin type A as a treatment for lower limb post-stroke spasticity: a proof of concept controlled trial

Project Coordinator:

Name	Pablo Herrero Gallero		
Institution/Department	IIS ARAGON. Instituto de Investigación Sanitaria Aragón		
Position	Researcher. Head of iHealthy research group		
Address	Avda. San Juan Bosco, 13		
Country	Spain		
Phone + Fax	+134 646168248		
Email	pherrero@unizar.es		
ORCID Number	0000-0002-9201-0120	ECR[1] (yes or no)	NO

Partners:

No.	Country	Name of the group leader	Institution and full affiliations (e.g. address, phone + fax, e-mail, ORCID number)	ECR (yes or no)
2	Canada	Mindy F. Levin	McGill University.(MGU)<br>Affiliation:<br>- Department: School of Physical and Occupational Therapy, Faculty of Medicine and Health Sciences<br>- Address: 3654 Promenade Sir William Osler, H3G 1Y5. Montreal, Quebec. Canada.<br>- Phone number: +1 514-398-3994<br>- e-mail: mindy.levin@mcgill.ca<br>- ORCID: 0000-0002-8965-7484	NO
3	Belgium	Wim Saeys	University of Antwerp (ANT)<br>Affiliation:	NO

[1] Early Career Researcher

			- Department of Rehabilitation sciences and Physiotherapy, Faculty of medicine and Health Sciences.<br>- Address: Universiteitsplein, 2610. Wilrijk, Belgium<br>- Phone number: +32 496 80 43 47<br>- e-mail: wim.saeys@ANTen.be<br>- ORCID: 0000-0001-8193-5016	

Total funding applied for: **755 249€** €

- **Scientific abstract of the project** (max. 1/2 page)

Rationale: Cerebrovascular Accident (CVA) is one of the main causes of morbidity and disability worldwide. A frequent consequence of stroke is spasticity in the affected limbs. Spasticity is *a velocity-dependent increase in muscle reflex activity that affects both resting muscle tone (hypertonicity) and voluntary movements.* Lower limb spasticity is associated with limitations in walking ability, resulting in an increased incidence of falls, a reduced quality of life and greater caregiver burden. Two treatments for post-stroke spasticity are injection of botulinum neurotoxin (BTX A) and dry needling (DN) to chemically or mechanically disrupt signal transmission at the neuromuscular junction in the affected muscle respectively. Both treatments reduce spasticity and improve functional walking. As BTX A injection has some adverse effects, DN may be an effective, minimally-invasive, non-pharmacological alternative to the more invasive chemical denervation. However, while some local (muscle) mechanisms of action of BTX A and DN have been described, there is little information about their actions at the central (spinal) level, on activity, quality of life and cost-effectiveness.
Objectives: The **primary objective** is to determine the mechanisms of action of BTX A infiltration and DN on lower limb post-stroke spasticity at the central (spinal) level. The **secondary objectives** are to determine safety and feasibility of each treatment and their effects at muscle and functional levels, quality of life and cost-effectiveness. The **primary hypothesis** is that DN treatment will be comparable to BTX A and will decrease post-stroke spasticity by decreasing stretch reflex excitability at the central (spinal) level. Improving knowledge of the mechanisms of action of both interventions will lead to more informed treatment prescription and better clinical mobility outcomes for post-stroke patients. **Methods:** This prospective study will compare BTX A and DN treatment on spasticity relief in people who have sustained a first stroke 3-12 months previously and who have plantar flexor spasticity. Ninety patients will be recruited from 3 centers (30 per site) in Spain, Belgium and Canada. We will use a multiple-baseline time-series design across pairs of subjects matched for age and time since stroke. BTX A will be injected once and DN will be applied once weekly for 12 weeks. Effects will be evaluated before, during and after treatment and at a 4 week follow-up by blinded evaluators. Effects on spasticity will be evaluated at the central (spinal) level using a physiological measure of motoneuronal excitability (Tonic Stretch Reflex Threshold and its velocity sensitivity) and at the muscle level by quantifying morphological changes with ultrasound imaging and the perceived resistance to stretch (Mod-Mod Ashworth Scale). We will also assess effects on gait (Timed Up and Go, 10 Meter Walk Test and instrumented gait analysis) and quality of life (EuroQOL-5D). Cost-effectiveness of each intervention will be determined. A patient Advisory Group will be created to engage patients by asking for feedback during the study and to contribute to results dissemination. **Outcomes:** Although BTX is the gold standard for post-stroke spasticity treatment, DN treatment has shown to be effective with potentially fewer adverse effects. However, DN has not yet been routinely implemented in clinical practice and there are no comparative studies with BTX A and mechanisms of action remain unknown. This impedes prescription of the best available treatment to post-stroke patients considering spasticity pathophysiology. Results of this feasibility study (proof of concept) will determine the mechanisms of action of both treatments so that future studies with larger samples and other neuropathologies can be developed.

- **Lay Abstract** (max. 1/2 page)

Stroke is one of the main causes of disease and disability in the world and has a significant economic impact on society. As populations grow and people live longer, the number of cases of stroke is expected to rise. Stroke affects the central nervous system and interferes with the ability to move and walk. Half of all stroke victims develop spasticity (stiffness in the muscles) within six months, which may cause discomfort and interfere with the ability of people to return to everyday life activities. There are several treatment possibilities for alleviating spasticity. One of the most effective treatments is the injection of Botulinum Toxin type A (BTX A). However, this treatment is considered to be ‘invasive’ and it has been linked to several side effects. A relatively new treatment for spasticity with comparable effectiveness as BTX A, is a technique called ‘dry needling’. This technique does not require the use of drugs and is safe, minimally invasive and has fewer side effects. However, the use of dry needling is not very common in current clinical practice. Some studies have reported the effects of these two techniques on the muscle and on the patients’ ability to move but their effects across the whole system have not been systematically studied and there are few comparative studies of their effects. ***We propose*** that dry needling and BTX A treatments will have comparative effects on decreasing spasticity but that dry needling will have fewer side effects and be more acceptable as a treatment option for patients and their families. We will study this question by examining the effects of each treatment in ankle muscles in two groups of subjects. One group will have a series of 12 dry needling sessions and the other will have one injection of BTX A. We will evaluate the effects of the treatment on spasticity at several different levels –muscle, reflex activity, motor ability, quality of life and whether the treatment is found to be acceptable and cost-effective. We will use novel methods to measure the effects that will provide us with new information to help clinicians together with patients and their families make more informed choices about spasticity treatment options.

Detailed Information

1. Background and present state of the art in the research field, and rationale

Cerebrovascular Accident (CVA) is one of the main causes of morbidity and disability worldwide (Feigin et al., 2021). The Global Burden of Diseases concluded that there are 101 million cases of stroke in the world (Feigin et al., 2021). In Europe, spasticity affects ≈1.1 million inhabitants each year, and at the beginning of the 21st century, the age-standardized incidence of stroke ranged from 95 to 290/100,000 per year (Béjot et al., 2016). Moreover, as populations continue to grow and live to an older age, the number of cases in the European Union (EU) is expected to increase by 27% between 2017 and 2047 (Wafa et al., 2020). According to a cost analysis study published in 2020, the total economic cost for the EU's 28 member states in 2017 was €57 billion (Luengo-Fernandez et al., 2020).

Within the first six months, 43.2% to 49.5% of stroke patients develop spasticity (Dorňák et al., 2019), “a velocity-dependent increase in muscle reflex activity that affects both resting muscle tone and voluntary movements”. Spasticity is clinically manifested as a feeling of resistance to externally imposed muscle stretch beyond a certain joint angle, rising rapidly with increasing speed and varying with the direction of joint movement. Spasticity interferes with movement and may be associated with discomfort or pain, joint contracture, and abnormal limb posture (Thompson et al., 2005). One of the most frequent muscles affected are the ankle plantar flexors, with ankle spasticity being a major barrier to gait recovery due to equinovarus foot deformity. This leads to reduced foot clearance and circumduction during gait, asymmetric weight-bearing (Foley et al., 2010), and prolonged hospitalization (Dajpratham et al., 2009). Indeed, more severe spasticity is related to decreased functional independence (Oh et al., 2018), greater tendency to fall and reduced quality of life (Gupta et al., 2018; Soyuer & Öztürk, 2007). According to the American Stroke Association, 63% of occupational and physical therapists reported post-stroke spasticity as a key reason for patients failing to meet treatment goals (American Stroke Association, 2022).

Currently, two treatments used to decrease spasticity in patients with stroke are botulinum toxin type A infiltration (BTX A) (Varvarousis et al., 2021) and dry needling (DN) (Fernández-de-Las-Peñas et al., 2021). Whereas BTX A infiltration is considered the gold-standard to treat spasticity (Franeschini et al. 2014; Varvarousis et al., 2021) and has been widely used in clinical practice since 1989 (Das & Park, 1989), the use of DN in clinical practice is still limited, despite having been demonstrated as safe (Boyce et al., 2020). Regarding the effectiveness of DN for post-stroke spasticity, a recent meta-analysis concluded that there is moderate evidence for a positive effect of dry needling on spasticity in the lower extremity in post-stroke patients (Fernández-de-Las-Peñas et al., 2021), although the number of clinical studies is limited and very heterogeneous. Apart from reducing spasticity, BTX A has been shown to be effective in improving functional gait, measured with the 10 Metre Walk Test (10MWT) (Varvarousis et al., 2021). DN has also been shown to effectively reduce spasticity and improve functional gait in a smaller number of studies (Hadi et al., 2018; Ghannadi et al., 2020).

DN is expected to be a safer treatment, with fewer side effects than BTX A injection. Several adverse effects have been reported due to the use of BTX A for spasticity management (Simon & Yelnik, 2010). In a recent review of the changes in muscle structure and passive mechanical properties, BTX A injection led to a lingering atrophy, with a remodeling of the muscle contractile proteins (process of fibrosis), which may not be completely reversible, as well as changes in muscle elasticity (Mathevon et al., 2015). In contrast, no similar adverse effects have been reported for DN. After BTX A injection, histological analyses in animals show that a neurogenic atrophy systematically occurred (Schroeder et al., 2009). In humans, one year after a single injection, histological recovery remained incomplete (Schroeder et al., 2009). Furthermore, ultrasound analyses of muscle fibers showed a reduction of gastrocnemius thickness and pennation angle (Tok et al. 2011) and magnetic resonance imaging volumetric analysis indicated persistent muscular atrophy six months and one year after a single BTX A injection (Schroeder et al., 2009; Mathevon et al. 2015). Moreover, BTX A and DN differ in the types of needles used during the treatment. BTX A is infiltrated with a 26 gauge (0.45 mm) beveled needle, while DN is performed with a thinner (from 0.25 to 0.30 mm caliber), filiform, solid non-beveled needle, similar to those used for acupuncture, which has shown to lead to less damage at the muscle level (Figure 1, Bosque et al, in press).

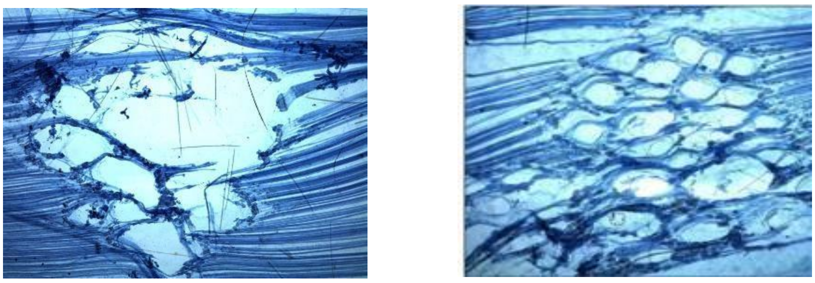

Figure 1. Muscle damage in a myofascial trigger point (MTrP) generated in a mouse through infiltration of neostigmine. After creation of the MTrP (contraction knots), the damage provoked by a beveled needle similar to the one used for BTX A injection is shown on the left and that caused by a filiform needle, like the ones used for DN is shown on the right.

While some of the local mechanisms of action of both DN and BTX A are known, the mechanisms through which they decrease spasticity at central levels remain unknown. Current evidence shows that both DN and BTX A work on the same target structure, the neuromuscular endplate zone, but via different mechanisms of action. BTX A causes a chemical denervation (McGuire, 2015) whereas DN provokes a mechanical disruption of dysfunctional endplates (Domingo et al., 2013). However, few studies have reported how either of these treatments affect excitability at the spinal level, and their effects on muscle function, mobility and quality of life.

Regarding costs, BTX A treatment involves high costs (between 603,64 € and 707,59 € annual costs per patient + medical/nursing staff costs in Spain;Hernández Herrero & Miangolarra Page, 2021). Annual costs for DN are estimated to be 83.2€ per patient (Fernández Sanchis et al., 2022a,b) + physiotherapy staff costs. Both BTX A treatment (1 session) or DN treatment (multiple sessions) are complemented with standard physiotherapy care. Until now, there have been no comparative studies of DN with BTX A that allow reaching any conclusions in terms of costs and cost-effectiveness. Two studies carried out in post-stroke upper-limb spasticity concluded that DN treatment was cost-effective based on the percentage of responders according to a clinical measure of muscle resistance and the fact that DN is inexpensive (Fernández-Sanchis et al., 2022a,b). However, no studies have analyzed the cost-effectiveness of DN in lower limb post-stroke spasticity or compared costs to BTX A injection. Moreover, the number of sessions necessary for each treatment to be cost-effective has not been determined. One study in subacute stroke patients (Fernández Sanchis et al., 2022a) concluded that 4 weeks could be more cost-effective than 8 weeks of DN treatment.

In terms of applicability to clinical practice, the infiltration of BTX A is usually done by a physician in most countries in contrast to DN, which is performed mainly by physiotherapists. Thus, DN may be more accessible to patients in rural areas with a limited presence of medical specialists to provide BTX A infiltration. Moreover, considering a patient-centered approach, there are no alternatives to BTX A infiltration that have a similar effectiveness for the reduction of spasticity using non-pharmacological treatments, which should be also taken into account as many patients would potentially prefer a non-pharmacological treatment.

Rationale:

Stroke is one of the main causes of morbidity and disability. No drug currently available completely alleviates spasticity and many have undesirable side effects, such as fatigue, drowsiness (Chan, 1990; Goldstein, 2001; Hulme et al., 1985; Jamous et al. 1994) and weakness (Lapeyre et al. 2010). Clinical practice guidelines recommend the use of BTX A infiltration to diminish spasticity, despite its adverse effects. DN is an alternative, less-invasive treatment for the management of spasticity. The use of DN for spasticity management may improve access to health care by increasing the treatments available to patients. Access to DN would be easier for patients, as this is a treatment that can be provided at any clinical center where there is a physiotherapist, in contrast to BTX A, which is only delivered in specialized clinical centers in most countries. Availability of DN could not only increase accessibility to the treatment of spasticity but also decrease treatment costs for patients and their families, as well as the health system.

However, there are no comparative studies between DN and BTX A that can be used to help clinicians and patients make informed decisions about their use. Indeed, no studies have analyzed and/or compared their mechanism(s) of action at different levels in order to understand to which patients each treatment should be administered to maximize their effects. A better understanding of the mechanism of action of treatments for spasticity may help in patient stratification as well as allow a better personalization of treatment taking into account the pathogenesis of spasticity and the different mechanisms that can be effective to modulate it.

2. Work plan

A. Objectives, hypotheses and evidence

The **primary objective** is to determine the mechanisms of action of DN and BTX A infiltration treatments on lower limb post-stroke spasticity at central (spinal) levels. ***Primary hypothesis:*** *DN and BTX A infiltration treatments will decrease spasticity by decreasing stretch reflex excitability as measured by an increase in the Tonic Stretch Reflex Threshold (TSRT) angle, a decrease in velocity sensitivity (mu) of the stretch reflex, and an increased muscle resistance to stretch*

The **secondary objectives** are to determine:

1) the safety and feasibility of DN and BTX A infiltration treatments for lower limb post-stroke spasticity. ***Hypothesis 1.1:*** *DN will have fewer adverse effects than BTX A and will have similar feasibility.*
2) the mechanisms of action and effects of DN and BTX A infiltration treatments on lower limb post-stroke spasticity at the muscle level. ***Hypothesis 2.1:*** *DN will have similar effects as BTX A on morphometric variables (i.e increased thickness and pennation angle;* Blazevich et al. 2006) *and densitometric variables (characterization of histogram distribution pre-post by echo-textural analyses), as measured with ultrasound, despite having different mechanisms of action.*
3) the mechanisms of action and effects of DN and BTX A infiltration treatments on lower limb post-stroke spasticity at functional levels. ***Hypothesis 3.1:*** *DN will have similar effects as BTX A on gait function measured with clinical scales and instrumented gait analysis.*
4) the effect of DN and BTX A infiltration treatments on quality of life and cost-effectiveness. ***Hypothesis 4.1:*** *DN will have a similar impact as BTX A on the patients' quality of life.* ***Hypothesis 4.2:*** *The cost-effectiveness of both treatments will be comparable considering that the administration procedure is different (BTX A: one infiltration every 3 months vs DN: 12 treatments over three months) due to their different mechanisms of action.*

Significance of the study:

Although BTX A is the gold standard for the treatment of post-stroke spasticity, new treatments, such as DN, have also been shown to be effective for post-stroke spasticity with potentially fewer adverse effects. However, DN has not yet been fully implemented in clinical practice as there are no comparative studies with BTX A and the mechanisms of action remain unknown. If the mechanisms of action at different levels of both DN and BTX A are better known, treatment prescription for individual patients will be improved, and will lead to better future patient stratification, ultimately resulting in better clinical mobility outcomes.

Results of this feasibility study (proof of concept) will determine the mechanisms of action of both treatments and it will allow us to obtain initial estimates for sample size calculations for future randomized controlled trials. It will also delineate the difficulties and challenges that may occur in the implementation of the treatment in different countries, helping to identify region specific and/or institution-specific practices that can have an impact on overall study completion.

Moreover, in terms of translation to clinical practice, although the POC study is directed to the management of spasticity in people with stroke, spasticity is a also a common characteristic of many other neurological conditions, so that any advances in the understanding of the mechanisms of action of DN in stroke may be translated to other neurological conditions such as Parkinson Disease, Multiple Sclerosis, Spinal Cord Injury and Traumatic Brain Injury amongst others, where anecdotal cases have been reported about the benefits of DN on spasticity.

It is important to mention that alternative approaches could be researched in the future based on the results of this POC study. One possibility would be to analyze the cost of DN if this is included in the standard physiotherapy treatment plan without the need of additional sessions, which could involve additional savings.

Evidence:

DN is effective in decreasing myofascial pain (De Sire et al. 2021; Khan et al., 2021; Rodríguez-Huguet et al. 2022) and is a safe technique. A case report in 2010 was the first study to report the effects of DN in a child with spastic cerebral palsy (Herrero Gallego & Del Moral, 2010). It was followed by different clinical cases and case series studies on the effects of DN on spasticity due to different pathologies. Since 2014, three studies

(Ghannadi et al., 2020; Salom-Moreno et al., 2014; Sánchez-Mila et al., 2018) have analyzed the effect of DN in lower limb post-stroke spasticity, with a meta-analysis concluding that "moderate evidence suggests a positive effect of dry needling on spasticity in the lower extremity in post-stroke patients" (Fernández-de-Las-Peñas et al., 2021).

The clinical studies carried out to date have only measured the effects of DN and BTX A on spasticity with clinical scales and therefore, the mechanism(s) of action is still unknown. Comparative studies of the mechanisms of action of DN and BTX A are necessary in order to determine the magnitude and duration of their effects at both muscle and central levels. It is expected that information about the action of DN and BTX A will be gained by measuring the effects on the TSRT angle and the velocity-sensitivity of the stretch reflex (μ). The TSRT is the joint angle at which spasticity in the muscle begins (Figure 2A; Levin & Feldman, 1994; Levin et al. 2000). In healthy individuals, no TSRT can be evoked in resting muscles because the TSRT is increased so that it lies outside of the biomechanical joint range (Figure 2B, TSRT+).

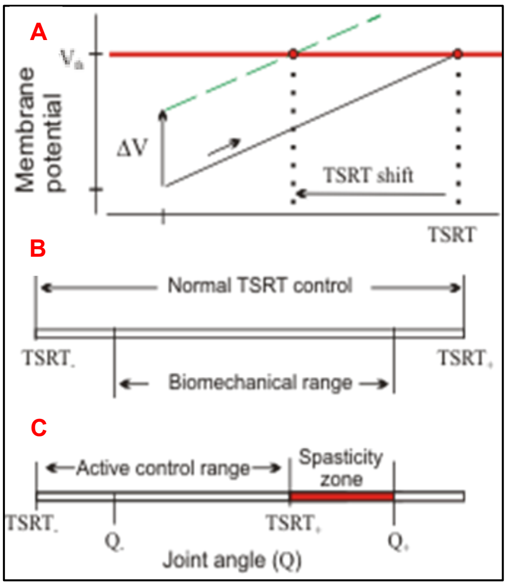

Figure 2 (A, B, C). Descending input enhances (Δv) the membrane potential such that the same amount of stretch (diagonal arrow) leads to a stretch response at a shorter muscle length. Normal (B) and reduced (C) range of TSRT.

However, after CNS injury, deficits in descending and spinal mechanisms together with changes in intrinsic motoneuronal properties (e.g.,chloride reversal potentials, serotonin receptor properties, Nichols & Steeves, 1986) may contribute to limitations in TSRT regulation so that the TSRT at rest lies within the biomechanical joint range (Figure 2C). This has been shown to lead to both spasticity and disordered muscle activation (Levin et al. 2000). In healthy subjects, only phasic responses are evoked during rapid stretch of passive plantar flexors (Levin et al., 2000). In contrast, in spasticity, muscles cannot relax such that stretching leads to muscle activation beyond a certain muscle length or joint angle evoking a tonic response in the muscle (Figure 2C, Spasticity Zone lying beyond the TSRT+), resulting in active resistance, clinically identified as spasticity. Our preliminary studies have shown that TSRT and μ differentiate between stroke-related spasticity and Parkinsonian rigidity (Mullick et al. 2013), determine the influence of heteronymous muscle activity on the angular zones in which spasticity is present (Musampa et al. 2007), and identify ranges in which movements can/cannot be voluntarily controlled (Subramanian et al., 2018). Also, the ability to modulate TSRTs and μ during voluntary movement was related to levels of functional impairment (Turpin et al. 2017). Since the TSRT and μ are determined by synaptic influences from afferents on alpha motoneurons in the spinal cord, changes in these measures may track spasticity-relieving treatment effects as well as predict the eventual level of sensorimotor recovery.

At the muscle level, both DN (Hadi et al., 2018) and BTX A (Tok et al., 2011) have led to improvements in different muscle parameters (pennation angle and muscle thickness) of the gastrocnemius medialis, measured with ultrasound, although in the case of BTX A, adverse effects have been reported and they still have to be analyzed in the long-term (Mathevon et al., 2015). However, recently, echo-textural analysis using ultrasound imaging has provided the opportunity to correlate the specific characteristics of the tissue with other functional outcomes. Echo-textural analysis measures the relationship between muscle characteristics (Figure 3) and has led to the description of new biomarkers in neurological diseases such as amyotrophic lateral sclerosis (Martínez-Payá et al., 2017) for diagnosis and evaluation. This computational image analysis is a reliable method (Del-Canto-Fernández et al., 2022) and could help to determine the mechanism of action of both DN and BTX A at the muscle level.

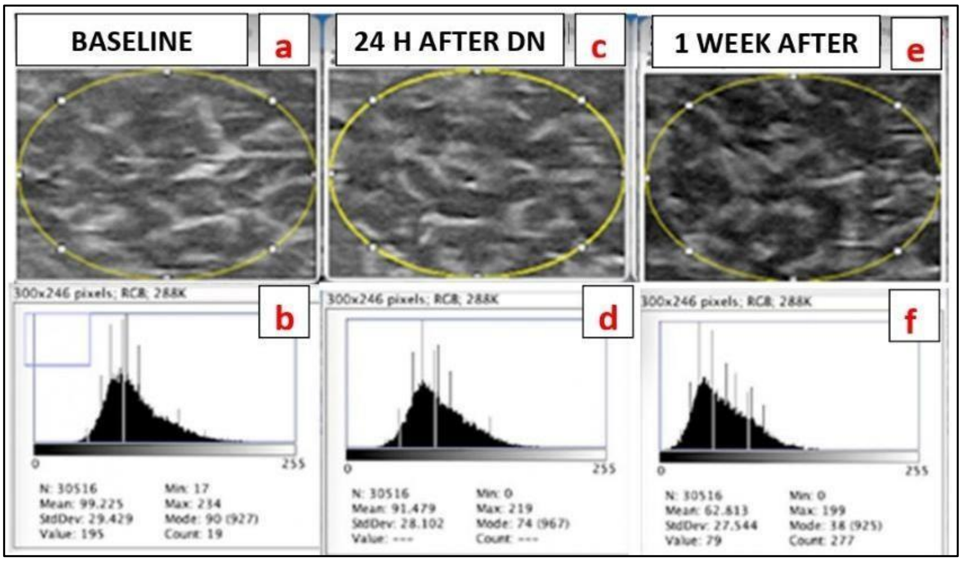

Figure 3. Ultrasound images of gastrocnemius medialis at baseline, 24 h, and 1 week after dry needling (non-published data). Figure 3a shows the region of interest that has been analyzed in figure 3b (baseline). Figure 3c shows the region of interest that has been analyzed in figure 3d (24 h after dry needling). Figure 3e shows the region of interest that has been analyzed in figure 3f. In the first row (figures 3a,3c,3e) it can be seen how the region of interest becomes less echogenic after dry needling. In the second row, the quantitative analysis of the regions of interest (figures 3b, 3d and 3f) shows the distribution of grey level (horizontal axis) and the quantity of pixels for each grey level (vertical axis). As it can be seen in the quantitative analysis (figures 3b, 3d and 3f), there is an increase in the amount of pixels showing less echogenicity (more black) after dry needling throughout the time.

At the functional level, there is limited evidence supporting the effectiveness of DN on gait in people with stroke (Ghannadi et al., 2020), and it is unknown whether these improvements were due to changes at central or muscle levels. Combining TSRT, μ, clinical, muscle ultrasound and gait analysis measures is expected to provide new information on the influence of DN and BTX A at the functional level.

B. Relevance

Whereas research with pharmacological treatments follows a strict process before clinical uptake, this is not always the case for physiotherapeutic treatments. Many physiotherapeutic treatments come from the history of different civilizations that applied physical agents and natural remedies to treat different conditions. This is the case of DN, derived from ancient acupuncture techniques which evolved thanks to an accidental discovery from the use of injections to treat muscle pain, when Brav and Sigmond in 1941 (Brav & Sigmond, 1941) claimed that pain could be relieved by simple needling without the injection of any substance. Since then, the use of DN has been extended worldwide, with an exponential increase in the number of publications in the last few years, showing that DN is effective to decrease pain without the need of injecting any pharmaceutical substances. Although DN has been demonstrated as safe and some mechanisms of action for pain are already known, the mechanisms of action for the management of spasticity have not been clearly elucidated. We consider that the this POC study is highly relevant for the following reasons:

- Cerebrovascular Accident (CVA) is one of the main causes of morbidity and disability worldwide. The Global Burden of Diseases concluded that there are 101 million cases of stroke in the world and, in Europe, it

affects ≈1.1 million inhabitants every year. Moreover, as populations continue to grow and live to an older age, the number of cases in the European Union (EU) is expected to increase.

- This POC study is expected to improve understanding of mechanisms of action of both DN and BTX A at muscle, central and functional levels. This should help both health professionals and the health system to know when to offer DN and if DN can be offered as complementary/alternative treatment to BTX A.

- A better understanding of the mechanisms of action, duration of effects and other characteristics of DN and BTX A will contribute to providing better information to patients so that they can make more informed decisions, which will contribute to better patient-centered care.

- The increase of available treatments derived from the knowledge gained of the mechanisms of action is good for patients and their families, as it allows them to choose between different treatments according to their preferences. We consider that this will also be advantageous for some patients, who could be offered a new treatment option without the need of injecting pharmacological substances.

- According to the existing data of safety and adverse effects, DN is expected to be safer than BTX A due to the type of needle used and the lack of injection of pharmacological agents. Moreover, DN has not shown any adverse effects derived from repeated application as it is the case of BTX A, which may lead to negative long-term effects.

- DN treatment is more accessible, as it can be provided by physiotherapists without the need of a specialist medical doctor, as it is the case for BTX A injection. Especially for patients in rural areas, it increases accessibility and also decreases the inconvenience to their families that in some cases have to transport or accompany the patients to the health center. This is also expected to have an impact in costs, due to costs of transportation (transport and staff) and days of work lost.

- In terms of translation to clinical practice, DN has a great potential, as this is a technique commonly used by many physiotherapists mainly for the treatment of pain and musculoskeletal conditions, which could be integrated into the standardized physiotherapy treatment for neurological patients. Moreover, it is important to consider that spasticity is common to many different neurological conditions, so the results of this study could be translated to other neurological conditions such as Parkinson Disease, Multiple Sclerosis, Spinal Cord Injury or Traumatic Brain Injury, amongst others, where anecdotal cases have been reported about the benefits of dry needling on spasticity.

Figure 4. Timeline for outcome measurements

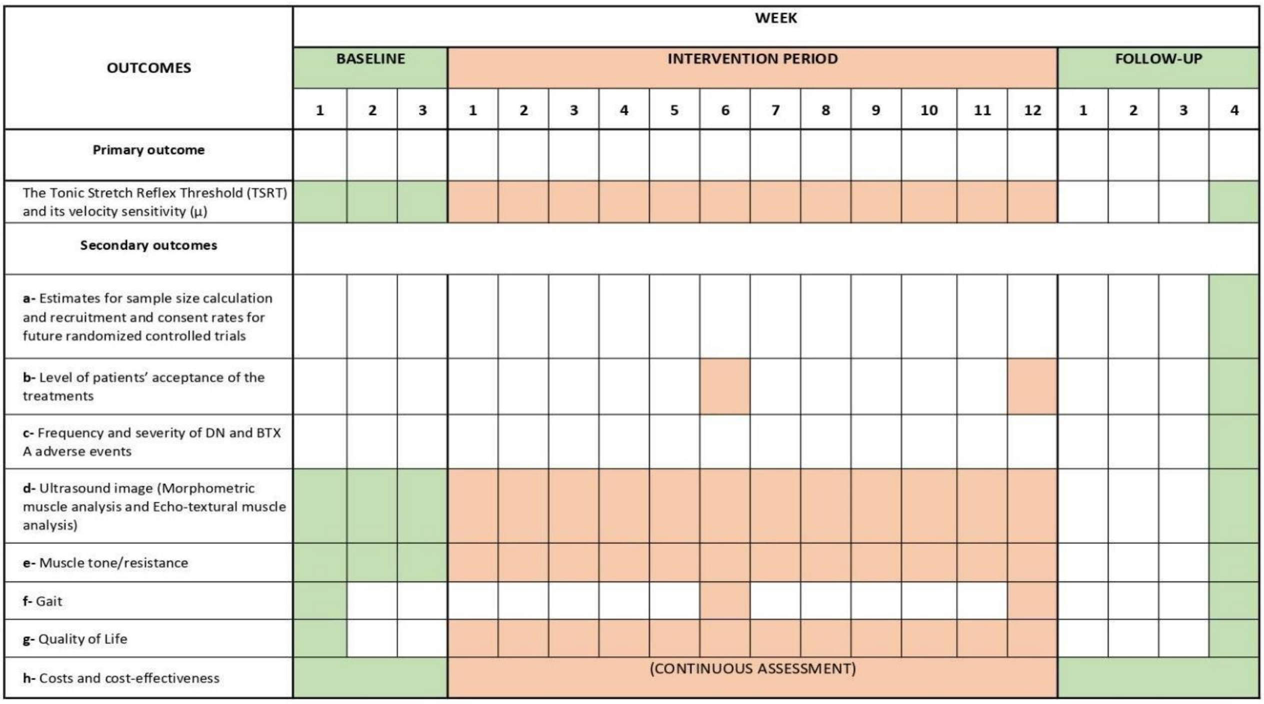

Methodological approach
Primary outcome:

The primary outcome will be measured at baseline (3 baseline measurements to establish test-retest reliability for this population) and then, weekly for 12 weeks, with a short-term follow-up evaluation after an additional 4 weeks.

- The Tonic Stretch Reflex Threshold (TSRT) and its velocity sensitivity (µ) (Figure 5): TSRT and µ are novel measures of stretch reflex excitability that provide an indirect indicator of the excitability of α-motoneurons at the level of the spinal cord (Levin & Feldman 1994; Blanchette et al. 2016). Measures of TSRT angles in ankle plantar flexors in patients with stroke showed high inter-rater reliability (ICC=0.85, 95% CI=0.703–0.928; p<0.001). Paired t-test revealed no significant differences in either TSRT angles (mean difference = 6.1±4.3º) or µ values (mean difference = 0.034±0.015s) between evaluations (Blanchette et al., 2016). The TSRT mean increase of 6.1º suggests that a change in plantar flexion TSRT angle of ~10% of the joint range (normal ankle range is ~70º) would be needed to show the effect of reducing activity in spinal α-motoneuronal activity of a treatment intervention. The TSRT and µ will be measured using the Montreal Spasticity Measure (MSM; Calota et al. 2008). The MSM is a portable device consisting of a 2-channel EMG system and an electrogoniometer. An algorithm is in place to recognize the stretch responses and compute the TSRT and µ based on a minimum of 20 muscle stretches performed at various software-instructed velocities by the evaluator. In this methodology, it is important to note that velocity is not strictly controlled since we are not concerned with reproducing the same velocity with each stretch. Indeed, a key feature of the method is that the greater the variability of stretch velocities, the more robust is the determination of TSRT. This is because the TSRT is computed as the angle at which abnormal muscle activation begins in the muscle at rest. Since the activity at rest cannot be measured directly, the TSRT is determined by computing the regression equation between a series of stretch velocities and the angles corresponding to the onset of the stretch response in the stretched muscle (Blanchette et al., 2016; Calota et al., 2008). The intersection of the regression line with the zero velocity value is the TSRT angle and the inverse of the slope of the regression line is the velocity sensitivity, µ. Stretch velocity is not strictly controlled. Indeed, a more robust regression is obtained when there is greater variability in the velocity/angle data.

For this measure, a higher value of TSRT and a lower value of µ indicate improvements (diminished) spasticity. This is related to the **primary hypothesis***: DN and BTX A infiltration treatments will decrease spasticity by decreasing stretch reflex excitability as measured by an increase in the Tonic Stretch Reflex Threshold (TSRT) angle and a decrease in velocity sensitivity (mu) of the stretch reflex.*

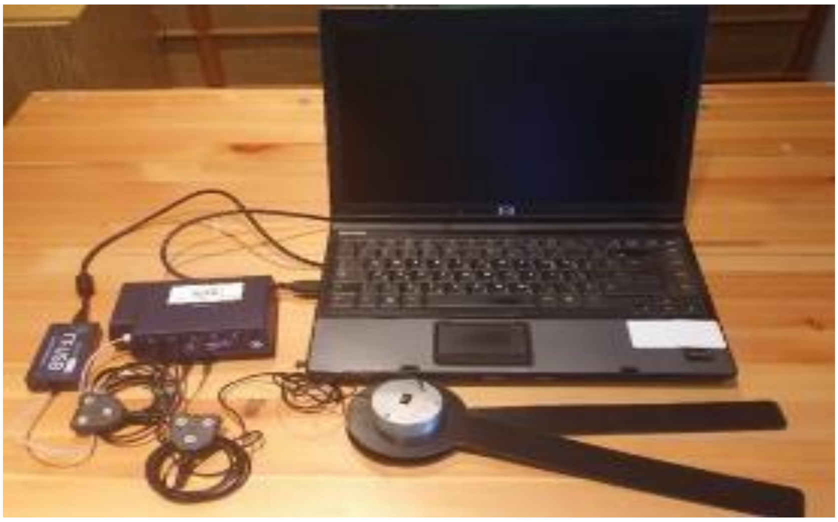

Figure 5. The Montreal Spasticity Measure (MSM) consisting of a 2 channel EMG system (Procomp EMG amplifier) and a custom-made goniometer with a high precision resolver. A computer algorithm displays real-time data and instructs the evaluator on the sequence of stretch velocities. It then computes the TSRT and µ in real-time.

Secondary outcomes:

a- **Estimates for sample size calculation and recruitment and consent rates for future randomized controlled trials** will be measured at the three clinical sites to identify determining factors. The drop-out rate during the treatment and follow-up will also be analyzed for each group (DN and BTX A). This will be determined at the end of the study. This is related to Hypothesis 1.1 (*DN will have fewer adverse effects than BTX A and will have similar feasibility).*

b- Level of patients' acceptance of the treatments will be determined by a questionnaire designed at the beginning of the study as part of the patient and public involvement methodology. Patient acceptance will be measured after 6 wk and 12 wk of the intervention and at the follow-up assessment. This is related to Hypothesis 1.1 (*DN will have fewer adverse effects than BTX A and will have similar feasibility).*

c- Frequency and severity of DN and BTX A adverse events. This will be determined at the end of the study. This is related to Hypothesis 1.1 (*DN will have fewer adverse effects than BTX A and will have similar feasibility).*

d- Ultrasound imaging: The images obtained will be analyzed in two different ways: firstly a **morphometric muscle analysis** and secondly a **echo-textural muscle analysis.** Data will be acquired 3 times at baseline to establish reliability in the present patient cohort and then, weekly for 12 weeks and at follow-up after 4 weeks**,** as it is necessary to analyze which muscle changes occur at the different timepoints**.** This is related to Hypothesis 2.1 (*DN will have similar effects as BTX A on morphometric variables and densitometric variables, as measured with ultrasound).*

d.1 Morphometric muscle analysis: morphometric variables (i.e muscle thickness, pennation angle) will be measured with ultrasound. Measurement of morphometric variables has been shown to be reliable in neurological patients (Mathevon et al., 2017; May et al. 2021).

d.2- Echo-textural muscle analysis (see Figure 3): An echo-textural analysis of muscle microstructure at defined regions of interest (ROIs; Yang et al., 2020) will be done with FIJI software (Schindelin et al., 2012). Echo-texture analysis has excellent intra-rater reliability (Chang et al. 2018; Ríos-Díaz et al., 2019) and may provide biomarkers of the severity of muscle pathology. This mathematical analysis will be divided into four different sub-analyses: Grey Level Co-occurrence Matrices (GLCM), Grey Level Run-Length Matrices (GLRLM), Local Binary Pattern (LBP) analysis and Blob analysis. ROIs will be selected from the images acquired by the morphometric analysis. Usually ultrasound imaging offers between 0-255 grey levels with each pixel having a value of 1. We will quantify the relationship between the pixel distribution and the type of intervention, as well as functional outcomes.

- Grey Level Co-occurrence Matrices (GLCM), which consist of comparing pairs of pixels separated by a certain distance (by default a value of 1 is used) and in an angular direction (0°, 45°, 90°, and 135°) along the entire matrix, calculating the frequency with which certain grey levels appear in the image and their relationship with each other.
- Grey Level Run-Length Matrices (GLRLM), calculated from the run-length statistic, which represents a set of consecutive pixels having the same grey level in each of the four angular directions described across the entire matrix.
- Local binary pattern (LBP) analysis, which compares the intensity of a central pixel, which is taken as a reference value, with the surrounding pixels.
- Blob analysis, based on detecting areas close to each other with a similar eco-intensity called "blobs".

e- Muscle tone/resistance, measured with the **MMAS (Modified Modified Ashworth Scale**, Ghotbi et al., 2011), which is a clinical instrument for measuring spasticity. It is widely used in research, in different patient groups such as stroke. MMAS scores spasticity on an ordinal scale from 0 to 4 as follows: 0 = no increase in muscle tone; 1 = slight increase in muscle tone, manifested by a catch and release or by minimal resistance at the end of the range of motion when the affected part(s) is moved in flexion/ extension; 2 = marked increase in muscle tone, manifested by a catch in the middle range and resistance throughout the remainder of the range of motion, but affected part(s) easily moved; 3 = considerable increase in muscle tone, passive movement difficult; and 4 = the affected part(s) rigid in flexion or extension. The MMAS has shown good intra-rater reliability when used to measure ankle plantar flexor spasticity in stroke patients (Ghotbi et al., 2011). A higher score on the MMAS represents increased spasticity. **MMAS** will be measured 3 times at baseline to establish its reliability in the present patient cohort and then weekly for 12 weeks and at follow-up after 4 weeks.

This is related to Primary Hypothesis: DN and and BTX A infiltration treatments will decrease spasticity by decreasing stretch reflex excitability as measured by an increase in the Tonic Stretch Reflex Threshold (TSRT) angle, a decrease in velocity sensitivity (mu) of the stretch reflex, and an increased muscle resistance to stretch.

f - Gait: Functional gait and muscle force will be measured using clinical scales TUG, (Chan et al., 2017; Podsiadlo & Richardson 1991), 10MWT, (Cheng et al., 2019) and instrumented gait analysis (Yavuzer et al., 2008). Faster times for the TUG and 10MWT constitute improved function at the activity level. However, although increased gait speed is important in daily life (e.g. crossing streets), it is not entirely related to quality parameters such as gait symmetry. Therefore, in addition to the clinical scales, instrumented gait analysis will be conducted to identify and explain the underlying mechanisms of the clinical outcomes observed in this study. For instrumented gait analysis, we will use a combination of sensorized insoles, which provide spatio-temporal parameters of gait in a real scenario and gait analysis in a lab, where the variables of interest will be spatial and temporal 3D kinematics of ankle, knee and hip joints and step-time measurements such as % stance time, % swing time, % single stance support, % double stance support, stride time, stride length, step time, step length, step width, walking speed, cadence. Improved gait speed together with a larger symmetry between the paretic and non paretic side due to improved muscle activation patterns at the level of the lower limb are considered to indicate improvement. Since its test-retest reliability is known, gait outcomes will be assessed once at baseline, at 6 wk (mid-study), at the end of the intervention (12 wk) and at the 4 wk follow-up.This is related to Hypothesis 3.1 *(DN will have similar effects than BTX A on gait function, measured with clinical scales and instrumented gait analysis)*.

g- Quality of Life: Health-related Quality of Life will be assessed with the EuroQOL-5D. Responses from the questionnaire will be converted to health-state utility values, where higher values represent improved quality of life. Based on QoL assessments, QALYs will be estimated using the area under the curve (AUC) analysis. It will be evaluated once at baseline, weekly for 12 weeks and at the 4 wk follow-up, as QoL is needed as well as MMAS to calculate cost-effectiveness throughout the study. This is related to Hypothesis 4.1 *(DN will have a similar impact than BTX A on the patients' quality of life)*.

h- Costs and cost-effectiveness: Direct and indirect costs related to treatment, medication and patient care will be assessed by computing the ICER (Incremental cost-effectiveness ratio) in €/QALY. The approach of non-parametric bootstrapping will be adopted to represent the uncertainty surrounding the ICER estimate. It will be calculated at the end of study although the costs will be collected throughout the study. This is related to Hypothesis 4.2 *(the cost-effectiveness of both treatments will be comparable considering that the administration procedure is different due to the different mechanism of action).*

Apart from the aforementioned scientific objectives and outcomes, the project aims to contribute to the improvement of evidence-based practice through a Public and Patient Involvement (PPI) approach, which will also take into account the patient's preferences and expectations (patient-centered approach). For this reason, a Patient Advisory Group will be created with the objective of engaging patients, asking for feedback during the study and contributing to the dissemination of results. Each country will have a group composed of patients and health care professionals that will meet coinciding with the main project´s milestones. There will also be an International Group inviting participants from different countries, who will meet after the national groups have met. The design of this Patient Advisory Group will be discussed at the kick-off meeting (M1). It is expected to have an initial meeting with the user group during the first consortium meeting (M8), where the assessment procedures will be standardized and training for the project staff will be done before the clinical POC starts. The subsequent consortium meetings will include the consultation with the Patient Advisory Group to get feedback throughout the study.

Experimental groups, procedures and interventions:

It is a prospective multicenter feasibility randomized clinical trial comparing DN and BTX A treatment on the relief of spasticity in plantar flexor muscles (gastrocnemius medialis and lateralis, soleus and tibialis posterior). We will use a single-subject multiple-baseline design for the main outcome measures. Each participant will have 3 baseline assessments of the primary measure separated by 1 week to meet the recommended minimum of three baseline points suggested by the What Works ClearingHouse for single case experimental designs (Kratochwill et al., 2010).

Following the baseline period, at week 1, pairs of two participants, stratified by spasticity severity with the MMAS (moderate= grade 1 and severe = grades 2 and 3) will be block-randomized to one of two interventions,

DN treatment or BTX A injection. Block randomization will be repeated in 15 pairs of subjects per site. Patients in the DN group will receive 12 weekly sessions of DN whereas patients in the BTX A group will receive injections of BTX A in the plantar flexors at week 1. The muscles treated by DN or BTX A will be classified into two categories: mandatory muscles (gastrocnemius medial head, gastrocnemius lateral head, soleus and tibialis posterior) and optional muscles (flexor digitorum longus, flexor digitorum brevis, flexor hallucis longus and extensor hallucis longus).

The intervention protocol for BTX A will consist of injecting the mandatory muscles to achieve a total dose of 300 units (Table 1). An additional 100 units may be injected into optional muscles for a total maximal dose of 400 units (Esquenazi et al., 2017).The final protocol will be discussed and agreed with the clinical team in the three countries. The key point will be using the same preparation, as there are currently three leading botulinum neurotoxin type A products available in the Western Hemisphere: onabotulinum toxin-A (ONA) Botox®, abobotulinum toxin-A (ABO), Dysport®, and incobotulinum toxin A (INCO, Xeomin®), and there is an intense debate regarding the comparability of various preparations.

Mandatory Ankle Muscles	Dose	Optional Muscles	Dose
Gastrocnemius medial head	75 U (25 U×3 sites)	Flexor digitorum longus	50 U (25 U×2 sites)
Gastrocnemius lateral head	75 U (25 U×3 sites)	Flexor digitorum brevis	25 U (1 site)
Soleus	75 U (25 U×3 sites)	Flexor hallucis longus	50 U (25 U×2 sites)
Tibialis posterior	75 U (25 U×3 sites)	Extensor hallucis longus	25 U (1 site)

Table 1. Recommended intervention protocol for onabotulinum toxin-A (ONA) Botox®.

Effects of treatment will be evaluated at 3 levels of the International Classification of Functioning (International Classification of Functioning, Disability and Health (ICF)) by blinded evaluators. At the Body Structure and Function level, we will evaluate the effects at the central level using a physiological measure of spasticity (Tonic Stretch Reflex Threshold, TSRT and its velocity sensitivity, mu) in the plantar flexor muscles. At the muscle level, ultrasound imaging and a manual test of perceived resistance to stretch (the MMAS) will be used. At the Activity Level, an assessment of gait activities will be performed with clinical scales (TUG and 10MWT) with sensorized insoles and with instrumented gait analysis. At the Participation Level, quality of life will be measured (EuroQOL-5D) (Chen et al., 2016).

This is a POC study and therefore we do not have data to estimate the sample size. Moreover, one of the objectives of this feasibility study is to have data to allow the estimation of sample size for future clinical trials. We have followed the Recommendations for Planning Pilot Studies in Clinical and Translational Research (Moore et al., 2011) to define the sample size, which will have 90 participants in total, 30 per country. This publication recommends a minimum of 12 participants for pilot studies with the objective to estimate mean values and variability which allows future planning of clinical studies. Therefore,considering a drop-out rate of 20%, 15 patients will be recruited in each group in each country (30 patients per country). This sample size would be valid according to Julious (2005).

Inclusion criteria: 1) patients aged 18-75 years old; 2) having lower limb post-stroke spasticity in ankle plantar flexors (MMAS scores of 1, 2 and 3); 3) having had a first stroke; 4) 3 to 12 months evolution since stroke; 5) no previous DN or BTX A treatment; and 6) ankle PROM ≥ 30°; 7) able to ambulate independently with or without aids.

Exclusion criteria: 1) having other neurological and/or orthopedic conditions that may interfere with the interpretation of data; 2) unable to give informed consent.

We do not expect that the study will be discontinued at a clinical center or a country, as the two treatments have a low rates of adverse events. Although the different clinical sites have good access to patients, if the recruitment rate is lower than expected (i.e., COVID or other reasons), efforts will be made to increase the number of referring clinical centers to increase the recruitment rate. At the patient level, each participant will be able to withdraw from the study at any time, without providing any explanations with the assurance that this will not affect his/her treatment. This information will be included in the informed consent forms.

To minimize the effects of bias, participants will be randomized and evaluators will be blinded, as it is not possible to blind the patients nor the healthcare professionals. Although randomization is guaranteed by the randomization process itself, allocating the participants by pairs will allow the allocation of participants equally to the 2 groups throughout the time frame of the study. This will minimize the potential bias of assigning similar participants to one group at the beginning or at the end of the study, as may occur in studies of small samples. Sampling bias will also be minimized by collecting 3 baseline values for most measures prior to group allocation of the participants. Confounding variables, such as the number of hours of physiotherapy treatment, change in medication, and comorbidities will be monitored during the study and used as control variables when appropriate. Standardization of assessment and treatment procedures amongst countries will be carried out.To decrease the risk of bias related to drop-outs, an intention-to-treat analysis will be performed, and its results compared to the per protocol analysis, to analyze if drop-outs may have affected the final results.

Sex and Gender Considerations: Stroke incidence, prevalence, mortality and outcomes differ between men and women (Ones et al. 2009). During most of the life span, men have a higher incidence of stroke than women. However, women over 85 years old are more likely to have a stroke, leading to excess disability and mortality. Women tend to be treated less aggressively for primary and secondary stroke prevention and receive less treatment in the acute stage. Women have higher rates of depression, worse recovery and are more likely to require assistance after stroke than men, even when controlling for factors such as age and premorbid function. Thus, it is important to account for sex differences in this study. To this end, we will strive to obtain equal numbers of men and women. We will account for sex in primary and secondary outcomes and consider it as a confounding factor in the data analysis.

Gender is not expected to influence the response to spasticity treatment, since spasticity is a pathological state of body function (i.e. muscle reflex activity) not affected by socially constructed identities. However, other socio-cultural factors such as age, time since stroke and number of comorbidities, may influence outcomes. Family care capacity, marital status, degree of illness acceptance, depression and sense of self-efficacy in patients with stroke have been related to poorer treatment outcomes. In our proposal, age, time since stroke and health status are controlled for by the inclusion/exclusion criteria. Socioeconomic status will not hamper participation since travel expenses will be reimbursed equally. We will assess the impact of individual factors of treatment outcomes at the mid-point, end and follow-up timepoints of the study. A better understanding of how sex and gender affect spasticity outcomes will permit more appropriate treatment prescription for women and men.

The recruitment and consent rates will be similar at all 3 study sites. The three participating countries have access to the required sample for this POC study. The current admission rate in the clinics where the consortium partners can recruit patients are: 20 patients/month for Canada, 16 patients /month for Belgium and 24/month for Spain.

- Spain: IIS Aragon (IISA) will recruit from the two biggest hospitals of Zaragoza, Hospital Universitario Miguel Servet and Hospital Clínico Universitario as they are part of IIS Aragon. Moreover, it has an agreement with many other clinical centers and recruitment will be extended to the Hospital “Obispo Polanco” in Teruel, as this is attending rural areas and therefore relevant for the project.

- Belgium: The University of Antwerp (ANT) has several close collaboration agreements with large (rehabilitation) hospitals in the vicinity of Antwerp and Flanders related to this project (Rehabilitation Hospital Revarte and its related campus Hof Ter Schelde, AZ Turnhout). If recruitment from the primary clinical partners is insufficient, alternative existing collaborations can be accessed to mitigate the insufficient inclusion rate at the primary settings (GastHuisZusters Antwerp GZA, Ziekenhuis Netwerk Antwerpen ZNA and AZ Monica). One of the applicants (WS) is also working part-time at the Rehabilitation Hospital Revarte which will increase the feasibility of this project within this hospital.

- Canada: Research will be conducted in the Sensorimotor Control and Rehabilitation Lab of Dr. Levin situated at the Jewish Rehabilitation Hospital, affiliated with McGill University (MGU) and the University of Montreal. Patients seeking treatment for spasticity will be recruited from Physiotherapie Universelle (24 clinics) and Physio Proform for DN and from neurology clinics led by Dr. T. Wein and Dr. M. Haziza at the McGill University Health Center (4 major hospitals in Montreal). Additional clinical collaborators will be included if required to increase the recruitment rate.

C. Statistical analysis

For the statistical analysis, the following analyses will be carried out: 1) Univariate descriptive analysis. Frequency distribution of the qualitative variables will be shown in each category. Quantitative variables will

be tested for normal distribution by means of Kolmogorov-Smirnov test and indicators of central tendency (mean or median) and dispersion (standard deviation or percentiles) will be computed; 2) Analytical statistics. Analysis of equivalence or non-inferiority will be performed by means of contrast hypothesis, comparing proportions of qualitative variables (Chi Square, Fisher Exact test) or comparison of means of quantitative variables (Student t, ANOVA) and bivariate correlations (Pearson's correlation coefficients) when both are quantitative. If the distribution is not adjusted to normalcy, the Mann-Whitney U or Kruskal-Wallis tests will be used. The same tests adjusted for repeated samples will be applied; 3) The analysis will include multivariate regression models that will include the significant variables of the analytical statistics as well as possible confounding variables. A significance level (alpha) of 5% will be used to consider statistical significance.

Statistical power will be calculated to achieve a minimum detectable change of 6.1º in the primary outcome measure (Tonic Stretch Reflex Threshold, TSRT) and 0.05 s for mu (Blanchette et al., 2016). Moreover, it will calculate the precision of the estimations carried out. Methodological advice to design this POC study has been received from SAME, which is a service for methodology and statistics belonging to IACS, which is part of IIS Aragon, the leading partner. Moreover Eva López Hernández, the coordinator of the IIS Aragon Clinical Research Unit and a member of the SCREN (Spanish Clinical Research Network) and ECRIN (European Clinical Research Infrastructure Network), will also be part of the team providing support in the development of the study.

D. Work package structure

Temporal planning: (Figure 6. Gantt Chart)

WP1: Project management and quality control. WP Leader: IISA. Contributors: ANT, MGU.

This WP includes the activities related to project management and reporting including: (1) manage the consortium; (2) manage financial, legal, administrative and technical matters involved in the project; (3) act as the main contact point for the ERA-NET Neuron Secretariat; (4) approve and submit the project deliverables; (5) organize and chair the consortium meetings; (6) supervise and contribute to the communication and reporting strategy; (7) ensure resource sharing and usage as well as overall smooth execution of the project activities; and (8) ensure the flow of information to the partner teams and signal any delay in providing the requested contributions.

T1.1 Project management and reporting (M1-M36). Task Leader*:* IISA. Contributors: ANT and MGU.

- **Conflict resolution and decision-making:** one of the first points to be managed will be the elaboration of a consortium agreement in terms of decision making and conflict resolution.
- **Project meetings:** regular online meetings will be held according to the project needs throughout the project. In order to guarantee the standardization of both assessments and treatments as well as a successful project execution, 5 face-to-face consortium meetings will be held:
  1) Kick-off meeting (M1), which will be organized in **Spain** and will have the objective of defining all the coordination and management procedures, as well as any administrative, financial or legal aspects related to the project. In this meeting, the project details will be revised and agreed upon by the partners and the final version of the consent form will be submitted to each national Ethics Committee **(related to Milestone 1- M6)**;
  2) 1st face-to-face Meeting (M8): organized in Canada: The main objective is to standardize the assessment procedures and training for the project´s staff before the clinical POC starts **(related to Milestone 2, M10);**
  3) 2nd face-to-face Meeting (M18): This meeting, just in the midpoint of the project, will be in **Belgium** and be aimed at reviewing the preliminary results as well as any problems or delays that may have occurred during the project to date, and to apply mitigation actions **(related to Milestone 3, M18);**
  4) 3rd face-to-face Meeting (M28) in Belgium: The objective will be to analyze the project results, and to discuss how to present the results in scientific journals by areas of expertise **(related to Milestone 4- M28);**
  5) Final project Meeting (M36) in Spain: The goal will be to analyze the progress of scientific publications submitted or in preparation, as well as to finalize the administrative tasks to close the project **(Milestone 5- M36).**

T1.2: Quality control and data management (M1-M36). Task Leader: IISA. Contributors: ANT and MGU.

Quality control metrics will be defined to measure the progress of the work being achieved. Each partner will be responsible for assuring the quality of their deliverables and for adopting the most appropriate quality-assurance measures to contribute to the fulfillment of the activity objectives. This task includes activities related to quality control: (1) elaboration of a quality management plan; (2) elaboration of the data management plan; (3) elaboration of progress reports used for quality supervision; (4) quality supervision, identifying deviations from the work plans. IISA will lead the task, will supervise the quality control at the project level, coordinate the quality plan and reporting, and prepare and administer the quality-control tools. The consortium will agree on a specification of the format and mandatory minimum content for the project deliverables. To ensure quality, all deliverables will be reviewed by all the partners

WP2: Clinical studies (M1-M28). WP Leader: MGU. Contributors: IISA and ANT.

T2.1 Design of multicenter clinical study and protocol development (M1-M2): based on the project submitted, minor details will be discussed during the kick-off meeting to ensure that it fits well with all clinical centers enrolling patients in each country. **Task Leader: MGU.** Contributors: IISA and ANT.
T2.2 Local ethics application (M3-M6): We plan to submit ethics applications in M3. Approval may take longer in some countries compared to others but is expected at M6 at the latest. **Task Leader: MGU.** Contributors: IISA and ANT.
T2.3 Evaluator training and standardization of clinical procedures (M6-M9), in coordination with the 1st face-to-face consortium meeting (**M8**), assessment and treatment procedures will be standardized and trained to ensure the homogeneity of both assessments and treatment in the three participating countries. **Task Leader: MGU.** Contributors: IISA and ANT.
T2.4 Implementation and follow-up for the clinical study (M10-M28). Task Leader: IISA. Contributors: ANT and MGU. Physicians applying BTX A injections, clinicians applying DN and clinical evaluators from all centers will meet on a regular basis to ensure standardization of all procedures and to discuss problems that may arise during the implementation of the clinical study.

WP3: Communication and Dissemination (M1-M36). WP Leader: ANT. Contributors: MGU and IISA.

T3.1 Communication of the project´s activities to the general audience through website and social media (M1-M36). Task Leader: ANT. Contributors: IISA and MGU. This task will be equally shared by all partners in their respective official languages but coordinated in a common website and social media managed by ANT.
T3.2 Dissemination of the project´s activities and results for the scientific/professional audience: scientific publications. (M29-M36). Leader: ANT. Contributors: IISA and MGU. This task includes scientific publications for specific audiences (6 publications in open access) and dissemination in Congresses (expected to disseminate in the CIFI - International Congress of Invasive Therapy). This task will be equally shared by all partners, with each of them leading the task related to the deliverable. MGU will lead dissemination related to mechanisms of action of DN and BTX A, ANT will lead the clinical and instrumented gait analysis and IISA will lead the results related to functionality and quality of life, as well as specific results related to secondary analysis of cost-effectiveness and muscle architecture and echotexture.
T3.3 Exploitation (M29-M36). Leader: ANT: exploitation will be discussed amongst partners to analyze if there are results that can be exploited by the whole consortium and/or others that could be exploited by one of the partners, mainly if this comes from their previous knowledge or it is more related to their area of expertise. Intellectual property agreements will be developed if needed. Details of potential exploitation by the consortium as a whole and by each consortium partner is found in section 6.1.

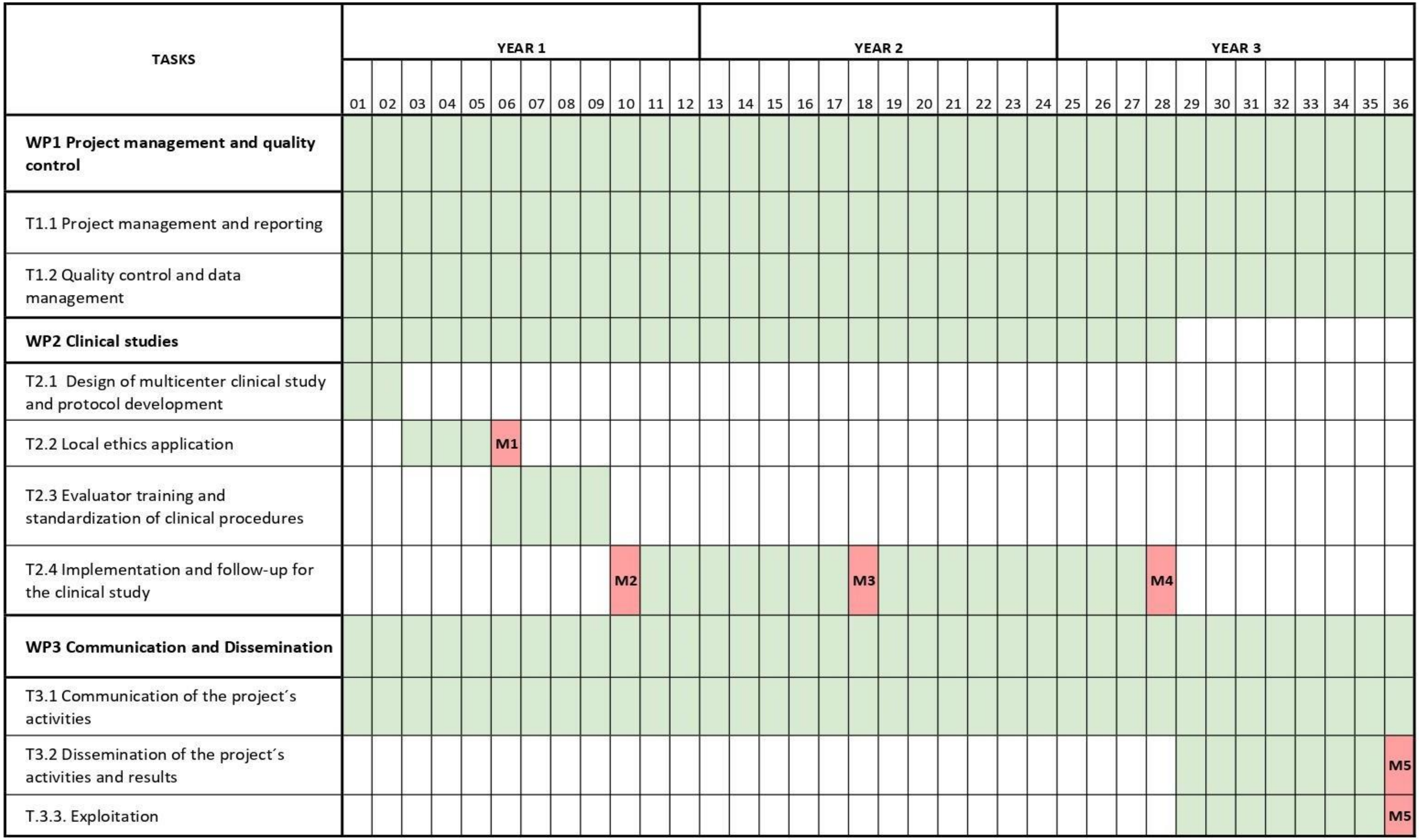

Figure 6. Gantt Chart

Deliverables

D.1 Ethics approval of study protocol (M6)
D.2: Data management plan and data sharing agreements (M6)
D.3 1st Annual scientific progress report (M12): This report will be submitted by the consortium coordinator to the Joint Call Secretariat and will include the project's evolution in the first 12 months. In case of any delays or problems that may have arisen during the project, it will include the mitigation measures that have been adopted.
D.4 Mid-term report (M18): The coordinators will present a progress report during a Midterm symposium. Both the coordinator and the project partners will attend the mid-term symposium.
D.5 2nd Annual scientific progress report (M24): This report will be submitted by the consortium coordinator to the Joint Call Secretariat and will include the project's evolution between month 12 and 24. In case of any delays or problems that may have arisen during the project, it will include the mitigation measures that have been adopted.
D.6 Final project report (M36): This will include the scientific publications submitted and/or published. Expected publications will be the following:

D4.1 Comparative mechanism of action on spasticity between DN and BTX A. **Responsible MGU**. Contributions from IISA and ANT.

D4.2 Comparison between DN and BTX A as a treatment for spasticity, functional mobility, and quality of life in patients with stroke. **Responsible IISA**. Contributions from MGU and ANT.

D.4.3 The impact of BTX A on clinical gait assessment compared to DN in patients with lower limb spasticity. **Responsible: ANT**. Contributions from IISA and MGU.

D.4.4 The Impact of DN on instrumented gait assessment (spatial-temporal parameters and biomechanics) compared to BTX A in patients with lower limb spasticity. **Responsible: ANT.** Contributions from IISA and MGU.

D.4.5 Cost-effectiveness analysis of DN and BTX A. **Responsible IISA.** Contributions from MGU and ANT.

D.4.6 : Comparative ultrasound analysis of muscle architecture and echo intensity between DN and BTX A. **Responsible IISA.** Contributions from MGU and ANT.

Milestone plan (and Months):

Milestone 1 (M6): approval from local ethics committees, data management plan, data sharing agreements
Milestone 2 (M10): start of feasibility study
Milestone 3 (M18): follow-up of feasibility report
Milestone 4 (M28): final results of the feasibility study
Milestone 5 (M36): final project report, including dissemination activities and quality control

References

Al-Boloushi Z, Gómez-Trullén EM, Arian M, Fernández D, Herrero P, Bellosta-López P. (2020). Comparing two dry needling interventions for plantar heel pain: a randomised controlled trial. *BMJ Open*, *10*(8), e038033.

American Stroke Association. (n.d.). Retrieved June 20, 2022, from https://www.stroke.org/en/help-and-support?docID=1941

Béjot Y, Bailly H, Durier J, Giroud M. (2016). Epidemiology of stroke in Europe and trends for the 21st century. *La Presse Médicale*, *45*(12), e391–8. doi: 10.1016/J.LPM.2016.10.003

Blanchette AK, Mullick AA, Moïn-Darbari K, Levin MF. (2016). Tonic stretch reflex threshold as a measure of ankle plantar-flexor spasticity after stroke. *Phys Ther*, *96*(5), 687–95. doi: 10.2522/PTJ.20140243

Blanco-Díaz M, Ruiz-Redondo R, Escobio-Prieto I, De la Fuente-Costa M, Albornoz-Cabello M, Casaña J. (2022). A Systematic review of the effectiveness of dry needling in subacromial syndrome. *Biol*, *11*(2).

Blazevich AJ, Gill ND, Zhou S. (2006). Intra- and intermuscular variation in human quadriceps femoris architecture assessed in vivo. *J Anat*, *209*(3), 289–310. doi: 10.1111/J.1469-7580.2006.00619.X

Bosque M, Margalef R, Carvajal O, Álvarez, DMMS. Dry needling produces mild injuries irrespective to muscle stiffness and tension in ex vivo mice muscles. *Pain Res Management. In Press*.

Boyce D, Wempe H, Campbell C, et al. (2020). Adverse events associated with therapeutic dry needling. *Int J Sports Phys Ther, 15*(1), 103. doi: 10.26603/ijspt20200103

Brav EA, Sigmond H. (1941). The local and regional injection treatment of low back pain and sciatica. *Ann Intern Med, 15* (5), 840. doi.org/10.7326/0003-4819-15-5-840

Calota A, Feldman AG, Levin MF. (2008). Spasticity measurement based on tonic stretch reflex threshold in stroke using a portable device. *Clin Neurophysiol*, *119*(10), 2329–37. doi: 10.1016/J.CLINPH.2008.07.215

Chan CH. (1990). Dantrolene sodium and hepatic injury. *Neurology*, *40*(9), 1427–32.

Chan PP, Si Tou JI, Tse MM, Ng SS. (2017). Reliability and validity of the timed up and go test with a motor task in people with chronic stroke. *APMR*, *98*(11), 2213–20. doi: 10.1016/j.apmr.2017.03.008

Chang KV, Wu WT, Huang KC et al. (2018). Limb muscle quality and quantity in elderly adults with dynapenia but not sarcopenia: An ultrasound imaging study. Exp Ger, 108, 54–61. doi: 10.1016/J.EXGER.2018.03.019

Chen P, Lin KC, Liing RJ. et al. (2016). Validity, responsiveness, and minimal clinically important difference of EQ-5D-5L in stroke patients undergoing rehabilitation. Qual Life Res 25, 1585–1596. doi: 10.1007/s11136-015-1196-z

Cheng DK, Nelson M, Brooks D, Salbach NM. (2019). Validation of stroke-specific protocols for the 10-meter walk test and 6-minute walk test conducted using 15-meter and 30-meter walkways. *27*(4), 251–61.

Dajpratham P, Kuptniratsaikul V, Kovindhaet A et al. (2009). Prevalence and management of poststroke spasticity in Thai stroke patients: multicenter study. *J Med Assoc Thai,* 92(10):1354-60. PD: 19845244

Das TK, Park DM. (1989). Effect of treatment with botulinum toxin on spasticity. *Postgrad Med J*, *65*(762), 208.

De Sire A, Moggio L, Demeco A, et al. (2021). Efficacy of rehabilitative techniques in reducing hemiplegic shoulder pain in stroke: Systematic review and meta-analysis. *Ann Phys Rehabil Med*, *65*(5).

Del-C-Fernández A, C-Martínez P, D-Hoyas et al. (2022).The application of image texture analysis techniques on the effects of dry needling versus placebo in low-back pain patients: pilot-study. *App Sci, 12*(11), 5556.

Domingo A, Mayoral O, Monterde S, Santafé MM. (2013). Neuromuscular damage and repair after dry needling in mice. *Evidence-Based Compl Alternative Med: ECAM*, *2013*. doi: 10.1155/2013/260806

Dorňák T, Justanová M, Konvalinková, et al. (2019). Prevalence and evolution of spasticity in patients suffering from first-ever stroke with carotid origin: a prospective, longitudinal study. *Eur J Neurol*, *26*(6), 880–6.

Esquenazi A, Alfaro A, Ayyoub Z, et al. (2017). OnabotulinumtoxinA for lower limb spasticity: guidance from a delphi panel approach. *PM and R*, *9*(10), 960–8. doi: 10.1016/j.pmrj.2017.02.014

Feigin VL, Stark BA, Johnson CO, et al. (2021). Global, regional, and national burden of stroke and its risk factors, 1990–2019: systematic analysis for Global Burden of Disease Study 2019. *Lancet Neurol*, *20*(10), 795–820.

Fernández-de-Las-Peñas C, Pérez-Bellmunt A, Llurda-Almuzara L, et al. (2021a). Is dry needling effective for the management of spasticity, pain, and motor function in post-stroke patients? systematic review and meta-analysis. *Pain Med*, *22*(1), 131–41. doi: 10.1093/PM/PNAA392

Fernández-Sanchis D, Brandín-De la Cruz N, Jiménez-Sánchez C, Gil-Calvo M., Herrero P, Calvo S. (2022a). Cost-effectiveness of upper extremity dry needling in chronic stroke. *Healthcare*, *10*(1).

Fernández Sanchis D, Cuenca Zaldívar JN, Calvo S, Herrero P, Gómez Barrera M. (2022b). Cost-effectiveness of upper extremity dry needling in the rehabilitation of patients with stroke. *Acupuncture Med*, *40*(2), 160–8.

Foley N, Murie-Fernandez M, Speechley M, et al. (2010). Does the treatment of spastic equinovarus deformity following stroke with botulinum toxin increase gait velocity? A systematic review and meta-analysis. *Eur J Neurol*, *17*(12), 1419–27. doi: 10.1111/J.1468-1331.2010.03084.X

Franceschini M, Iocco M, Molteni F, Santamato A, Smania N. (2014). Management of stroke patients submitted to botulinum toxin type A therapy: a Delphi survey of an Italian expert panel of specialist injectors. *Eur J Phys Rehabil Med, (5):*525-33. PMID: 24963604

Ghannadi S, Shariat A, Ansari NN, et al. (2020). The effect of dry needling on lower limb dysfunction in poststroke survivors. *J Stroke Cerebrovas Dis*, *29*(6):104814.

Ghotbi N, Ansari NN, Naghdi S, Hasson S. (2011). Measurement of lower-limb muscle spasticity: Intrarater reliability of Modified Modified Ashworth Scale. *J Rehab Res Dev, 48*(1), 83–8.

Goldstein EM. (2001). Spasticity management: an overview. *J Child Neurol*, *16*(1), 16–23.

Gupta AD, Chu WH, Howell S, et al. (2018). A systematic review: efficacy of botulinum toxin in walking and quality of life in post-stroke lower limb spasticity. *Syst Rev*, *7*(1), 1. doi: 10.1186/S13643-017-0670-9

Hadi S, Khadijeh O, Hadian M, et al. (2018). The effect of dry needling on spasticity, gait and muscle architecture in patients with chronic stroke: A case series study. *Top Stroke Rehabil*, *25*(5), 326–32.

Hernández Herrero D, Miangolarra Page JC. (2021). Descriptive analysis of the annual cost of treating spasticity with different types of botulinum toxin A. *Neurología,* 18; S2173-5808(21)00177-2.

Herrero Gallego P, Del Moral OM. (2010). A case study looking at the effectiveness of deep dry needling for the management of hypertonia. *J Musc Pain, 15*(2), 55–60. doi: 10.1300/J094v15n02_09.

Hulme A, MacLennan WJ, Ritchie RT, John VA, Shotton PA. (1985). Baclofen in the elderly stroke patient its side-effects and pharmacokinetics. *Eur J Clin Pharmacol, 29*(4), 467–9. doi: 10.1007/BF00613463

International Classification of Functioning, Disability and Health (ICF). (n.d.). Retrieved June 20, 2022, from https://www.who.int/standards/classifications/international-classification-of-functioning-disability-and-health

Jamous A, Kennedy P, Grey N. (1994). Psychological and emotional effects of the use of oral baclofen: a preliminary study. *Paraplegia*, *32*(5), 349–53. doi: 10.1038/SC.1994.59

Julious SA. (2005). Sample size of 12 per group rule of thumb for a pilot study. *Pharmaceut Stat*, *4*(4), 287–91.

Khan I, Ahmad A, Ahmed A, Sadiq S, Asim HM. (2021). Effects of dry needling in lower extremity myofascial trigger points. *J Pakistan Med Assoc*, *71*(11), 2596–603. doi: 10.47391/JPMA.01398

Kratochwill TR, Hitchcock J, Horner RH, et al. (2010). What Works Clearinghouse single-case design technical documentation. Retrieved from http://ies.ed.gov/ncee/wwc/pdf/wwc_scd.pdf.

Lapeyre E, Kuk, JBM, Meijler WJ. (2010). Spasticity: revisiting the role and the individual value of several pharmacological treatments. *NeuroRehabili*, *27*(2), 193–200. doi: 10.3233/NRE-2010-0596

Levin MF, Feldman AG. (1994). The role of stretch reflex threshold regulation in normal and impaired motor control. *Brain Res, 657*(1–2), 23–30. doi: 10.1016/0006-8993(94)90949-0

Levin MF, Selles RW, Verheul MHG, Meijer OG. (2000). Deficits in the coordination of agonist and antagonist muscles in stroke patients: implications for normal motor control. *Brain Res*, *853*(2), 352–69.

Luengo-Fernandez R, Violato M, Candio P, Leal J. (2020). Economic burden of stroke across Europe: A population-based cost analysis. *Eur Stroke J*, *5*(1), 17–25. doi: 10.1177/2396987319883160

Martínez-Payá JJ, Ríos-Díaz J, Del Baño-Aledo ME, et al. (2017). Quantitative muscle ultrasonography using textural analysis in amyotrophic lateral sclerosis. *Ultrasonic Imaging*, *39*(6), 357–68.

Mathevon L, Michel F, Decavel P, Fernandez B, Parratte B, Calmels P. (2015). Muscle structure and stiffness assessment after botulinum toxin type A injection. A systematic review. *APRM 58*(6), 343–50.

Mathevon L, Michel F, Aubry S, et al. (2017). Two-dimensional and shear wave elastography ultrasound: A reliable method to analyse spastic muscles? *Muscle & Nerve*, *57*(2), 222–228. doi: 10.1002/MUS.25716

May S, Locke S, Kingsley, M. (2021). Reliability of ultrasonographic measurement of muscle architecture of the gastrocnemius medialis and gastrocnemius lateralis. *PLOS ONE*, *16*(9), e0258014.

McGuire JR. (2015). Effective use of chemodenervation and chemical neurolysis in the management of poststroke spasticity. Top Stroke Rehabil, 8(1), 47–55. doi: 10.1310/CYP4-BPXC-CG8M-XCA3

Moore CG, Carter RE, Nietert PJ. Stewart PW. (2011). Recommendations for planning pilot studies in clinical and translational research. Clinical and Translational Science, 4(5), 332–7. doi: 10.1111/j.1752-8062.2011.00347.x

Mullick AA, Musampa NK, Feldman AG, Levin MF. (2013). Stretch reflex spatial threshold measure discriminates between spasticity and rigidity. *Clin Neurophysiol*, *124*(4), 740–51. doi: 10.1016/J.CLINPH.2012.10.008

Musampa NK, Mathieu PA, Levin MF. (2007). Relationship between stretch reflex thresholds and voluntary arm muscle activation in patients with spasticity. *Exp Brain Res*, *181*(4), 579–93. doi:10.1007/S00221-007-0956-6

Nichols TR, Steeves JD. (1986). Resetting of resultant stiffness in ankle flexor and extensor muscles in the decerebrate cat. *Exp Brain Res*, *62*(2), 401–10. doi: 10.1007/BF00238859

Oh HM, Park GY, Choi YM, Koo HJ, Jang Y, Im S. (2018). The Effects of botulinum toxin injections on plantar flexor spasticity in different phases after stroke: a secondary analysis from a double-blind, randomized trial. *PM & R : J Injury Func Rehabil*, *10*(8), 789–97. doi: 10.1016/J.PMRJ.2018.02.011

Oneş K, Yalçinkaya EY, Toklu BC, Cağlar N. (2009). Effects of age, gender, and cognitive, functional and motor status on functional outcomes of stroke rehabilitation. *NeuroRehabil*, *25*(4), 241–9.

Podsiadlo D, Richardson S. (1991). The timed “Up & Go”: a test of basic functional mobility for frail elderly persons. *J Am Ger Soc*, *39*(2), 142–8. doi: 10.1111/J.1532-5415.1991.TB01616.X

Ríos-Díaz J, del Baño-Aledo ME, Tembl-Ferrairó JI, et al. (2019). Quantitative neuromuscular ultrasound analysis as biomarkers in amyotrophic lateral sclerosis. *Eur Radiology*, *29*(8), 4266–75.

Rodríguez-Huguet M, Vinolo-Gil MJ, Góngora-Rodríguez J. (2022). Dry needling in physical therapy treatment

of chronic neck pain: systematic review. *J Clin Med*, *11*(9): 2370. doi: 10.3390/JCM11092370

Salom-Moreno J, Sánchez-Mila Z, Ortega-Santiago R, et al. (2014). Changes in spasticity, widespread pressure pain sensitivity, and baropodometry after the application of dry needling in patients who have had a stroke: a randomized controlled trial. *J Manip Physiol Ther, 37*(8), 569–79. doi: 10.1016/J.JMPT.2014.06.003

Sánchez-Mila Z, Salom-Moreno J, Fernández-de-las-Peñas C. (2018). Effects of dry needling on post-stroke spasticity, motor function and stability limits: a randomised clinical trial. *Acupuncture Med, 36*(6), 358–66.

Schindelin J, Arganda-Carreras I, Frise E, et al. (2012). Fiji - an Open Source platform for biological image analysis. *Nature Methods*, *9*(7), 676–82. doi: 10.1038/NMETH.2019

Schroeder AS, Ertl-Wagner B, Britsch S, et al. (2009). Muscle biopsy substantiates long-term MRI alterations one year after a single dose of botulinum toxin injected into the lateral gastrocnemius muscle of healthy volunteers. *Movement Disord*, *24*(10), 1494–1503. doi: 10.1002/MDS.22661

Simon O, Yelnik AP (2010) Managing spasticity with drugs. *Eur J Phys Rehab Med,* 46(3):401-10. P: 20927006

Soyuer F, Öztürk A. (2007). The effect of spasticity, sense and walking aids in falls of people after chronic stroke. *Disabil Rehab*, *29*(9), 679–87. doi: 10.1080/09638280600925860

Subramanian SK, Feldman AG, Levin MF. (2018). Spasticity may obscure motor learning ability after stroke. *J Neurophysiol*, *119*(1), 5–20. doi: 10.1152/JN.00362.2017

Thompson AJ, Jarrett L, Lockley L, Marsden J, Stevenson VL. (2005). Clinical management of spasticity. *J Neurol Neurosurg Psych*, *76*(4), 459–63. doi: 10.1136/JNNP.2004.035972

Tok F, Özçakar L, Safaz I, Alaca R. (2011). Effects of botulinum toxin-A on the muscle architecture of stroke patients: An ultrasonographic study. *J Rehabil Med*, *43*(11), 1016–19. doi: 10.2340/16501977-0876

Turpin NA, Feldman AG, Levin MF. (2017). Stretch-reflex threshold modulation during active elbow movements in post-stroke survivors with spasticity. *Clin Neurophysiol, 128*(10), 1891–7.

Varvarousis DN, Martzivanou C, Dimopoulos D, Dimakopoulos G, Vasileiadis GI, Ploumis A. (2021). The effectiveness of botulinum toxin on spasticity and gait of hemiplegic patients after stroke: A systematic review and meta-analysis. *Toxicon*, *203*, 74–84. doi: 10.1016/J.TOXICON.2021.09.020

Wafa HA, Wolfe CDA, Emmett E, Roth GA, Johnson CO, Wang Y. (2020). Burden of stroke in europe: thirty-year projections of incidence, prevalence, deaths, and disability-adjusted life years. *Stroke*, *51*(8), 2418–27.

Yang KC, Liao YY, Chang KV, Huang KC, Han DS. (2020). The quantitative skeletal muscle ultrasonography in elderly with dynapenia but not sarcopenia using texture analysis. *Diagnostics, 10*(6), 400.

Yavuzer G, Öken Ö, Elhan A, Stam HJ. (2008). Repeatability of lower limb three-dimensional kinematics in patients with stroke. *Gait & Posture*, *27*(1), 31–5. doi: 10.1016/J.GAITPOST.2006.12.016

E. Changes between pre- and full proposal

Reviewer comments	Minor changes
The topic is relatively narrow and the translational aspects are limited	In terms of applicability to clinical practice, the infiltration of BTX A usually requires administration by a medical doctor, in contrast with DN, which is performed mainly by physiotherapists. If DN can demonstrate similar effects as BTX A with at least similar costs, it would have a significant impact in clinical practice for several reasons: 1) the safety of DN would be greater due to the type of needle and the avoidance of infiltrating pharmacological substances; 2) it would increase the accessibility of treatments in rural areas, where in some cases there is no presence of medical specialists to provide BTX A infiltration and requires that patients visit the medical specialist in the specialty centers. This could help not only increase accessibility to the treatment but also to decrease costs associated with patient transportation and the inconveniences for the patients themselves or their families (e.g., days of work lost).<br><br>Moreover, in terms of translation to clinical practice, it is important to note that although the POC study is directed to post-stroke spasticity, spasticity is a common characteristic of many other neurological conditions, so that any advance in the understanding of the mechanisms of action of DN in stroke may be translated to other neurological conditions such as Parkinson Disease, Multiple Sclerosis, Spinal Cord Injury or Traumatic Brain Injury, amongst others, where anecdotal cases have been reported about the benefits of dry needling on spasticity.
The DN technique requires considerable time for the treatment including very many visits to the person performing the intervention.	It is true that although only 1 session of BTX A infiltration is necessary, it is expected that more than one DN session (to be studied) will be necessary to have similar effects. However in this POC study we aim to investigate the mechanism of action of both DN and BTX A and understand the time course of their effects.<br><br>Also related to the previous comment, although DN may involve more visits, these visits may involve fewer inconveniences to patients and their families, as well as fewer costs (i.e transportation, work leave, etc), specially in the case of patients living in rural areas or far from the medical specialty centers.<br><br>Moreover, it is important to highlight that patients usually attend physiotherapy, so that DN could be integrated to already scheduled sessions and would not involve additional time for the patient/families. This could be better analyzed if the mechanisms of action are better understood although this is beyond this POC study.
It is uncertain to what extent the TSRT test will contribute to the understanding of the DN technique.	The clinical studies carried out to date have only measured the effects of DN on spasticity with clinical scales and therefore, the mechanism of action is still unknown. Comparative studies between DN and BTX A are necessary regarding the mechanism of action to understand how they work, as well as the magnitude and duration of effects at the central level. It is expected that the TSRT will allow us to understand the mechanisms involved in DN and BTX A.<br><br>The TSRT and velocity sensitivity measures assess the net alpha motoneuron excitability at the spinal level due to changes in descending and peripheral afferent influences. We expect that a decrease in spasticity will be associated with a higher angular value of the TSRT and a lower sensitivity to velocity. We will compare changes at the central level due to the two techniques to gain insight into their respective mechanisms of action.

The project proposal seems feasible and is well thought out in terms of study protocol, finances, allocation of tasks, and timeline. There is no risk analysis added so.	No project is free of unexpected events that may alter the planned timeline. Because of this, we have included several mitigation actions for potential events that may delay or impact the project:<br><br>It is not expected to have any events that will result in discontinuation of the study at a clinical center or a country, as the two treatments have a low rate of adverse events. Although the different clinical sites have good access to patients, the recruitment rate could be lower than expected (i.e COVID or other reasons). If this is the case, each country will contact other clinical centers to increase the recruitment rate.<br><br>To minimize the effects of bias, the study will be randomized and blinded for evaluators, as it is not possible to blind the patients nor the healthcare professionals. We will avoid bias by allocating patients to groups in pairs, as well as by collecting repeated baseline measurements when indicated for each participant.<br><br>Differential confounding variables will be controlled during the study as mitigation measures (number of hours of physiotherapy treatment, change in medication or other comorbidities, etc).<br><br>Standardization of assessment and treatment procedures amongst countries will be carried out during the consortium meetings. All clinical evaluators at each site will undergo harmonized intensive training on how to apply techniques and measures.
The addition of a patient-centered perspective is of benefit for the long-term clinical application.	Considering a patient-centered approach, there are no alternatives to the BTX A infiltration treatment in terms of non-pharmacological treatments, which should be also taken into account as many patients would potentially prefer a non-pharmacological treatment.<br><br>According to the existing data on safety and adverse effects, DN is expected to be safer than BTX A due to the type of needle used and the lack of injection of pharmacological agents. Moreover, DN has not shown any adverse effects derived from repeated use as it is the case of BTX A, which in the latter case, may lead to negative long-term effects.<br><br>This POC study is expected to improve the understanding of the mechanism of action of both DN and BTX A at central, functional and muscle levels. This should help both health professionals and the health system to know when to offer DN and if DN in some cases could be used as a complement and/or alternative to BTX A for post-stroke spasticity.<br><br>A better understanding of the mechanisms of action, duration of effects and other characteristics of DN and BTX A, will contribute to providing better information to patients, which should lead them to making more informed decisions and therefore contributing to better patient-centered care.<br><br>Moreover, as it has been mentioned, accessibility to spasticity treatment by the availability of DN can be better for patients, especially in rural areas, which may also contribute to better long-term effects, decreasing the impact of stroke not only in patients but also in their families.
Scientific quality is good but the experimental design and data analysis may be improved.	We have simplified and clarified the methodology. On the one hand, the methodology will consist of 1 to 3 baseline assessments followed by 12 weeks of DN treatment with multiple assessments. Moreover, we have included an intention to treat analysis to analyze the potential impact that drop-outs may have in the results, considering the reduced sample size.

	We have also specified the time points for the measurement of each outcome.
There are some conflicting data in the literature regarding its effects (although dry needling seems to have a positive effect on lower limb spasticity in stroke patients, it may have a negative effect on upper limb spasticity) and we do need to understand the mechanisms behind. Therefore, further high-quality studies are needed to confirm or refute the effect of dry needling and this study may be an important proof of concept for a subsequent well designed clinical trial.	We agree that the results are controversial, which is possibly due to the heterogeneity of the interventions carried out. This is exactly why a better understanding of the mechanism of action of both DN and BTX A would help to design better clinical studies.
Establishing one primary objective, with a specific assessment tool, would probably be a more adequate strategy than proposing several measures (including observer dependent techniques), such as ultrasound, TSRT, MMAS and gait measurements.	Following the reviewer's suggestion, we have established only one primary outcome, which is most related to the mechanism of action at a central level. Therefore the **primary objective** is to determine the mechanisms of action of DN and BTX A infiltration treatments on lower limb post-stroke spasticity at central (spinal) levels. ***Primary hypothesis:*** *DN and BTX A infiltration treatments will decrease spasticity by decreasing stretch reflex excitability (measured by an increase in the Tonic Stretch Reflex Threshold (TSRT) angle and a decrease in velocity sensitivity (mu)).*<br><br>To address this objective, we will use a specific assessment tool (TSRT) that has shown to be reliable and valid to measure changes at a central level.
Objectives are relevant although neither of the techniques is novel.	In the short proposal we could not explain some of the novelties of this POC study. The use of the TSRT test will be innovative in this area, as most of the clinical studies have only used clinical scales to measure change and have not addressed the mechanism of action.<br><br>Moreover, at a muscle level, we plan to introduce a combination of both morphometric and echo-textural measures, which have not yet been applied to this specific area.

Apart from the changes related to the reviewers' comments, the changes we have made are the following:

- Small changes in the months that consortium meetings will take place, as we have adjusted them to the main milestones.
- In WP3, we have differentiated between communication and dissemination activities, and we have included exploitation.
- Deliverables have been also modified to be more consistent with the call.
- The Canadian budget has been modified according to its national agency's indications. FRQS asked to rearrange the items so as not to go over $75000 per year and not to go above $225,000 in total over the 3 years. Differences also occurred due to changes in the exchange rate.
- We noticed a mistake in Belgium budget (2500€ were not added), so we decided to readjust the traveling expenses to avoid modifications in the Belgium budget.

3. Data Management Plan -DMP

All quantitative and qualitative data on individual participants of the evaluation study will be entered and stored in one shared database. We will use a specific web based platform (e.g. Hefora or Castor) for collecting and analyzing data. The software allows the setup of a private and secure website for data collection and users are able to submit, view and send information. The software offers extensive respondent management; composing tracks for questionnaires and forms; data overviews per patient, track, questionnaire, round, etc.; multi-center functionality; user-friendly interfaces; elaborate rights management; and extensive security and logging possibilities. Procedures for data cleaning, data analysis and the reporting of results will be established in a data management plan.

All data and outputs generated and managed within the project will be managed in line with the FAIR principles (Findable, Accessible, Interoperable, Reusable). A comprehensive description of data management will be included in the Data Management Plan (DMP), describing the data management life cycle for all data sets that will be collected, processed, or generated within this project. The first version of the DMP will be delivered in month 6 (D2) and, as it is a living document, the final version will be delivered at the end of the project.

The research data will be anonymized and made accessible for verification and re-use. All individuals providing data to the project will be asked to give informed consent for the sharing of data after the completion of the project. Based on GDPR, data can be shared only after careful assessment if the aims of the individual or organization who is requesting the data comply with participants' consent as provided.

STROKE-POC will take into account the European infrastructure EBRAINS for data management and tools, and the scientific data generated within the project will be stored at the "ELIXIR Core Data Resources" or any other repository certified by ELIXIR.

IISA will be in charge of these tasks with the support and approval of the rest of the partners

4. Justification of requested budget for each partner

Spain:

- Personnel: it is expected to contract a researcher (MSc) for 0,5 FTE (18 PM) and a Project Manager for 0,5 FTE (18 PM).
- Consumables: Sanitary materials (needles, nitrile gloves, cotton or gauze, alcohol spray for skin asepsis, container for needles) as well as electrodes for evaluation.
- Equipment: Different equipment will be purchased for evaluation: 1) sensorized insoles, to assess gait out from the lab setting; 2) A portable system for gait analysis based on inertial IMUS to perform analysis directly in the clinical centers, as IISA doesn't have a gait lab or gait analysis at the clinical setting; 3) The Montreal Spasticity Measure, which includes a laptop, the Procomp 5 (device used to collect the EMG data) and a goniometer and 4) portable ultrasound device.
- Travel: Attendance at ERA-NET midterm symposium and face-to-face consortium meetings (2 in Belgium and 1 in Canada).
- Other direct costs: External service consisting of API development to integrate data from the sensorized insoles as well as technical support for raw data processing, 3 open access publications (APCs) and insurance for the clinical trial.
- Overheads: Administrative overheads. 21% of total direct cost

Canada:

- Personnel: Salary is requested for 0,155 FTE for site coordination, Physiotherapist- DN Rx (12 treatments x 15 patients; 360 treatments x 1/2 hour), Physiotherapist blinded evaluator (30 patients x 16 assessments x 2 hours) and a PhD trainee.
- Consumables: Transportation and patient costs, sanitary materials (needles, nitrile gloves, cotton or gauze, alcohol spray for skin asepsis, container for needles) for the dry needling technique, electrodes for evaluation and Botox for the BTX A technique.
- Equipment: The Montreal Spasticity Measure, which includes the Procomp 5 (device used to collect the EMG data), software license and a goniometer. The sensorized insoles to assess gait out from the lab setting will be covered by other sources*.
- Travel: Attendance at ERA-NET midterm symposium and face-to-face consortium meetings (2 in Spain and 2 in Belgium)
- Other direct costs: Technical support for sensorized insoles, raw data processing and 1 open access publication (APCs). This will be covered by other sources*.
- Overheads: none

  * '*Other sources*' refers to funds held by Mindy F. Levin, Canada coordinator, associated with her Distinguished James McGill Professorship ($15,000 per year).

Belgium:

- Personnel: Salary is requested for 0,2 FTE for site coordination, 1 FTE for Physiotherapist-DN Rx PhD and 0,5 FTE for Physiotherapist blinded evaluator.
- Consumables: Transportation and patient costs, sanitary materials (needles, nitrile gloves, cotton or gauze, alcohol spray for skin asepsis, container for needles) for the dry needling technique, electrodes for evaluation and Botox for the BTX A technique.
- Equipment: Different equipment will be purchased for evaluation: 1) sensorized insoles, to assess gait out from the lab setting and 2) The Montreal Spasticity Measure, which includes a laptop, the Procomp 5 and a goniometer.
- Travel: Attendance at ERA-NET midterm symposium and face-to-face consortium meetings (2 in Spain and 1 in Canada)
- Other direct costs: Technical support for sensorized insoles and raw data processing and 2 open access publications (APCs).
- Overheads: Administrative overheads. 17% of total direct cost.

5. Added value of the proposed collaboration

- The research team from Spain (IIS Aragon) includes two large hospitals (Hospital Clínico Universitario y Hospital Miguel Servet) and the University of Zaragoza. The team, directed by Dr. Herrero has extensive experience in the application of both BTX A infiltration and DN, as well as using diagnostic ultrasound for muscle evaluation.They will be responsible for training clinicians and evaluators in all the participating centers to ensure that all treatments and evaluations are performed in the same way. The criteria to infiltrate BTX A or apply DN will be standardized by the participating centers. Moreover, the research team also has experience conducting cost-effectiveness studies (Fernández Sanchis et al., 2022),(Fernández-Sanchis et al., 2022). Eva López Hernández, the coordinator of the IIS Aragon Clinical Research Unit and a member of the SCREN (Spanish Clinical Research Network) and ECRIN (European Clinical Research Infrastructure Network), will also be part of the team providing support in the development of the study. Moreover, SAME has participated in the design of methodology and statistics and will provide advice and supervision during the study.

- Dr. Levin and her group (Canada) have developed an innovative system to measure spasticity based on the objective quantification of reflex excitability at the spinal level. Her previous studies (Mullick et al., 2013) have shown that TSRT and μ differentiate between stroke-related spasticity and Parkinsonian rigidity, evaluate the influence of heteronymous muscle activity on spasticity zones (Musampa et al., 2007), and identify ranges in which movements can/cannot be controlled (Subramanian et al., 2018). The ability to modulate TSRTs and μ during voluntary movement has been related to levels of functional impairment (Turpin et al., 2017). This suggests that these measures may predict the eventual level of sensorimotor recovery as well as track spasticity-relieving treatment effects, the objective of this study. Originally measured using a torque motor and motion sensors, the measurement method was implemented in a portable device (Calota et al., 2008) called the Montreal Spasticity Measure (MSM). All centers will have the same equipment and in-person as well as remote training of evaluators on how to use the MSM and analyze the TSRT and μ data will be done.

- The team of Prof Saeys includes two settings, more specifically the Rehabilitation Hospital Revarte and the University of Antwerp (Belgium). The expertise of the University of Antwerp (ANT) is in trunk, balance, and gait control (assessment and treatment) in stroke patients. ANT has also extensive expertise in gait (movement) analyses and will provide their expertise to implement a reliable and comprehensive clinical and instrumented gait analysis that can be used in the clinical facilities, so that all researchers standardize the gait measurements. In the Rehabilitation Hospital Revarte, patients are going to be recruited and BTX A will be applied by the affiliated medical physicians. The hospital has extensive expertise in the treatment of neurological patients with spasticity and gait and balance disorders.

6. Possible exploitation of expected project results and potential health and clinical impact

6.1 Exploitation of expected project results

As a consortium it will be analyzed if any of the results can be exploited by the whole consortium, signing the agreements to perform this exploitation.

Spain (IISA): researchers of IIS Aragon have a long trajectory in transferring the know-how acquired during research through training services. This was the case of the application of dry needling for neurological patients, which led to the development of a specific methodology to assess and to apply the DN technique, registered as DNHS. Since then, the research team has provided training courses and seminars worldwide. Other potential exploitation could be related to the creation of an algorithm integrated into the ultrasound device software (i.e software licenses) that would allow the clinician to decide in real time the dosage optimal that has to be used based on the histogram analysis of the region of interest (ROI). Moreover, the involvement of basic and clinical researchers at IISA makes the translation of the results into the clinical practices an easy and fluid process. IISA is a member of the EATRIS Research Infrastructure and belongs to its Spanish-Portugal node. This contact will facilitate the translation of the results of the project for further exploitation.

Canada (MGU): The Montreal Spasticity Measure (MSM) was developed by the team in Canada and has since been used to analyze spasticity in adults with stroke, Parkinson Disease and spinal cord injury as well as in children with cerebral palsy in several countries. These include, the University of Chicago at Urbana and Cleveland FES Centre (Case Western Reserve University) in the USA, the University of Florence (Italy), Manipal Academy of Higher Education (India), and Loewenstein Rehabilitation Hospital and Soroka Hospital Beersheva (Israel). The technique of measurement of TSRT and mu has been adopted by the rehabilitation community (over 30 publications) as an alternative to spasticity measurement using clinical scales. Results of the current project will provide new information about whether and to what extent spasticity management techniques at the muscle level may lead to lasting changes in muscle morphology and in reflex excitability at the spinal level. This information will help in clinical-decision making for physicians prescribing these techniques for spasticity management and provide scientific evidence of their effectiveness for decisions about when reinjection or additional treatment sessions may be beneficial to the patient.

Belgium (ANT): Researchers of the research group MOVANT (Movement Antwerp) of the department Rehabilitation Sciences and Physiotherapy have extensive experience in balance and gait disorders. A close collaboration exists between clinical settings, the university of Antwerp and industrial companies. One of the co-applicants (WS) is part time working in a rehabilitation hospital Revarte besides his activities at Antwerp. This means that research can immediately be transferred into clinical practice. Implementing new standards to improve the well-being of patients while decreasing load on the healthcare system and workers is of high importance to our research group. The M²OCEAN gait analysis lab is a reference center for gait, posture and balance research in Flanders and has extensive collaboration agreements with other labs in Europe in transferring the knowledge acquired during research towards the clinical setting through training services. In Belgium, the use of dry needling is widespread among Belgian physiotherapists. Therefore, validation of the Dry Needling technique for spasticity and hypertonia would be quickly noticed by physiotherapists who master the 'conventional' dry needling in both research and clinical practice settings. Moreover, due to a national committee that sensitizes and promotes quality within physiotherapy, new and innovative techniques and treatments would be promoted when any of the mentioned institutions would implement the results in their theoretical background of their courses.

6.2 Potential health and clinical impact

The main potential health and clinical impact from this project is to offer a new treatment to patients with post-stroke spasticity that can be safer, more cost-effective and accessible. Although this will have to be tested during the study, it is expected that DN is safer than BTX A due to the type of needles used and because it does not inject any pharmacological substance. Moreover, the cost of BTX A is high, and if DN can be included in the standard rehabilitation programmes for post-stroke spasticity, this may represent a significant saving. Besides, this would help to make this treatment more accessible if it can be provided by physiotherapists who work in rural areas, which is not the case for medical specialists who work only in the specialty centers, which involves patient transportation and its associated costs and inconvenience for both patients and their families.

7. Brief CVs for each participating group leader with a list of up to five relevant publications within the last five years demonstrating the competence to carry out the project, description of patents and ongoing projects of each participating group related to the present topic, indicating funding sources and possible overlaps with proposal

Electronic proposal submission is mandatory. It is strongly recommended to meet the deadline and observe the format of the proposal structure (DIN-A4; font: Arial, 10pt; page limit). Do not add any additional attachments. All items (such as figures, tables, list of references) have to be included in the work plan (16 pages max., excl. Annex I). Proposals not meeting the formal criteria will be rejected.

Curriculum Vitae: Herrero, P.

Positions:

Clinical Physiotherapist, Aragon Government (2002-2009) Associate
Professor, Universidad Pública de Navarra (2008-2009)
Lecturer, San Jorge University (2009-2020). Vice-Dean of Physiotherapy Degree from 2009 to 2016.Full
Professor, University of Zaragoza (2020 to present)
Head of iHealthy Research Group (2020 to present)
Founder and President of AIDIMO (Association for Research in Motor Handicap), from 2006 topresent

Education/Training

INSTITUTION AND LOCATION	DEGREE	COMPLETION DATE	FIELD OF STUDY
UNIVERSITY OF ZARAGOZA	B.Sc	2001	Physiotherapy
UNIVERSITY OF ZARAGOZA	M.Sc	2009	Rehabilitation
UNIVERSITY OF ZARAGOZA	Ph.D	2012	Physiotherapy

Honors (selected)

Awarded for the best Merging Innovation (patented 3TOOL) in Tercer Milenio Awards

Five relevant publications within the last five years (* indicates trainee, # indicates correspondingauthor):

1. Fernández-Sanchis*, D.; Brandín-de la Cruz, N.; Jiménez-Sánchez, C.; Gil-Calvo, M.; **Herrero, P#**.; Calvo, S. Cost-Effectiveness of Upper Extremity Dry Needling in Chronic Stroke. Healthcare 2022, 10, 160. https://doi.org/10.3390/healthcare10010160
2. Brandín-de la Cruz N*, Calvo S, Rodríguez-Blanco C, **Herrero, P#**, Bravo-Esteban E. Effects of dry needlingon gait and muscle tone in Parkinson's disease: a randomized clinical trial. Acupunct Med. 2022 Feb;40(1):3- 12. https://10.1177/09645284211039232
3. Calvo S*, Brandín-de la Cruz N*, Jiménez-Sánchez C, Bravo-Esteban E, **Herrero, P#**. Effects of dry needlingon function, hypertonia and quality of life in chronic stroke: a randomized clinical trial. Acupunct Med. 2021 Dec 13:9645284211056347. doi: 10.1177/09645284211056347. Epub ahead of print. PMID: 34894776.
4. Fernández Sanchis D*, Cuenca Zaldívar JN*, Calvo S*, **Herrero, P#**, Gómez Barrera M. Cost-effectiveness of upper extremity dry needling in the rehabilitation of patients with stroke. Acupuncture in Medicine. December 2021. https://10.1177/09645284211055750
5. Cuenca Zaldívar JN*, Calvo S*, Bravo-Esteban E, Oliva Ruiz P, Santi-Cano MJ, **Herrero, P#**. Effectivenessof dry needling for upper extremity spasticity, quality of life and function in subacute phase stroke patients. Acupuncture in Medicine. August 2020. JCR Q2. Category: Integrative and Complementary Medicine (position 17/29) FI: 2.267. https://10.1177/0964528420947426

Patents

Postural evaluation device with goniometer and inclinometer. Patent for Spain. Reference ES 1510.71. 15/4/2010.
Expired
Adapted tricycle for cerebral palsy children. Patent for Spain, Reference ES1073280. 18/10/2010. **Expired.** Device for the application of therapeutic actions on the body. Patent for Spain MU0000091/2014. Applicationmade on 27/02/2014. Holder: University of Zaragoza. **In exploitation by Fisio Consultores SL**

Relevant funding – Grants applied for:

Deepening knowledge and improving the Quality of Life in Parkinson Disease through Smart Insoles. Ref. PID2020-116011RB-C22. Period 2021-2024. National Spanish Research Agency. There is no overlap with thepresent proposal.
Development of a tele-physiotherapy tool for the early diagnosis of chronic non-specific low back pain. Ref. LMP97_21. Period 2021-2023. There is no overlap with the present proposal.

Curriculum Vitae: Levin, Mindy F.

Positions:

Full Professor (since 2009), School of Physical and Occupational Therapy, Faculty of Medicine and Health Sciences, McGill University, Montreal, Quebec, Canada.

Director (since 2004), Sensorimotor Control and Rehabilitation Lab, Jewish Rehabilitation Hospital, Centre for Interdisciplinary Research in Rehabilitation, Montreal, Quebec, Canada.

Education/Training

INSTITUTION AND LOCATION	DEGREE	Completion Date	FIELD OF STUDY
McGill University, Montreal, Quebec	B.Sc.	1976	Physical Therapy
University of Montreal, Montreal, Quebec.	M.Sc.	1985	Clinical Sciences
McGill University, Montreal, Quebec	Ph.D.	1990	Physiology
University of Montreal, Montreal, Quebec	Post-doc	1992	Neurological Science

Honors (selected)

1992-2004	Chercheur-boursier Fonds de recherche en Santé du Québec (FRSQ)2005-
2019	Canada Research Chair Tier 1: Motor Recovery and Rehabilitation
2010	Enid Graham Memorial Lecture of the Canadian Physiotherapy Association
2012	American Society of Neurorehabilitation Education and Research Foundation Award2016
	Jonas Salk Lifetime Achievement Award, March of Dimes Canada
2018-present	Fellow of the Canadian Academy of Health Sciences 2019-
present	Distinguished James McGill Professor, McGill University

Five relevant publications within the last five years (* indicates trainee):

1. Turpin N,* Feldman A.G., **Levin M.F.** Stretch-reflex threshold modulation during active elbow movements inpost-stroke survivors with spasticity. *Clinical Neurophysiology,* 2017; 128(10):1891-1897. doi: 10.1016/j.clinph.2017.07.411.
2. **Levin M.F.**, Solomon J., Shah A.*, Blanchette A.K.*, Feldman A.G. Activation of elbow extensors during passive stretch of flexors in patients with post-stroke spasticity. *Clinical Neurophysiology,* 2018;129:2065-2074. doi.org/10.1016/j.clinph.2018.07.007
3. Davidowitz I., Parmet Y., Frenkel-Toledo S., Baniña M.C.,* Soroker N., Solomon J.M., Liebermann D.G., **Levin M.F.,** Berman S. Relationship between spasticity and upper limb movement disorders in patients with stroke using stochastic spatiotemporal modeling. *Neurorehabilitation and Neural Repair,* 2019;33(2):141–152.doi: 10.1177/1545968319826050
4. Piscitelli D.,* Turpin N.A.,* Subramanian1 S.K., Feldman A.G., **Levin M.F.** Deficits in corticospinal control of stretch reflex thresholds in stroke: implications for motor impairment. *Clinical Neurophysiology*, 2020:131:2067-2078. doi: 10.1016/j.clinph.2020.05.030
5. Frenkel-Toledo S.,* Solomon J.S., Baniña M.C.,* Berman B., Soroker N., Liebermann D.G., **Levin M.F.** Tonicstretch reflex threshold as a measure of spasticity after stroke: Reliability, minimal detectable change and responsiveness. *Clinical Neurophysiology*. 2021;132(6):1226-1233. doi: 10.1016/j.clinph.2021.02.390.

Patents

United States Patent No: US 9,265,451 B2, Feb. 23, 2016. “Method and Apparatus for Determining Spasticity” ***expired.***

Relevant funding – Grants applied for:

Sept 2021 — Heart and Stroke Foundation of Canada: G-22-0032010
Improving diagnosis and prognosis of spasticity in post-stroke patients.

The goal of this study is to predict which people who have suffered a recent stroke will develop spasticity in themuscles of the ankle, an area commonly affected by spasticity. Using serial testing over 12 weeks and imaging, we will determine when spasticity occurs using a new physiological test of stretch reflex excitability combined with common clinical measures. There is no overlap with the present proposal.

Curriculum Vitae: Saeys Wim

Positions:

2005 till present: Professor affiliated 70% at the University of Antwerp, Department of Rehabilitation Sciences and Physiotherapy
2005 till present: Physiotherapist Neurological Rehabilitation, Rehabilitation Hopsital Revarte 50%

2016 till present: Vice-chair of the Center of Health and Technology (ChaT) of the University of Antwerp
2022: coordinator of the 4D4A movement analysis lab (Located at Revarte Rehabilitation Hospital)

Education/Training

INSTITUTION AND LOCATION	DEGREE	COMPLETION	FIELD OF STUDY
Artesis College of University	Master in Physiotherapy	2005	Rehabilitation
University of Brussels (Vrije Universiteit Brussel)	Master in Neurological	2006	Rehabilitation
University of Antwerp	Doctor in Medical	2012	Medicine and

Honors (selected)

2020 United States of America: De Luca Foundation Visuospatial neglect in stroke

2019 Belgium Belfius Smart Technology Award

2017 China Editoral Board "Modern Medicine and Health Magazine" Award for Honorary Member of the 5th Editoral Board of "Modern Medicine and Health Magazine"

Five relevant publications within the last five years (* indicates trainee, # indicates correspondingauthor):

- Van Criekinge T*, **Saeys W#**, Hallemans A, Herssens N, Lafosse C, Van Laere K, Dereymaeker L, Van TicheltE, De Hertogh W, Truijen S. SWEAT2 study: effectiveness of trunk training on muscle activity after stroke. A randomized controlled trial. Eur J Phys Rehabil Med. 2021 Aug;57(4):485-494. doi: 10.23736/S1973- 9087.20.06409-6.
- Abdullahi A*, Truijen S, Umar NA, Useh U, Egwuonwu VA, Van Criekinge T, **Saeys W#**. Effects of Lower Limb Constraint Induced Movement Therapy in People With Stroke: A Systematic Review and Meta-Analysis. Front Neurol. 2021 Mar 23;12:638904. doi: 10.3389/fneur.2021.638904.
- Abdullahi A*, Candan SA, Soysal Tomruk M, Yakasai AM, Truijen S, **Saeys W#**. Constraint-induced movement therapy protocols using the number of repetitions of task practice: a systematic review of feasibility and effects. Neurol Sci. 2021 Jul;42(7):2695-2703. doi: 10.1007/s10072-021-05267-2.
- Herssens N*, **Saeys W**, Vereeck L, Meijer K, van de Berg R, Van Rompaey V, McCrum C, Hallemans A#. Anexploratory investigation on spatiotemporal parameters, margins of stability, and their interaction in bilateral vestibulopathy. Sci Rep. 2021 Mar 19;11(1):6427. doi: 10.1038/s41598-021-85870-7.
- Herssens N*, van Criekinge T, **Saeys W**, Truijen S, Vereeck L, van Rompaey V, Hallemans A#. An investigation of the spatio-temporal parameters of gait and margins of stability throughout adulthood. J R Soc Interface. 2020 May;17(166):20200194. doi: 10.1098/rsif.2020.0194.

Patents

patent for “COMPUTER IMPLEMENTED METHOD AND SYSTEM FOR MAPPING SPATIAL ATTENTION” December 8, 2021 PCT/EP2021/084817 (Pending)

Relevant funding – Grants applied for:

4D scanner and expertisecenter (4D4A) for movements analyses and valorisation – FWO Medium-Scale Infrastructure 2020

Unravelling the relationship between brain structural connectivity and gait outcome in stroke survivors: a deeper look into longitudinal gait recovery. BOF DOCPRO4 2021

Can stroke survivors re-learn normal walking? Understanding functional recovery and effects of exoskeleton-assisted training. FWO SB grant 2019

ERA-NET NEURON Call 2022
Pre-proposal: Budget plan of the project

	Coordinator	Partner 2	Partner 3	
**Name (group leader)**	Dr. Pablo Herrero Gallego<br>pherrero@unizar.es	Dr. Mindy F. Levin<br>mindy.levin@mcgill.ca	Prof Wim Saeys<br>wim.saeys@uantwerp.be	
**Institution**	IIS ARAGON	McGill University	University of Antwerp	
**Country**	SPAIN	CANADA	BELGIUM	
**Funding organisation**	INSTITUTE OF HEALTH CARLOS III - INSTITUTO DE SALUD CARLOS III (ISCIII)	FRQS	FWO	
**PROJECT COSTS (€)**	244 156 €	$254,608 CAD = 186.813 €<br>($224 999 CAD + $29 609 CAD other sources)	346 005 €	**Total**
**Personnel €**	**TOTAL: 103 500 €**<br><br>**- 57 000 €:** Researcher 18 PM x 3166,66€/month<br>**- 46 500 €:** Project Manager 18 PM x 2583,33 €/month	**TOTAL: ($179 798 CAD) = 131 923 €**<br><br>**- $24 804 CAD:** Site coordinator (0.155 FTE). (Salary = $36/hr (McGill salary scale + 22.3% benefits = $8.03/hr; $44.03 per hour = $80,013 per year).<br>**- $9 000 CAD:** Physiotherapist for dry needling: 15 patients x 12 sessions per patient = 180 sessions @$50	**TOTAL: 261 000 €**<br><br>**- 36 000 €:** Site coordinator (0.2 FTE)<br>**- 135 000 €:** Physiotherapist- DN Rx (PhD Bart Eeckhout) (1FTE)<br>**- 90 000 €:** Physiotherapist - blinded evaluator (0.5 FTE @ €45 000/yr)	**496 423 €**

		**- $48 394 CAD:** Physiotherapist blinded evaluator (30 patients x 16 assessments = 480 assessments x 2 hours = 960 hrs.Total salary = 960 hours @ $41.22 + 22.3% benefits ($9.19) = $50.41 per hour.<br>**- $97 600 CAD:** PhD trainee ($32000 year 1 and 2; $33,600 year 3).		
**Consumables €**	**TOTAL: 2 862 €**<br><br>**- 1 536 €:** Sanitary materials (needles, nitrile gloves, cotton or gauze, alcohol spray for skin asepsis, container for needles)<br>**- 1 326 €:** Electrodes 20 boxes of 100 electrodes and alcohol swabs, electrode gel, tape and bandages for each assessment	**TOTAL: ($32 898 CAD) = 24 109 €**<br><br>**- $14 400 CAD:** Patient costs - transportation for patient visits (16 assessments per patient) =16 sessions x 30 patients x $30 per session)<br>**- $2 200 CAD:** Needles for dry needling (20 boxes of 100 needles per box). Cost per box is $110.00 + sanity materials including latex gloves, alcohol spray/pads and container for needles.<br>**- $15 000 CAD:** Botox ($1000 per dose) x 15 patients<br>**- $1 298 CAD:** Electrodes 10 boxes of 100 electrodes @ $99.75= $998) and alcohol swabs, electrode gel, tape and bandages for each assessment ($300).	**TOTAL: 30 750 €**<br><br>**- 9 000 €:** Patient costs (30 x €25 per visit) x 12 visits<br>**- 7 650 €:** Transportation and costs for patient evaluations. (15 (50% patients hospital stay) x €30 per visit) x 17 visits<br>**- 1 600 €:** Needles for dry needling (20 boxes of 100 needles per box). @110 per box + sanity materials including latex gloves, alcohol spray/pads and container for needles<br>**- 10 500 €:** Botox (€700 per dose) x 15 patients<br>**- 2 000 €:** Electrodes 20 boxes of 100 electrodes @ €100 and alcohol swabs, electrode gel, tape and bandages for each assessment.	**57 721 €**

**Equipment €**	**TOTAL: 45 670 €**<br><br>**- 5 170€:** Montreal Spasticity Measure laptop computer, Procomp 5, goniometer.<br>**- 7 500€:** Sensorized insoles 30 patients per country x 250€/pair of sensorized insoles<br>**- 28 000€:** Portable system for gait analysis based on inertial IMUS.<br>**- 5 000€:** Portable ultrasound device.	**TOTAL: ($17 584 CAD) = 12 902€**<br><br>**- $7 343 CAD:** Montreal Spasticity Measure - software license, Procomp 5, goniometer<br><br>**Items covered by other sources* of funding:**<br>**- ($10 241 CAD):** Sensorized insoles 30 patients per country x 250€/pair of sensorized insoles.<br><br>* DJMP Fund of PI Levin	**TOTAL: 12 670 €**<br><br>**- 5 170€:** Montreal Spasticity Measure laptop computer, Procomp 5, goniometer<br>**- 7 500€:** Sensorized insoles 30 patients per country x 250€/pair of sensorized insoles	**71 242 €**
**Travel €[1]**	**TOTAL: 10 250 €**<br><br>- **10 250 €**. 5 Consortium meetings (2 in Spain, 2 in Belgium and 1 in Canada). 2 people:<br><br>- Kick-off meeting (Spain M1):0€<br>- 1st face-to-face Meeting (Canada M8): 4 000€<br>- 2nd face-to-face Meeting (Belgium M18): 2 500€<br>- Midterm symposium: 1 250€<br>- 3rd face-to-face Meeting (Belgium M28): 2 500€<br>- Final project meeting: (Spain M36): 0€	**TOTAL: ($ 10 000 CAD) = 7 337€**<br><br>- **$4 960 CAD**. 5 Consortium meetings (2 in Spain, 2 in Belgium and 1 in Canada). 1 person:<br><br>- Kick-off meeting (Spain M1): $500 CAD<br>- 1st face-to-face Meeting (Canada M8): $0 CAD<br>- 2nd face-to-face Meeting (Belgium M18): $250 CAD<br>- Midterm symposium: $250 CAD<br>- 3rd face-to-face Meeting (Belgium M28): $1 980 CAD<br>- Final project meeting: (Spain M36): $1 980 CAD	**TOTAL: 9 000 €**<br><br>- **9 000 €**. 5 Consortium meetings (2 in Spain, 2 in Belgium and 1 in Canada). 2 people:<br><br>- Kick-off meeting (Spain M1): 2 000€<br>- 1st face-to-face Meeting (Canada M8): 3 000€<br>- 2nd face-to-face Meeting (Belgium M18): 0€<br>- Midterm symposium: 2 000€<br>- 3rd face-to-face Meeting (Belgium M28): 0€<br>- Final project meeting: (Spain M36): 2 000€	**26 587 €**

		- * **$5 040 CAD** paid by DJMP Fund of PI Levin		
**Other direct costs €[2]**	**TOTAL: 39 500 €**<br><br>**- 14 000€:** External service consisting of API development to integrate data (anonimyzed and encrypted) from different sources (sensorized insoles, gait analysis) into the clinical data management platform used during the project.<br>**- 8 000€:** Technical support for sensorized insoles and raw data processing, delivering clinical curated data<br>**- 7 500€:** 3 open access publication as part of PhD studies (2500€ x3)<br>**- 10 000€:** Insurance for clinical trial	**TOTAL: $14 328 CAD = 10 514 €**<br><br>**Items covered by other sources of funding:**<br>**- ($10 917 CAD):** Technical support for sensorized insoles and raw data processing, delivering clinical curated data.<br>**- ($3 411 CAD):** 1 open access publication	**TOTAL: 13 000 €**<br><br>**- 8 000€:** Technical support for sensorized insoles and raw data processing, delivering clinical curated data:<br>**- 5 000€:** 2 open access publication (2x2500€)	**63 014 €**
**Overheads €[3]**	42 374€	0	19 585€	61 959€
**Total budget €[4]**	**244 156€**	**($254,608 CAD) = 186.813 €**<br>($224 999 CAD + $29 609 CAD other sources)	**346 005€**	**776 974€**
**Requested budget €[5]**	**244 156€**	**($224 999 CAD) = 165 088€**	**346 005€**	**755 249€**

Annex I : Ethical considerations

Please fill in the requested information (mandatory form).

**Section 1: HUMAN EMBRYOS/FOETUSES**		YES/NO
**Does this research involve Human Embryonic Stem Cells (hESCs)?**		NO
**If YES**:	- Will they be directly derived from embryos within this project?	
	- Are they previously established cells lines?	
**Does this research involve the use of human embryos?**		NO
**If YES**:	- Will the research lead to their destruction?	
**Does this research involve the use of human fetal tissues / cells?**		NO
**IMPORTANT:**<br>*The following are not eligible for funding under Horizon 2020:*<br>• *Research directed at human cloning for reproductive purposes;*<br>• *Research intended to modify the genetic make-up of human beings that could make such changes heritable (except research related to cancer treatment of the gonads);*<br>• *Research activities intended to create human embryos solely for the purposes of research or stem cell procurement including somatic cell nuclear transfer;*<br>• *Research that leads to the destruction of human embryos.*		
**Compliance (Brief description of compliance procedures, no upload document needed)**		
**Section 2: HUMAN SUBJECTS**		YES/NO
**Does this research involve human participants?**		YES
**If YES**:	- Are they volunteers for social or human sciences research?	NO
	- Are they healthy volunteers for medical studies?	NO
	- Are they patients?	YES
	- Are they vulnerable individuals or groups?	NO
	- Are they persons unable to give informed consent?	NO
	- Are they children/minors?	NO
**Does this research involve physical interventions on the study participants?**		YES

**If YES**:	- Does it involve invasive techniques?	YES
	- Does it involve collection of biological samples?	NO

Compliance (Brief description of compliance procedures, no upload document needed)

The involvement of Human Beings will comply with the Helsinki Declaration. We will only include people who are capable of providing informed consent and who indeed provided consent. All partners are experienced with conducting such studies in this group (people with stroke).

Dry Needling: minimal invasive technique used to treat spasticity and underlying muscular trigger points for the management of neuromusculoskeletal pain and movement impairments. It is performed with a thin (from 0,25 to 0,30 mm caliber), filiform, solid non-bevelled needle. The target structure is the neuromuscular endplate zone and the mechanism of action is mechanical.

Botulinum Toxin Type A injections: invasive technique used to treat spasticity that consists of an infiltration of neurotoxin in the muscle. It is performed with a 26 gauge (0,45 mm) beveled needle. The target structure is the neuromuscular endplate zone and the mechanism of action is chemical denervation.

**Section 3: HUMAN CELLS / TISSUES**		YES/NO
**Does this research involve human cells or tissues?** (o*ther than from Human Embryos/Foetuses, see section 1)*		NO
**If YES:**	- Are they available commercially?	
	- Are they obtained within this project?	
	- Are they obtained from another project, laboratory or institution?	
	- Are they obtained from a biobank?	

Compliance (Brief description of compliance procedures, no upload document needed)

**Section 4: PERSONAL DATA**		YES/NO
**Does this research involve personal data collection and/or processing?**		YES
**If YES:**	- Does it involve the collection and/or processing of sensitive personal data *(e.g. sexual lifestyle, ethnicity, political opinion, religious or philosophical conviction)*?	NO
	- Does it involve the collection and/or processing of Health data (e.g. Genetic or biometric information)	YES
	- Does it involve tracking or observation of participants?	YES
**Does this research involve further processing of previously collected personal data (secondary use)?**		NO

**Compliance (Brief description of compliance procedures, no upload document needed)**<br>Data related to sex, date of birth, type of stroke, location of stroke, date of stroke as well as results of clinical assessments will be collected. All data will be anonymized and stored on a secure server at each clinical site. Data sharing agreements will be created and only anonymized data will be shared.		
**Section 5: ANIMALS**		YES/NO
**Does this research involve animals?**		NO
**If YES:**	- Are they vertebrates?	
	- Are they non-human primates (NHPs)?	
	- Are they genetically modified?	
	- Are they cloned farm animals?	
	- Are they endangered species?	
Please indicate the species involved		
**Compliance (Brief description of compliance procedures, no upload document needed)**		
**Section 6: Non-EU COUNTRIES**		YES/NO
**In case non-EU countries are involved, do the research related activities undertaken in these countries raise potential ethics issues?**<br>Canada		**NO**
**Is it planned to use local resources (e.g. animal and/or human tissue samples, genetic material, live animals, human remains, materials of historical value, endangered fauna or flora samples, etc.)?**		**NO**
**Is it planned to import any material – including personal data – from non-EU countries into the EU?**		**NO**
**If Yes**:	*Specify material and countries involved*	
**Is it planned to export any material – including personal data –from the EU to non-EU countries?**		**NO**
**If Yes**:	*Specify material and countries involved*	
**In case this research involves <u>low and/or lower-middle income countries</u>, are any benefit-sharing actions planned?**		**NO**

**Could the situation in the country put the individuals taking part in the research at risk?**	**NO**
**Compliance (Brief description of compliance procedures, no upload document needed)**<br>No personal data will be shared by any of the research sites. All data will be anonymized and uploaded onto a secure server for data sharing.	
**Section 7: ENVIRONMENT & HEALTH AND SAFETY**	YES/NO
**Does this research involve the use of elements that may cause harm to the environment, to animals or plants?**	**NO**
**Does this research deal with endangered fauna and/or flora/protected areas?**	**NO**
**Does this research involve the use of elements that may cause harm to humans, including research staff?**	**NO**
**Compliance (Brief description of compliance procedures, no upload document needed)**	
**Section 8: ARTIFICIAL INTELLIGENCE**	**YES**/NO
**Does this activity involve the development, deployment and/or use of Artificial Intelligence-based systems?**	**NO**
**Section 9: OTHER ETHICS ISSUES**	YES/NO
**Are there any other ethics issues that should be taken into consideration (e.g misuse[2] of research results)?**<br>*If yes, please specify:*	**NO**

Legal references which should be used during the ethics evaluation process

Clinical Trials
Regulation 536/2014 of the European Parliament
Commission Directive 2005/28/EC

Human genetic material and biological samples
Directive 2004/23/EC

Use of animals
Directive 2010/63; Council Directive 98/58/EC; Council Directive 2008/120/EC; Council Directive 2008/119; Council Directive 2007/43; Council Regulation (EC) 1/2005; Council Regulation 1099/2009

Data Protection
Directive 95/46/EC is repealed with effect from 25 May 2018
Regulation (EU) 2016/679 on the protection of natural persons and processing personal data and its free movement (as from 25/05/2018)

Developing countries and politically sensitive issues
Declaration/Charter (EU Fundamental Rights; UN Rights of Child, UNESCO Universal Declaration

[2] Guidance note – Potential misuse of research results:
http://ec.europa.eu/research/participants/data/ref/h2020/other/hi/guide_research-misuse_en.pdf

Environmental Protection and safety
Directive 2001/18/EC; Directive 2009/41/EC; Regulation EC No 1946/2003; Directive 2008/56/EC; Council Directive 92/43/EEC; Council Directive 79/409/EEC and Council Regulation EC No 338/97

Dual use in the context of security/dissemination
Council Regulation (EC) 428/2009

Access to Genetic Resources
The Nagoya Protocol, Secretary-G

***Multinational and translational research projects on
Cerebrovascular diseases including Small Vessel Disease and brain barrier dysfunction***
