# Supplementary material for: Spinal mechanisms and feasibility of Dry Needling versus Botulinum Toxin Type A in post-stroke lower limb spasticity: A proof-of-concept randomized clinical trial protocol (STROKE-POC)
Source: PLoS One. 2026 May 20;21(5):e0334571. doi: 10.1371/journal.pone.0334571 (PMC13189288; doi:10.1371/journal.pone.0334571)
Supplement: S4 File — (PDF) [file pone.0334571.s004.pdf]

Stroke-POC

**Organization**	DSW (researchers)
**Created by**	Pablo Herrero Gallego (pherrero@unizar.es)
**Based on**	Common DSW Knowledge Model, 2.4.4 (dsw:root:2.4.4)
**Project Phase**	Before Submitting the DMP
**Created at**	02 Oct 2023

Report generated by Data Stewardship Wizard <https://ds-wizard.org>

DSW

Summary Report

Summary

Answered (current phase)	159 / 162
Answered	222 / 232

Metric	Score
Findability	0.83
Accessibility	0.64
Interoperability	0.60
Reusability	0.78
Good DMP Practice	0.79
Openness	1.00

I. Administrative information

Summary

Answered (current phase)	21 / 23	
Answered	42 / 44	

Questions

1

Contributors

Horizon 2020 DMP | maDMP | Science Europe DMP | Horizon Europe DMP

Each person contributing to creating or executing the data management plan should be added as a contributor. A project probably should have a Contact Person, and a Data Curator.

Answers

1.a.1

Name

Horizon 2020 DMP | maDMP | Science Europe DMP | Horizon Europe DMP

✔ Pablo Herrero Gallego

1.a.2

E-mail address

Horizon 2020 DMP | maDMP | Science Europe DMP | Horizon Europe DMP

✔ pherrero@unizar.es

1.a.3

ORCID Identifier

Horizon 2020 DMP | maDMP | Science Europe DMP | Horizon Europe DMP

✔ 0000-0002-9201-0120

1.a.4

Affiliation

Horizon 2020 DMP | Science Europe DMP | Horizon Europe DMP

✔

Instituto de Investigación Sanitaria Aragón

ROR https://ror.org/03njn4610

1.a.5

Role

Horizon 2020 DMP | maDMP | Science Europe DMP | Horizon Europe DMP

Roles in a project should be given as they are defined by datacite.

You should specify at least one "Contact Person". If your project has a work package for data management, identify the leader of that work package as "Data Curator".

- [x] Contact Person
- [ ] Data Collector
- [ ] Data Curator
- [ ] Data Manager
- [ ] Data Protection Officer
- [ ] Data Steward
- [ ] Distributor
- [ ] Editor
- [ ] Producer
- [x] Project Leader
- [ ] Project Manager
- [x] Project Member
- [x] Researcher

- [ ] Rights Holder
- [ ] Sponsor
- [x] Supervisor
- [ ] Work Package Leader
- [ ] Other

1.b.1

Name

Horizon 2020 DMP | maDMP | Science Europe DMP | Horizon Europe DMP

✔ Clara Pujol Fuentes

1.b.2

E-mail address

Horizon 2020 DMP | maDMP | Science Europe DMP | Horizon Europe DMP

✔ clara.pujol.fisioterapeuta@gmail.com

1.b.3

ORCID Identifier

Horizon 2020 DMP | maDMP | Science Europe DMP | Horizon Europe DMP

✔ 0000-0002-1736-3209

1.b.4

Affiliation

Horizon 2020 DMP Science Europe DMP Horizon Europe DMP

✔

Instituto de Investigación Sanitaria Aragón

ROR https://ror.org/03njn4610

1.b.5

Role

Horizon 2020 DMP maDMP Science Europe DMP Horizon Europe DMP

Roles in a project should be given as they are defined by datacite.

You should specify at least one "Contact Person". If your project has a work package for data management, identify the leader of that work package as "Data Curator".

- [ ] Contact Person
- [ ] Data Collector
- [ ] Data Curator
- [ ] Data Manager
- [ ] Data Protection Officer
- [ ] Data Steward
- [ ] Distributor
- [ ] Editor
- [ ] Producer
- [ ] Project Leader
- [x] Project Manager
- [ ] Project Member
- [x] Researcher
- [ ] Rights Holder
- [ ] Sponsor
- [ ] Supervisor
- [ ] Work Package Leader
- [x] Other

1.c.1

Name

Horizon 2020 DMP | maDMP | Science Europe DMP | Horizon Europe DMP

✔ Mindy F. Levin

1.c.2

E-mail address

Horizon 2020 DMP | maDMP | Science Europe DMP | Horizon Europe DMP

✔ mindy.levin@mcgill.ca

1.c.3

ORCID Identifier

Horizon 2020 DMP | maDMP | Science Europe DMP | Horizon Europe DMP

✔ 0000-0002-8965-7484

1.c.4

Affiliation

Horizon 2020 DMP | Science Europe DMP | Horizon Europe DMP

✔

McGill University

ROR https://ror.org/01pxwe438

1.c.5

Role

Horizon 2020 DMP | maDMP | Science Europe DMP | Horizon Europe DMP

Roles in a project should be given as they are defined by datacite.

You should specify at least one "Contact Person". If your project has a work package for data management, identify the leader of that work package as "Data Curator".

- [x] Contact Person
- [ ] Data Collector
- [ ] Data Curator
- [ ] Data Manager
- [ ] Data Protection Officer
- [ ] Data Steward
- [ ] Distributor
- [ ] Editor
- [ ] Producer
- [ ] Project Leader
- [ ] Project Manager
- [x] Project Member
- [x] Researcher
- [ ] Rights Holder
- [ ] Sponsor
- [ ] Supervisor
- [x] Work Package Leader
- [ ] Other

1.d.1

Name

Horizon 2020 DMP maDMP Science Europe DMP Horizon Europe DMP

✔ Wim Saeys

1.d.2

E-mail address

Horizon 2020 DMP maDMP Science Europe DMP Horizon Europe DMP

✔ wim.saeys@uantwerpen.be

1.d.3

ORCID Identifier

Horizon 2020 DMP maDMP Science Europe DMP Horizon Europe DMP

✔ 0000-0001-8193-5016

1.d.4

Affiliation

Horizon 2020 DMP Science Europe DMP Horizon Europe DMP

✔

University of Antwerp

ROR https://ror.org/008x57b05

1.d.5

Role

Horizon 2020 DMP maDMP Science Europe DMP Horizon Europe DMP

Roles in a project should be given as they are defined by datacite.

You should specify at least one "Contact Person". If your project has a work package for data management, identify the leader of that work package as "Data Curator".

- [x] Contact Person
- [x] Data Collector
- [ ] Data Curator
- [x] Data Manager
- [ ] Data Protection Officer
- [ ] Data Steward
- [ ] Distributor
- [ ] Editor
- [ ] Producer
- [ ] Project Leader
- [x] Project Manager
- [x] Project Member
- [ ] Researcher

- [ ] Rights Holder
- [ ] Sponsor
- [ ] Supervisor
- [ ] Work Package Leader
- [ ] Other

1.e.1

Name

Horizon 2020 DMP | maDMP | Science Europe DMP | Horizon Europe DMP

✔ Bart Eeckhaut

1.e.2

E-mail address

Horizon 2020 DMP | maDMP | Science Europe DMP | Horizon Europe DMP

✔ Bart.Eeckhaut@uantwerpen.be

1.e.3

ORCID Identifier

Horizon 2020 DMP | maDMP | Science Europe DMP | Horizon Europe DMP

✔ 0009-0001-2126-1782

1.e.4

Affiliation

Horizon 2020 DMP | Science Europe DMP | Horizon Europe DMP

✔

University of Antwerp

ROR https://ror.org/008x57b05

1.e.5

Role

Horizon 2020 DMP | maDMP | Science Europe DMP | Horizon Europe DMP

Roles in a project should be given as they are defined by datacite.

You should specify at least one "Contact Person". If your project has a work package for data management, identify the leader of that work package as "Data Curator".

- [x] Contact Person
- [x] Data Collector
- [ ] Data Curator
- [ ] Data Manager
- [ ] Data Protection Officer
- [ ] Data Steward
- [ ] Distributor
- [ ] Editor
- [ ] Producer
- [ ] Project Leader
- [ ] Project Manager
- [x] Project Member
- [ ] Researcher
- [ ] Rights Holder
- [ ] Sponsor
- [ ] Supervisor
- [ ] Work Package Leader
- [ ] Other

2
Research Project(s)

Horizon 2020 DMP | maDMP | Science Europe DMP | Horizon Europe DMP

Add each of the research project(s) that you are (or will be) working on and for which the data and work are described in this DMP. Give each project a small identifying name for yourself.

Answers

2.a.1
Project name

Horizon 2020 DMP | maDMP | Science Europe DMP | Horizon Europe DMP

✔ Comparative study of the mechanism of action of Dry Needling and Botulinum Toxin type A as a treatment for lower limb post-stroke spasticity: a proof of concept controlled trial

2.a.2
Project acronym

Horizon 2020 DMP | Horizon Europe DMP

✔ Stroke-POC

2.a.3
Project abstract

Horizon 2020 DMP | maDMP | Science Europe DMP | Horizon Europe DMP

✔ Rationale: Cerebrovascular Accident (CVA) is one of the main causes of morbidity and disability worldwide. A frequent consequence of stroke is spasticity in the affected limbs. Spasticity is a velocity-dependent increase in muscle reflex activity that affects both resting muscle tone (hypertonicity) and voluntary movements. Lower limb spasticity is associated with limitations in walking ability, resulting in an increased incidence of falls, a reduced quality of life and greater caregiver burden. Two treatments for post-stroke spasticity are injection of botulinum neurotoxin (BTX A) and dry needling (DN) to chemically or mechanically disrupt signal transmission at the neuromuscular junction in the affected muscle respectively. Both treatments reduce spasticity and improve functional walking. As BTX A injection has some adverse effects, DN may be an effective, minimally-invasive, non-pharmacological alternative to the more invasive chemical denervation.

However, while some local (muscle) mechanisms of action of BTX A and DN have been described, there is little information about their actions at the central (spinal) level, on activity, quality of life and cost-effectiveness. Objectives: The primary objective is to determine the mechanisms of action of BTX A infiltration and DN on lower limb post-stroke spasticity at the central (spinal) level. The secondary objectives are to determine safety and feasibility of each treatment and their effects at muscle and functional levels, quality of life and cost-effectiveness. The primary hypothesis is that DN treatment will be comparable to BTX A and will decrease post-stroke spasticity by decreasing stretch reflex excitability at the central (spinal) level. Improving knowledge of the mechanisms of action of both interventions will lead to more informed treatment prescription and better clinical mobility outcomes for post-stroke patients. Methods: This prospective study will compare BTX A and DN treatment on spasticity relief in people who have sustained a first stroke 3-12 months previously and who have plantar flexor spasticity. Ninety patients will be recruited from 3 centers (30 per site) in Spain, Belgium and Canada. We will use a multiple-baseline time-series design across pairs of subjects matched for age and time since stroke. BTX A will be injected once and DN will be applied once weekly for 12 weeks. Effects will be evaluated before, during and after treatment and at a 4 week follow-up by blinded evaluators. Effects on spasticity will be evaluated at the central (spinal) level using a physiological measure of motoneuronal excitability (Tonic Stretch Reflex Threshold and its velocity sensitivity) and at the muscle level by quantifying morphological changes with ultrasound imaging and the perceived resistance to stretch (Mod-Mod Ashworth Scale). We will also assess effects on gait (Timed Up and Go, 10 Meter Walk Test and instrumented gait analysis) and quality of life (EuroQOL-5D). Cost-effectiveness of each intervention will be determined. A patient Advisory Group will be created to engage patients by asking for feedback during the study and to contribute to results dissemination. Outcomes: Although BTX is the gold standard for post-stroke spasticity treatment, DN treatment has shown to be effective with potentially fewer adverse effects. However, DN has not yet been routinely implemented in clinical practice and there are no comparative studies with BTX A and mechanisms of action remain unknown. This impedes prescription of the best available treatment to post-stroke patients considering spasticity pathophysiology. Results of this feasibility study (proof of concept) will determine the mechanisms of action of both treatments so that future studies with larger samples and other neuropathologies can be developed. ● Lay Abstract Stroke is one of the main causes of disease and disability in the world and has a significant economic impact on society. As populations grow and people live longer, the number of cases of stroke is expected to rise. Stroke affects the central nervous system and interferes with the ability to move and walk. Half of all stroke victims develop spasticity (stiffness in the muscles) within six months, which may cause discomfort and interfere with the ability of people to return to everyday life activities. There are several treatment possibilities for alleviating spasticity. One of the most effective treatments is the injection of Botulinum Toxin type A (BTX A). However, this treatment is considered to be ‘invasive’ and it has been linked to several side effects. A relatively new treatment for spasticity with comparable effectiveness as BTX A, is a technique called ‘dry needling’. This technique does not require the use of drugs and is safe, minimally invasive and has fewer side effects. However, the use of dry needling is not very common in current clinical practice. Some studies have reported the effects of these two techniques on the muscle and on the patients’ ability to move but their effects across the whole system have not been systematically studied and there are few comparative studies of their effects. We propose that dry needling and BTX A treatments will have comparative effects on decreasing spasticity but that dry needling will have fewer side effects and be more acceptable as a

treatment option for patients and their families. We will study this question by examining the effects of each treatment in ankle muscles in two groups of subjects. One group will have a series of 12 dry needling sessions and the other will have one injection of BTX A. We will evaluate the effects of the treatment on spasticity at several different levels –muscle, reflex activity, motor ability, quality of life and whether the treatment is found to be acceptable and cost-effective. We will use novel methods to measure the effects that will provide us with new information to help clinicians together with patients and their families make more informed choices about spasticity treatment options.

2.a.4

Link to a project proposal or another description of the methods used in the project

✔ https://www.neuron-eranet.eu/projects/STROKE-POC/

2.a.5

Starting date of the project

Horizon 2020 DMP | Science Europe DMP | maDMP | Horizon Europe DMP

✔ 2023-04-01

2.a.6

Ending date of the project

Horizon 2020 DMP | Science Europe DMP | maDMP | Horizon Europe DMP

✔ 2026-03-31

2.a.7

Funding

Horizon 2020 DMP | maDMP | Science Europe DMP | Horizon Europe DMP

Add all the funding that are part of this project.

Answers

2.a.7.a.1

Funder

Horizon 2020 DMP | Science Europe DMP | maDMP | Horizon Europe DMP

Specify the name of the funder that you ask for funding for your project. If the funder is not present in the suggested list, please specify a complete URL to the funder web site.

✔

Horizon 2020

Crossref http://dx.doi.org/10.13039/501100007601

2.a.7.a.2

Funding status

Horizon 2020 DMP | Science Europe DMP | maDMP | Horizon Europe DMP

✔ c. Granted

2.a.7.a.3

Grant number

Horizon 2020 DMP | maDMP | Science Europe DMP | Horizon Europe DMP

✔ NEURON_CV-082

3

To execute the DMP, is additional specialist expertise required?

Horizon 2020 DMP | Science Europe DMP | Horizon Europe DMP

✔ b. Yes, trained support staff is available

4

Do you require hardware or software in addition to what is usually available in the institute?

Horizon 2020 DMP Horizon Europe DMP

✔ b. Yes

4.b.1

What specific hard/software do you need, and why?

Horizon 2020 DMP Science Europe DMP Horizon Europe DMP

✔ DS-Wizard will be used to create and update the DMP

5

Describe national / funder / sectorial / departmental policies and procedures for data management that you will be using.

Horizon Europe DMP

Answers

5.a.1

Name of the policy or process

Horizon Europe DMP

✔ GDPR

5.a.2

Give a link/reference to the policy or process

Horizon Europe DMP

✘ *This question has not been answered yet!*

5.a.3

Description of how and why you are using this policy or process

Horizon Europe DMP

✖ *This question has not been answered yet!*

II. Re-using data

Before you decide to embark on any new study, it is good practice to check all options to re-use existing available data, either collected or generated by yourself in an earlier project, or data from others (Barend Mons calls this "Other PEople's Data And Services" or OPEDAS). This can include reusable data that have been created for an earlier study, and also so-called "reference data" which is used by many projects.

It is not because we can generate massive amounts of data that we always need to do so. Creating data with public money is bringing with it the responsibility to treat those data well and (if potentially useful) make them available for re-use by others. And the circle is only complete if such data is actually re-used.

Summary

Answered (current phase)	1 / 1	
Answered	2 / 2	

Questions

1

Do you need guidance to find existing data?

Research funding organisations more and more demand that you search for existing data sets that could have information that you need, before assuming you need to collect all data yourself. You are asked to list what you have been able to locate, and whether you have found it suitable to use in your research. Do you need pointers to find such existing data sets?

✔ a. No

2

Is there any pre-existing data?

Horizon Europe DMP Horizon 2020 DMP maDMP Science Europe DMP

Are there any data sets available in the world that are relevant to your planned research?

Data Stewardship for Open Science: *atq*
External Links: *Google dataset search*, *Datacite Search*, *RDMkit on Reusing Data*, *RDMkit on Existing Data*

✔ a. No

It is possible that you have overlooked existing data! This question is not only about data sets that are similar to what you want to determine yourself, but also reference data or data that should be mined from the existing literature. Further, it is very likely that you will refer to related data, e.g. other databases where you usually "quickly look something up", but that could maybe be properly integrated, especially if you need to do such lookups multiple times.

III. Creating and collecting data

In this chapter we describe all the sources of data: they can e.g. come from instruments or from questionnaires; data can be newly collected as part of the current project, but it can also be pre-existing data that may need proper contracts with the maintainer, some pre-processing, and quality checks. It can also be reference data that is part of curated resources and (public) databases.

For more information see Collecting in RDMKit

Summary

Answered (current phase)	69 / 69
Answered	101 / 103

Metric	Score
Findability	0.80
Accessibility	0.00
Interoperability	0.75
Reusability	0.76
Good DMP Practice	0.75

Questions

1

Are you running the project in a collaboration between different groups or institutes?

✔ b. Yes

1.b.1

Is there a collaboration agreement in the project that describes who can have access to what data?

External Links: *See also description of Data Sharing in RDMkit*

✔ b. Yes

2

Will you be collecting physical samples?

Will you be collecting artefacts like specimens, minerals, biological samples?

Data Stewardship for Open Science: *kuz*

✔ a. No

3

How will you do file naming and file organization?

Horizon 2020 DMP | Horizon Europe DMP

Putting some thoughts into file naming can save a lot of trouble later.

External Links: *RDMkit on data organisation*

✔ a. Explore

3.a.1

Did you make appointments in the project on how to name files and folders?

Horizon 2020 DMP | Horizon Europe DMP

It can help if everyone in the project uses the same naming scheme.

✔ b. Yes

3.a.1.b.1

How do you name files in the project?

Horizon 2020 DMP | Horizon Europe DMP

Describe how everyone in the project will be naming files and folders, and what folder structure you will use.

✖ *This question has not been answered yet!*

3.a.2

Will you be keeping the relationships between data clear in the file names?

Horizon 2020 DMP Horizon Europe DMP

Advice: Use the same identifiers for sample IDs etc throughout the entire project.

✔ b. Yes

3.a.3

Will all the metadata that is embedded in the file names also be available in the proper metadata?

Horizon 2020 DMP Horizon Europe DMP

The file names are very useful as metadata for people involved in the project, but to computers they are just identifiers. To prevent accidents with e.g. renamed files metadata information should always also be available elsewhere and not only through the file name.

Also note that if metadata could need to change, embedding it in the file names may require renaming files during the project; and this may have implications for references to those files.

✔ b. Yes, all metadata is also explicitly available elsewhere

3.a.4

Will you be using persistent identifiers to refer to data within the project?

Horizon 2020 DMP

Especially for large projects, referring to data internally via a persistent identifier system can be helpful as such a system can help to keep track of data that moves to a new location.

External Links: *The Handle System*, *Handbook on Persistent Identifiers*

✔ b. Yes

4

Do you need guidance on what data formats/types to use?

In many research fields there are standard file formats and types that have been established by disciplinary standards organisations. Using such standards can help increase the interoperability of your data with other data and tools. Do you need help finding the right standards to use in your research?

✔ b. Yes

You can find a lot of information on standard data formats in [FAIRsharing]

4.b.1

Will you need data formats for Natural Sciences

We are interested to receive your expertise on this topic. You can use the feedback button if you have resources that we could list here

✔ a. No

4.b.2

Will you need data formats for Engineering and Technology?

✔ a. No

4.b.3

Will you need data formats for Medical and Health Sciences?

✔ b. Yes

4.b.3.b.1

Will you need data formats for toxicology research?

✔ a. No

4.b.4

Will you need data formats for Agricultural Sciences?

✔ a. No

4.b.5

Will you need data formats for Social Sciences?

✔ a. No

4.b.6

Will you need data formats for Humanities?

✔ a. No

5

What existing data formats/types will you be using?

Horizon 2020 DMP | Science Europe DMP | Horizon Europe DMP

Have you identified types of data that you will use that are used by others too? Some types of data (for example "images" or "tables") are used by many different projects. For such data, often common standards exist (in our example "JPG" and "CSV" [comma separated values]) that help to make these data reusable. Are you using such common data formats?

Please make sure you list all the data types that are important for your project. You should make sure also to list the formats used in any data sets that you are re-using.

Data Stewardship for Open Science: *njy*

Answers

5.a.1

Data format/type

Horizon 2020 DMP Science Europe DMP Horizon Europe DMP

✔

Common European Research project Information Format

FAIRsharing https://fairsharing.org/10.25504/FAIRsharing.6945dq

5.a.2

Is this a standard data format widely used by researchers in this field?

Horizon 2020 DMP Science Europe DMP Horizon Europe DMP

✔ b. Yes

5.a.3

Does this data format enable sharing and long term archiving?

Horizon 2020 DMP Science Europe DMP Horizon Europe DMP

Complicated (binary) file formats tend to change over time, and software may not stay compatible with older versions. Also, some formats (e.g. DOC, XLS) hamper long term usability by making use of patents or being hampered by restrictive licensing.

Ideally a format should be simple, text only, completely described, not restricted by copyrights, and implemented in different software packages.

✔ b. Yes

5.a.4

What volume of data of this type will you be working with?

Horizon 2020 DMP Science Europe DMP Horizon Europe DMP

✔ a. So small that it is not a problem

5.a.5

Is this data format completely described?

Formats like XLS or SQL are very flexible; they can be adapted to many different uses, and this makes them good for interoperability. However, their flexibility also makes that it is not immediately obvious from the file structure how it can be used. The data needs a proper *description* in order for others (or yourself at a later time) to be able to unambiguously understand what it contains.

✔ b. Yes

6

Do you need guidance on what encodings/terminologies/vocabularies/ontologies to use?

✔ b. Yes

6.b.1

Will you need terminologies in Natural Sciences?

✔ a. No

6.b.2

Will you need terminologies in Engineering and Technology

✔ a. No

6.b.3

Will you need terminologies in Medical and Health Sciences?

✔ b. Yes

Biomedical ontologies can be found at:

- BioPortal
- EBI Ontology Lookup Service

6.b.3.b.1

Will you need terminologies in toxicology research?

✔ a. No

6.b.4

Will you need terminologies in Agricultural Sciences?

✔ a. No

6.b.5

Will you need terminologies in Social Sciences?

✔ a. No

6.b.6

Will you need terminologies in Humanities?

✔ a. No

7

What existing encodings/terminologies/vocabularies/ontologies will you be using?

Horizon 2020 DMP Horizon Europe DMP

Answers

7.a.1

Name

Horizon 2020 DMP Horizon Europe DMP

✔

Common European Research project Information Format

FAIRsharing https://fairsharing.org/10.25504/FAIRsharing.6945dq

7.a.2

If you use a standard that is not in FAIRsharing, please specify its PID or URL

Horizon 2020 DMP Horizon Europe DMP

✖ *This question has not been answered yet!*

8

Will you be using new types of data?

Horizon 2020 DMP Horizon Europe DMP

Sometimes the type of data you collect can not be stored in a commonly used data format. In such cases you may need to make your own, keeping interoperability as high as possible.

Data Stewardship for Open Science: *ikk*

✔ a. No, all of my data will fit in common formats

9

How will you be collecting and keeping your metadata?

Science Europe DMP Horizon 2020 DMP Horizon Europe DMP

For the re-usability of your data by yourself or others at a later stage, a lot of information about the data – for example how it was collected and how it can be used – should be stored with the data. Such *data about the data* is called **metadata**, and this set of questions are about this metadata.

Data Stewardship for Open Science: *rhm*
External Links: *RDMkit on documentation and metadata*, *Metadata Standards Catalogue (RDA)*

✔ a. Explore

There are many kinds of metadata, each serving their own purpose. Some key metadata that you should consider:

- There is metadata that helps identify where the data is coming from (e.g. who created it, title). For this the Dublin Core is often used.
- There are different ways of adding metadata to make the data "discoverable" for other researchers. This requires either keywords or ontology terms describing what is in the data.
- There is metadata describing how the data can be re-used, such as license information and, for data about people, the extent of their consent for data reuse.
- There is metadata that makes the data understandable, e.g. linking to the exact processes used to collect them (is a *body temperature* measured under the tongue or in the rectum?) and units (is a temperature given in Celsius or Fahrenheit?).
- There is metadata describing where the data comes from and what it is useful for. For frequently used data types, there are often very well defined metadata standards, in other cases you may need to think about this yourself. For each of these kinds of metadata there are specific standards. There is no single standard that will get you all the metadata needed to make the data as FAIR as possible.

9.a.1

What standard(s) will you use to specify author/title/keyword information?

Horizon 2020 DMP | Science Europe DMP | Horizon Europe DMP

There are a few different standards that are often used to give basic information about your dataset. Which ones of these will you be using?

✔ a. Explore

9.a.1.a.1

Will you document the data with Dublin Core metadata?

Horizon 2020 DMP | Science Europe DMP | Horizon Europe DMP

Dublin Core is a standard documenting domain independent aspects of a resource; including who has created it, audience, function, formatting and licensing. Does your documentation follow the Dublin Core standard?

External Links: *Dublin Core Metadata Terms*, *Dublin Core Initiative*

✔ b. Yes

9.a.1.a.2

Will you document the data with DataCite metadata

Science Europe DMP | Horizon 2020 DMP | Horizon Europe DMP

External Links: *DataCite metadata schema*

✔ b. Yes

9.a.1.a.3

Will you document the data with DDI metadata

Horizon 2020 DMP | Science Europe DMP | Horizon Europe DMP

DDI metadata is more extensive than Dublin Core and DataCite, it details more of what is in the data and really can help other researchers locate your data set as an interesting source.

External Links: *DDI metadata documentation*

✔ a. No

9.a.1.a.4

Will you be including keywords or relevant ontology references to optimise the possibility for discovery and potential reuse?

Horizon Europe DMP

✔ b. Yes

9.a.2

Do suitable 'Minimal Metadata About ...' (MIA...) standards exist for your experiments?

Horizon 2020 DMP Horizon Europe DMP

Many research fields have worked together to define what kind of metadata should really be collected when an experiment of a certain kind is performed and described. That information is described in a Minimal Metadata Standard. Often, these standards describe both what kind of information needs to be collected as well as the format in which it is expected.

External Links: *FAIRsharing repository of standards*

✔ a. No

Did you really check a service like fairsharing.org to verify this?

9.a.2.a.1

Do you have a good idea of what metadata is needed to make it possible for others to read and interpret your data in the future?

Horizon 2020 DMP Horizon Europe DMP

Imagine yourself trying to interpret a similar data set from a colleague in a few years. Is there any way in which the data could be misinterpreted? Does everyone in the field do things in exactly the same way? And will that still be known in 10 years? How can such misinterpretation be avoided?

External Links: *FAIRsharing repository of standards*

✔ b. Yes

9.a.3

Do you know how and when you will be collecting the necessary metadata?

Often it is easiest to make sure you collect the metadata as early as possible.

External Links: *FAIRsharing repository of standards*

✔ b. Yes

9.a.4

Will you consider re-usability of your data beyond your original purpose?

Adding more than the strict minimum metadata about your experiment will possibly allow more wide re-use of your data, with associated higher data citation rates. Please note that it is not easy for yourself to see all other ways in which others could be reusing your data.

✔ b. Yes, I will add "optional" metadata where I can

9.a.4.b.1

How will you balance the extra efforts with the potential for added reusability?

✔ a. I will see what I can do

9.a.4.b.2

Do you need to exchange your data with others?

✔ b. Yes

9.a.5

Will a license be assigned to your datasets?

It is not always clear to everyone in the project (and outside) what can and can not be done with a data set. It is helpful to associate each data set with a license as early as possible in the project. A data license should ideally be as free as possible: any restriction like 'only for non-commercial use' or 'attribution required' may reduce the reusability and thereby the number of citations. If possible, use a computer-readable and computer actionable license.

External Links: *RDMkit on Licensing*

✔ b. Yes

9.a.5.b.1

Will you store the licenses with the data at all time?

It is very likely that data will be moved and copied. At some point people may lose track of the origins. It can be helpful to have the licenses (of course *as open as possible*) stored in close association with the data.

Data Stewardship for Open Science: *atw*

✔ b. Yes

9.a.6

Where do you keep any applicable restrictions on the usage of the data?

External Links: *Data Use Ontology*

✔ c. Stored in the same folder as the data

9.a.7

How will you be keeping track of the "provenance" of the data?

Horizon 2020 DMP | Science Europe DMP | Horizon Europe DMP

Data analysis is normally done step-by-step. It is important that for all data the origin and all processing and filtering steps are documented, otherwise results will not be reproducible.

Re-users of the data also need this information to decide whether the data can be used for their purpose.

In computing, systems like Galaxy and (Jupyter) notebooks often automatically keep provenance information.

✔ b. We use an electronic lab notebook

9.a.8

Will you be documenting the data with W3C PROV provenance?

Horizon 2020 DMP | Science Europe DMP | Horizon Europe DMP

The W3C Prov standard documents processes (workflow) that were used to produce a resource. This can be used to document e.g. the software (including version) and parameters you use to analyze the data. Will your documentation follow the W3C Prov standard?

External Links: [W3C Prov primer]

✗ *This question has not been answered yet!*

9.a.9

Will you use a workflow system that automatically keeps track of the steps in the analysis?

Some workflow systems automatically keep track of which steps were done in data analysis and what options were selected. This can help document the data for reproducibility.

✓ a. No

10

Will you be acquiring data using measurement equipment?

Horizon 2020 DMP | Science Europe DMP | Horizon Europe DMP

✓ b. Yes

10.b.1

Specify what data sets you will acquire using measurement equipment

Horizon 2020 DMP | Science Europe DMP | Horizon Europe DMP

You can use any name for the data set, make sure that it identifies the data set to yourself.

Answers

10.b.1.a.1

Data set:

Horizon 2020 DMP | Science Europe DMP | Horizon Europe DMP

✔ Datasets for the clinical outcomes and specific measurements of spasticity and muscle ultrasound

10.b.1.a.2

Who will do the measurements? And where?

Horizon 2020 DMP | Science Europe DMP | Horizon Europe DMP

Are there easily accessible specialized service providers for data capture?

✔ a. Experts in the project, with our own equipment

10.b.1.a.3

Instruments used for data collection

Science Europe DMP

Specify what technical instruments you are using to collect the data.

Answers

10.b.1.a.3.a.1

Instrument name

Science Europe DMP

✔ Montreal Stretch Reflex Threshold (MSRT)

10.b.1.a.3.a.2

Instrument description

Science Europe DMP

✔ The MSRT is a portable, clinical device consisting of a two-channel electromyography (EMG) system, an electrogoniometer and dedicated software implemented on a laptop computer

10.b.1.a.3.b.1

Instrument name

Science Europe DMP

✔ Ultrasound imaging

10.b.1.a.3.b.2

Instrument description

Science Europe DMP

✔ Deviced used to collect echo-textural and echo-structural parameters as well as M-mode (or motion mode) of the muscular ultrasound images

10.b.1.a.3.c.1

Instrument name

Science Europe DMP

✔ Xsens system (MVN Awinda, Movella, Hendersen, USA)

10.b.1.a.3.c.2

Instrument description

Science Europe DMP

✔ The Xsens system is a wireless motion capture system used for tracking human movement in real-time. The system includes the MVN Awinda, a full-body motion tracking suit that uses 17 wireless inertial sensors to capture movement data. The system is designed for use in a variety of applications, including sports performance analysis, clinical gait analysis, and animation and film production. The Xsens MVN Awinda is known for its accuracy, ease of use, and versatility. In our case we will use it for gait analysis. This system will be used in Canada and in Belgium.

10.b.1.a.3.d.1

Instrument name

Science Europe DMP

✔ Move Human Sensors MoCap System

10.b.1.a.3.d.2

Instrument description

Science Europe DMP

✔ The Move Human Sensors (MH) MoCap System is a full-body motion capture technology that uses IMU sensors to track human movement. This system is designed to aid in decision-making during treatments or therapies for individuals with motion disabilities that affect their range of motion and neural control. The MH system can be configured with magnetometer-free IMUs, which helps to overcome the limitation of the need for magnetically controlled environments. The system can be used for gait analysis, rehabilitation applications, and sports performance analysis. We will use it for gait analysis. This system will be used in Spain. .

10.b.1.a.3.e.1

Instrument name

Science Europe DMP

✔ Dinamometer

10.b.1.a.3.e.2

Instrument description

Science Europe DMP

✔ Dinamometer allows to measure muscle force

10.b.1.a.4

Is the equipment completely standard and well described?

Horizon 2020 DMP | Science Europe DMP | Horizon Europe DMP

If the technology is very much under development, you may want to come back later to understand exactly how the measurements have been made. Is the measurement equipment and protocol sufficiently standard that you will be able to explain how it is done or refer to a standard explanation?

✔ a. Very well described and known

10.b.1.a.5

Is special care needed to get the raw data ready for processing?

Where does the data come from? And who will need it? Sometimes the raw data is measured somewhere else than where the primary processing is taking place. In such cases the ingestion or transport of the primary data may take special planning. You also need to make sure that data is secure and that data integrity is guaranteed.

✔ b. Yes, lets explore this

10.b.1.a.5.b.1

Is the data format established?

Has the storage and transport format of the primary data been established between the people responsible for the measurement and the people responsible for the processing?

✔ b. Yes

10.b.1.a.5.b.2

How will the raw data be transported?

External Links: [RDMkit on Data Transfer]

✔ c. Via the network

10.b.1.a.5.b.2.c.1

Is sufficient network capacity available?

Can the volume of data be accommodated by the standard network connection? Has a special network connection (e.g. light path) that is needed been reserved?

✔ a. Yes, has been taken care of

10.b.1.a.5.b.3

Is data integrity guaranteed during this stage?

Do you have any means of identifying whether the raw data has been transferred error free and has not been tampered with?

✔ a. No

10.b.1.a.5.b.4

Is data security guaranteed during this stage?

Are the raw data encrypted or otherwise protected from theft or leaks at either site or during transport? You could e.g. use a light path or a virtual private network if you transport the data over the net.

✔ b. Yes

10.b.1.a.6

Will you be using quality processes?

Science Europe DMP Horizon Europe DMP

Specify how you guarantee that measurements measure what you think they should measure, and how you establish completeness and correctness of data and metadata.

External Links: *RDMkit on Data Quality*

✔ b. Yes

10.b.1.a.6.b.1

Are you calibrating measurements?

Science Europe DMP Horizon Europe DMP

✔ b. Yes

10.b.1.a.6.b.2

Are you running repeat samples or are you repeating measurements in order to monitor consistency of the results?

Science Europe DMP Horizon Europe DMP

✔ b. Yes

10.b.1.a.6.b.3

Are you running standardized data capture or recording?

Science Europe DMP Horizon Europe DMP

✔ b. Yes

10.b.1.a.6.b.4

Are you doing Data Entry validation?

Science Europe DMP Horizon Europe DMP

Are you checking that data that is entered e.g. in forms is satisfying criteria of reasonability?

✔ b. Yes

10.b.1.a.6.b.5

Are you using data peer review?

Science Europe DMP Horizon Europe DMP

Do you make sure all collected is cross-checked by colleagues?

✔ a. No

10.b.1.a.6.b.6

Are you using controlled vocabularies where possible?

Science Europe DMP Horizon Europe DMP

Controlled vocabularies should be used to limit what can be entered in most text fields.

✔ a. No

10.b.1.a.6.b.7

Are you using any other quality processes?

Science Europe DMP Horizon Europe DMP

✔ a. No

10.b.1.a.6.b.8

Are you measuring samples for which the outcome is known in order to monitor consistency?

Horizon Europe DMP

✔ a. No

10.b.1.a.7

Who else could be interested in using this data?

Horizon 2020 DMP Horizon Europe DMP

✔ b. Other researchers working in the same field of research

11

Do you have any non-equipment data capture?

Horizon 2020 DMP Science Europe DMP Horizon Europe DMP

Does the data you collect contain non-equipment captured data such as questionnaires, case report forms, electronic patient records?

Data Stewardship for Open Science: *ybw*

✔ b. Yes

11.b.1

Will you be collecting questionnaires?

Horizon 2020 DMP Science Europe DMP Horizon Europe DMP

✔ b. Yes

11.b.2

Will you be collecting case report forms?

Horizon 2020 DMP Science Europe DMP Horizon Europe DMP

Data Stewardship for Open Science: *hfg*

✔ b. Yes

11.b.3

Will you be collecting data from electronic patient records?

Horizon 2020 DMP Science Europe DMP Horizon Europe DMP

✔ a. No

11.b.4

Please list all non-equipment data sets you will collect

Horizon 2020 DMP | Science Europe DMP | Horizon Europe DMP

You can use any name for the data set, make sure that it identifies the data set to yourself.

Answers

11.b.4.a.1

Data set:

Horizon 2020 DMP | Science Europe DMP | Horizon Europe DMP

✔ Clinical dataset

11.b.4.a.2

Description

Horizon 2020 DMP | Science Europe DMP | Horizon Europe DMP

Briefly describe the contents of this data set

✔ A dataset containing all the clinical outcomes except the ones coming directly from the equipment

11.b.4.a.3

How will the data be captured?

✔ b. Data will first be on paper

11.b.4.a.3.b.1

Who will do the data entry?

✔ b. One of the members of the project team

11.b.4.a.3.b.2

Did you arrange who will make the data digitally available to you?

✔ a. Not yet

11.b.4.a.4

Will all data be collected by a single person?

✔ b. More people will capture data

11.b.4.a.4.b.1

Is there a risk of different interpretations? Subjectivity?

✔ b. There are internal controls to deal with interperation differences

11.b.4.a.5

Who else could be interested in using this data?

Horizon 2020 DMP Horizon Europe DMP

✔ b. Other researchers working in the same field of research

12

Is there a data integration tool that can handle and combine all the data types you are dealing with in your project?

✔ a. No

12.a.1

Can all data be brought into the same format, e.g. RDF?

a. No

13

Will you collect any data connected to a person, "personal data"?

Horizon Europe DMP

Very many kinds of data are connected to people. If there could be a way for someone, including yourself, to find out who that person is, that is considered personal data.

Simple examples are name, birth day or address; but there are many other data that can be personal: for example a voice recording, a combination of a location and a time (traffic flow), genetic information, or an X-ray of the skull.

External Links: *RDMkit about Sensitive Data*, *RDMkit on Human Data*, *RDMkit on Data Protection*

a. No

14

How is the ownership of the collected data arranged?

Horizon 2020 DMP Science Europe DMP Horizon Europe DMP

d. We have a consortium agreement that arranges Intellectual Property

15

Will you monitor data integrity once it has been collected?

Working with large amounts of heterogenous data in a larger research group can have implications for the data integrity. How do you make sure every step of the workflow is done with the right version of the data? How do you handle the situation when a mistake is uncovered? Will you be able to redo the strict minimum data handling?

Data Stewardship for Open Science: *spg*
External Links: *RDMkit on Data Quality*

a. No

If you are working with multiple people on a complex project, it could be causing problems if changes are made to e.g. sample identifiers half-way the project. If there is no central information

keeping, not everyone may be aware of important changes, and updates of partially run workflows could be forgotten.

IV. Processing data

In the processing phase, the data will be undergoing the mostly automated steps for processing, before the analysis and interpretation.

In this chapter, many questions are focusing on the compute environment that is used to process the data and make it available for interpretation by project partners. Some of those questions (e.g. on workflow systems and data provenance) are also relevant for the work in the interpretation phase.

Summary

Answered (current phase)	32 / 32
Answered	39 / 40

**Metric**	**Score**
Accessibility	1.00
Reusability	0.74
Good DMP Practice	0.85

Questions

1

Will you be using a shared working space to work with your data?

Horizon 2020 DMP **Science Europe DMP** **Horizon Europe DMP**

Will you be using a working space containing data and software specific to the project that is shared between all the people working on the data in the project? Sometimes such a system is called a *Virtual Research Environment*.

✔ a. No, participants in the project each have different collections of data and tools

1.a.1

Are data that project members and partners store themselves adequately backed up and traceable?

Science Europe DMP

✔ b. Yes, protected against both equipment failure and human error

2

Data storage systems and file naming conventions

Science Europe DMP

It is a good idea to pre-define how data will be organised in the project work space, and to set conventions for how any data files and folders will be named.

External Links: *RDMkit on data organisation*, *RDMkit on data storage*

✔ a. Explore

2.a.1

How much storage space will the project require for all data and software, including temporary storage?

✔ a. So little that it is not a problem

2.a.2

Are you using a filesystem with files and folders?

Science Europe DMP

Are some of the data in the project stored in a filesystem with files and folders?

External Links: *RDMkit on Data Organisation*

✔ b. Yes

2.a.2.b.1

Will you use a folder for each sample/subject?

Science Europe DMP

✔ a. No

2.a.2.b.2

Will you use a (sub)folder for each (repeated) analysis?

Science Europe DMP

✔ a. No

2.a.2.b.3

Will you use a (sub)folder for each step in the analysis workflow?

Science Europe DMP

✔ a. No

2.a.2.b.4

What appointments have you made about the naming of files?

Science Europe DMP

Make sure names are relatively short, and avoid spaces and special characters. You can use underscore characters, and consider using unique identifiers for the samples/experiments. You can consider to add versioning using the date in YYYYMMDD format.

✖ *This question has not been answered yet!*

2.a.2.b.5

Did you document how you manage file versioning?

✔ a. No

2.a.3

Will you be storing data in an "object store" or a "document store" system?

Science Europe DMP

Some "file" storage systems do not have a tree structure like we know in a file system, but rather have direct pointers to any file in the system. Such systems are called "object stores" or "document stores". Examples are Amazon S3 and CEPH, or MongoDB.

External Links: *Wikipedia on object storage*, *RDMkit on Data Storage*

✔ a. No

2.a.4

Will you use a database system to store project data?

Science Europe DMP

✔ a. No

2.a.5

Are you storing (some of your) data in an application specific manner?

Are you using e.g. an Electronic Lab Notebook (ELN) or Electronic Data Capture (EDC) application? Such applications often have their own data structure that can only be accessed through the application.

✔ b. Yes

Note that using the data that is stored in such a system will need to be exported in some standard way before it becomes available for other data analysis tools.

3

Workflow development

It is likely that you will be developing or modifying the workflow for data processing. There are a lot of aspects of this workflow that can play a role in your data management, such as the use of an existing work flow engine, the use of existing software vs development of new components, and whether every run needs human intervention or whether all data processing can be run in bulk once the work flow has been defined.

✔ a. This has been arranged

4

How will you make sure to know what exactly has been run?

External Links: [*RDMkit on Data Analysis*]

✔ a. Explore

4.a.1

Will you keep results together with all processing scripts or workflows including documentation of the versions of the tools that have been run?

✔ b. Yes

4.a.2

Will you make use of the metadata fields in your output data files to register how the data was obtained?

File formats like VCF (for genetics) and TIFF (for images) have possibilities to document metadata in the file header. It is a good idea to use work flow tools that use these fields to document what was done to obtain the data.

✔ b. Yes

4.a.3

Will you use a central repository for all tools and their versions as used in your project?

Especially if analysis and processing of data in the project is done on multiple different computers by different people, it is a good idea to have your own repository of tools and their blessed versions.

Data Stewardship for Open Science: *pzq*
External Links: *RDMkit on Package management systems*

✔ b. Yes

4.a.4

Will you use a central repository for reference data used in your project?

Especially if analysis and processing of data in the project is done on multiple different computers by different people, it is a good idea to have your own repository of reference data versions.

Data Stewardship for Open Science: *pzq*

✔ b. Yes

4.a.5

Will you make use of standard workflow engines and automatic work flows for all data analysis in the project?

It is much easier to guarantee consistency and reproducibility if all data processing is done using automated work flows, especially if the workflow engine automatically keeps adequate provenance data.

✔ a. No

4.a.6

Are all software tools in the work flow professionally maintained, with version control?

Will you be able to find and reproduce exactly which version was used for any analysis? Not only for the major tools in the workflows, but also for all 'glue' code and small tools you created especially for the project?

✔ b. Yes

5

How will you validate the integrity of the results?

Horizon 2020 DMP Horizon Europe DMP

✔ a. Explore

5.a.1

Will you run a subset of your jobs several times across the different compute infrastructures you are using?

Horizon 2020 DMP Horizon Europe DMP

There are surprisingly many complications that can cause (slight) inconsistencies between results when workflows are run on different compute infrastructures. A good way to make sure this does not bite you is to run a subset of all jobs on all different infrastructure to check the consistency.

✔ b. Yes

5.a.2

Will you be instrumenting the tools into pipelines and workflows using automated tools?

Horizon 2020 DMP Horizon Europe DMP

Surrounding all tools in your data processing and analysis workflows with the 'boilerplate' code necessary on the computer system you are using is tedious and error prone. Especially if you are using the same tools in multiple different work flows and/or on multiple different computer architectures. Automated instrumentation, e.g. by using a workflow management system, can prevent many mistakes.

✔ a. No

5.a.3

Will you use independently developed duplicate tools or workflows for critical steps to reduce or eliminate human errors?

Horizon 2020 DMP | Horizon Europe DMP

Validation of results without a golden standard is very hard. One way of doing it is to develop two solutions for a problem (two independent workflows or two independently developed tools) to check whether the results are identical or comparable.

✔ a. No

5.a.4

Will you run part of the data set repeatedly to catch unexpected changes in results?

Horizon 2020 DMP | Horizon Europe DMP

Running a small subset of the data repeatedly can be useful to catch unexpected problems that would otherwise be very hard to detect.

Data Stewardship for Open Science: *egv*

✔ b. Yes

6

Do you need to do compute capacity planning?

If you require substantial amounts of compute power, amounts that are not trivially absorbed in what you usually have abailable, some planning is necessary. Do you think you need to do compute capacity planning?

✔ a. No

7

Is the risk of information loss, leaks and vandalism acceptably low?

Horizon 2020 DMP | Science Europe DMP | Horizon Europe DMP

There are many factors that can contribute to the risk of information loss or information leaks. They are often part of the behavior of the people that are involved in the project, but can also be steered by properly planned infrastructure.

✔ a. Explore

7.a.1

Do project members store data or software on computers in the lab or external hard drives connected to those computers?

Horizon 2020 DMP | Science Europe DMP | Horizon Europe DMP

When assessing the risk, take into account who has access to the lab, who has (physical) access to the computer hardware itself. Also consider whether data on those systems is properly backed up

✔ a. No

7.a.2

Do project members carry data with them?

Horizon 2020 DMP | Science Europe DMP | Horizon Europe DMP

Does anyone carry project data on laptops, USB sticks or other external media?

✔ a. No

7.a.3

Do project members store project data in cloud accounts?

Think about services like Dropbox, but also about Google Drive, Apple iCloud accounts, or Microsoft Office 365.

✔ a. No

7.a.4

Do project members send project data or reports per e-mail or other messaging services?

✔ a. No

7.a.5

Do all data centers where project data is stored carry sufficient certifications?

Horizon 2020 DMP Science Europe DMP Horizon Europe DMP

✔ b. Yes

7.a.6

Are all project web services addressed via secure http (https://)?

Horizon 2020 DMP Science Europe DMP Horizon Europe DMP

✔ b. Yes

7.a.7

Have project members been instructed about the risks (generic and specific to the project)?

Horizon 2020 DMP Science Europe DMP Horizon Europe DMP

Project members may need to know about passwords (not sharing accounts, using different passwords for each service, and two factor authentication), about security for data they carry (encryption, backups), data stored in their own labs and in personal cloud accounts, and about the use of open WiFi and HTTPS.

✔ b. Yes

7.a.8

Did you consider the possible impact to the project or organization if information is lost?

Horizon 2020 DMP Science Europe DMP Horizon Europe DMP

✔ b. Yes; the effect is small

7.a.9

Did you consider the possible impact to the project or organization if information leaks?

Horizon 2020 DMP Science Europe DMP Horizon Europe DMP

Your institute may have a generic risk assessment; you should be informed about this.

✔ b. Yes; the effect is small

7.a.10

Did you consider the possible impact to the project or organization if information is vandalized?

Horizon 2020 DMP Science Europe DMP Horizon Europe DMP

✔ c. Yes; the risk is acceptably low

7.a.11

Are personal data sufficiently protected?

Horizon 2020 DMP Science Europe DMP Horizon Europe DMP

External Links: *RDMkit on Sensitive Data*, *RDMkit on Human Data*

✔ d. Yes, all data will be anonymized as early as possible

Please note that GDPR law in Europe specifies that data is only anonymous as long as nobody in the world has enough information to re-identify the subject.

Furthermore, GDPR requires that you regularly verify that it is still anonymous.

8

Do you have a contingency plan?

What will you do if the compute facility is down?

✔ b. We have an alternative

V. Interpreting data

The interpretation of the data consists of the last steps of processing (often with manual interventions), visualisation, and data integration. In this chapter many questions about data interoperability will come up.

Summary

Answered (current phase)	7 / 8	
Answered	7 / 9	

Metric	Score
Interoperability	0.00

Questions

1

List the data formats you will be using for interpretation and describe their structure

Give each type of data a name that you recognise.

If you have data in many different structures, integrating the data may be more challenging.

External Links: *RDMkit on Machine Actionability*, *RDMkit on Data Processing*

✗ *This question has not been answered yet!*

2

Will you be doing integration or linking of different data types?

If you are getting different types of data from different sources and want to use them together it is likely that you will need to match items and glue everything together. This can be done with traditional table database technology, but it is also possible to use Linked Data and RDF.

✓ a. No

3
Will you be using common ontologies?

✔ a. No

4
Will there be potential issues with statistical normalization?

✔ a. No

5
Will you be integrating different data sources to get more samples or more data points?

✔ a. No

6
Will you be integrating different data sources in order to get more information for each sample or data point?

✔ a. No

7
Do you have all tools to couple the necessary data types?

✔ b. Yes

8

Will you be using a federated analysis approach?

In some cases it is not practical to bring all data together:

- It may be legally hard to collect the data in one place for analysis
- It may be technically hard to transport data to a single place for analysis

In such cases, a Federated analysis approach may be applicable. Examples of such techniques are DataShield and the Personal Health Train. Secure multi-party computation may be useful too to prevent information leaking between parties.

✗ *This question has not been answered yet!*

9

Will you be doing (automated) knowledge discovery?

Data Stewardship for Open Science: *bzu*

✓ a. No

VI. Preserving data

In this chapter, issues regarding data publication and long term archiving are addressed.

Summary

Answered (current phase)	22 / 22	
Answered	24 / 27	

Metric	Score
Findability	1.00
Accessibility	0.62
Reusability	1.00
Good DMP Practice	0.67

Questions

1

Specify a list of data sets you will be producing

Horizon 2020 DMP maDMP Science Europe DMP Horizon Europe DMP

Add all the data sets you will be producing. Give each a short name, sufficient for yourself to know what data it is about. It is useful to think about a data set as some collection of data that will be ending up in the same place.

External Links: RDMkit on Collecting Data, RDMkit on Data Preserving

✕ *This question has not been answered yet!*

2

Will you be archiving data (using so-called 'cold storage') for long term preservation already during your project?

Horizon 2020 DMP Horizon Europe DMP

Much of the raw data you have will need to be archived for your own later use somewhere. This is often done off-line on tape, not on the disks of the compute facility. Please note that this does not refer to the data publication.

Data Stewardship for Open Science: *kjp*

✔ b. Yes

2.b.1

Is the archived data changing over time, needing re-archival?

Data Stewardship for Open Science: *tgk*

✔ a. No

2.b.2

Will the archive be stored on disk or on tape?

✔ c. Other

2.b.3

Will the archive be stored in a remote location, protecting the data against disasters?

Horizon 2020 DMP | Horizon Europe DMP

✔ a. No

2.b.4

Will the archive need to be protected against loss or theft?

Horizon 2020 DMP | Horizon Europe DMP

✔ a. No

2.b.5

Will your project require the archives to be available on-line?

Data Stewardship for Open Science: *ybd*

✔ b. Yes

2.b.5.b.1

Will data integrity be guaranteed?

If the 'master copy' of the data is available on line, it should probably be protected against being tampered with.

✔ b. Yes

2.b.5.b.2

Is there an interface and a defined process for people to request access to the data?

✔ b. Yes

2.b.6

Has it been established who has access to the archive, and how fast?

✔ a. No

2.b.7

Has it been established how long the archived data need to be kept? For each of the different parts of the archive (raw data / results)?

Data Stewardship for Open Science: *kdp*

✔ b. Yes

2.b.8

Will the data still be understandable and reusable after a long time?

Horizon 2020 DMP Horizon Europe DMP

See also all questions about keeping metadata and data formats. Make sure the metadata is kept close to the data in the archive, and that community supported data formats are used for all long term archiving.

Data Stewardship for Open Science: *zmu*

✔ b. Yes

3

Will you be archiving your data in 'cold storage' after the project finishes?

Horizon 2020 DMP Horizon Europe DMP

Will you be storing (in cold storage) copies of your own data for a longer period after the project has ended? Possibly as a continuation of archival as part of data storage strategy during the project? Data archival is distinct from data publishing, an archive is usually strictly limited in who can access the data.

Data Stewardship for Open Science: *fxe*

✔ b. Yes

3.b.1

Who will be paying for the long term storage?

Horizon Europe DMP

✔ c. This is a service budgeted by one or more of the participating institutes

3.b.2

What is the minimum lifetime of the archive?

Horizon Europe DMP

✔ d. Another period

3.b.2.d.1

For how many years will the archive minimally be kept?

Horizon Europe DMP

✔ 20

3.b.3

Can the archival period be extended?

Horizon Europe DMP

✔ b. Yes

3.b.3.b.1

Who will decide whether the archival period is extended?

Horizon Europe DMP

✔ a. One of the principle investigators involved in the project will decide

3.b.3.b.2

What will the decision whether or not to extend the renewal be based on?

Horizon Europe DMP

- [ ] It will be based on the actual use of the archived data
- [x] It will be based on the predicted use of the archived data
- [ ] It will be based on available budget

3.b.4

Will data formats of data in cold storage be upgraded if they become obsolete?

Horizon Europe DMP

✔ b. Yes

3.b.5

Will data be migrated regularly to more modern storage media (e.g. newer tapes)?

Horizon Europe DMP

✔ b. Yes

4

Will any of the repositories you use charge you for their services?

Horizon 2020 DMP | Science Europe DMP | Horizon Europe DMP

✘ *This question has not been answered yet!*

5

Are there any other recurring fees to keep data or documents available?

Are you using any commercially licensed products to keep data, software or documents available, for which a regular fee must be paid?

✔ b. Yes

Make sure this will be kept running by the department or institute. It is best to also have a backup plan, being able to move data and documents to a different place if a service is discontinued. For this, you may need to arrange permission from all project partners beforehand.

6

Did you budget for the time and effort it will take to prepare the data for publication?

Horizon 2020 DMP | Science Europe DMP | Horizon Europe DMP

✔ a. No

7

Will you be making sure that blocks of data deposited in different repositories can be recognized as belonging to the same study?

✔ c. Yes, all data sets will be linked from a single catalog entry

8

Specify a list of software packages you will be publishing

Specify a short name for each software package.

✖ *This question has not been answered yet!*

9

Will reference data be created?

Will any of the data that you will be creating form a reference data set for future research (by others)?

Much of todays data is used in comparison with reference data. You may be comparing your own data with a "standard set" which is maintained as a collection by someone else. Or you could be determining differences to a standard (for example in bioinformatics, a genome is often compared with a reference genome to identify genomic variants). Will you be creating any data that will be reference data for other researchers?

Data Stewardship for Open Science: *rbz*

✔ a. No

VII. Giving access to data

This chapter deals with the information needed by people who will re-use your data, and with the access conditions they will need to follow.

Summary

Answered (current phase)	7 / 7	
Answered	7 / 7	

Metric	Score	
Reusability	1.00	
Openness	1.00	

Questions

1

Will you be working with the philosophy 'as open as possible' for your data?

Horizon 2020 DMP | Science Europe DMP | Horizon Europe DMP

The FAIR principles do not contain any direction towards "Openness". This is done on purpose, because there can be compelling reasons not to make data "Open", such as privacy, other sensitive data, or intellectual property protection.

The true goal of funding agencies is to create the maximum value for society from their investments. They therefore often add "As open as possible, as closed as necessary" to the requirements for funding.

Data Stewardship for Open Science: *jvm*

✔ b. Yes

2

Can all of your data become completely open over time?

Horizon 2020 DMP | maDMP | Science Europe DMP | Horizon Europe DMP

Some data may be subject to a temporary embargo, or need to stay closed for specific reasons.

✔ b. Yes

3

Will you use temporary restrictions on the reuse of the data (embargo)?

Horizon 2020 DMP | Science Europe DMP | Horizon Europe DMP

✔ d. Yes, data without legal restrictions will be released openly after a fixed time period

> **3.d.1**
>
> **What embargo period are you using?**
>
> Horizon 2020 DMP | Science Europe DMP | Horizon Europe DMP
>
> After what period will restrictions on the reuse of data (except ethical and legal restrictions) fall away?
>
> ✔ No embargo once it has been published

4

Will metadata be available openly?

Horizon Europe DMP

Horizon Europe demands that metadata is completely open, e.g. provided under a CC0 license, and that any deviation from this is clarified.

✔ b. Yes

> **4.b.1**
>
> **Will metadata contain instructions how to get access to the data?**
>
> Horizon Europe DMP
>
> ✔ b. Yes

4.b.2

Will the metadata be available in a form that can be harvested and indexed?

Horizon Europe DMP

Repositories often allow search engines and catalogues to index the metadata in an automated way. Will this be the case for your data?

✔ b. Yes, by the repository / repositories
