# Supplementary material for: Spinal mechanisms and feasibility of Dry Needling versus Botulinum Toxin Type A in post-stroke lower limb spasticity: A proof-of-concept randomized clinical trial protocol (STROKE-POC)
Source: PLoS One. 2026 May 20;21(5):e0334571. doi: 10.1371/journal.pone.0334571 (PMC13189288; doi:10.1371/journal.pone.0334571)
Supplement: Attachment — Submitted filename: Response to Reviewers- Plos One.pdf [file pone.0334571.s006.pdf]

Pablo Herrero Gallego
Department of Physiotherapy, Faculty of Health
Sciences, Universidad de Zaragoza,
50009 Zaragoza, Spain
Tel +34-646168248

Plos One Journal

March 18th, 2026

Dear Editor,

We sincerely appreciate your thoughtful comments and constructive feedback, which have been invaluable in enhancing our manuscript. Below, we provide a detailed response to each of your observations and outline the revisions made to the text accordingly.

All modifications have been highlighted in **blue** within the revised manuscript to facilitate review. We are truly grateful for the time and expertise you devoted to evaluating our work, and we hope the revised version meets your expectations. Please do not hesitate to contact us should any further clarification be required.

Sincerely,

The Authors

Journal Requirements

__Decision__: ***"Please ensure that your manuscript meets PLOS ONE's style requirements, including those for file naming."***

Author response: We have carefully revised the manuscript to ensure full compliance with PLOS ONE's requirements, including file naming conventions and formatting specifications.

The manuscript has been adapted according to the journal's Manuscript Body Formatting Guidelines. In addition, the figure and Supporting Information files have been uploaded separately and named in accordance with PLOS ONE's requirements.

__Decision__: ***"We note that the grant information you provided in the 'Funding Information' and 'Financial Disclosure' sections do not match.***

When you resubmit, please ensure that you provide the correct grant numbers for the awards you received for your study in the 'Funding Information' section."

Author response: Thank you for pointing this out. We have carefully reviewed the information provided in both the "Funding Information" and "Financial Disclosure" sections and have now corrected and aligned the grant numbers to ensure full consistency between the submission system and the manuscript.

The grant numbers and funding bodies have been verified and adjusted accordingly in the "Funding Information" section to match exactly those reported in the manuscript.

We confirm that the funding details are now accurate and consistent across all sections.

__Decision__: ***"When completing the data availability statement of the submission form, you indicated that you will make your data available on acceptance. We strongly recommend all authors decide on a data sharing plan before acceptance, as the process can be lengthy and hold up publication timelines. Please note that, though access restrictions are acceptable now, your entire data will need to be made freely accessible if your manuscript is accepted for publication. This policy applies to all data except where public deposition would breach compliance with the protocol approved by your research ethics board. If you are unable to adhere to our open data policy, please kindly revise your statement to explain your reasoning and we will seek the editor's input on an exemption. Please be assured that, once you have provided your new statement, the assessment of your exemption will not hold up the peer review process."***

Author response: Thank you for this important clarification. A data sharing plan has already been defined. As stated in the manuscript, the STROKE-POC project data will be deposited in publicly accessible repositories (European EBRAINS infrastructure and ELIXIR Core Data Resources).

All shared datasets will be fully anonymized in accordance with GDPR and the approvals obtained from the Research Ethics Committees in Spain, Canada, and Belgium. No identifiable patient information will be included. We confirm that, upon acceptance, the relevant datasets

underlying the results will be made freely accessible in compliance with the journal's open data policy, unless restricted by ethical or legal regulations.

The Data Deposition and Curation section of the manuscript reflects this plan. In addition, the Data Availability Statement in the submission form has been revised to clearly specify the repositories and to confirm that the data will be made openly accessible upon publication.

Decision: ***"Please include your full ethics statement in the 'Methods' section of your manuscript file. In your statement, please include the full name of the IRB or ethics committee who approved or waived your study, as well as whether or not you obtained informed written or verbal consent. If consent was waived for your study, please include this information in your statement as well."***

Author response: We thank the Editor for this comment. We have now added a full ethics statement in the Materials and methods section (subsection "Registration and ethics"), specifying the full names of the ethics committees in Spain, Belgium and Canada, their approval numbers, and the fact that written informed consent is obtained from all participants (or from a legally authorized representative when required). The same wording has been entered in the Ethics Statement field of the submission form, as requested.

The following sections of the manuscript have been revised to incorporate these updates:

Line 340-348

Material and Methods

Registration and ethics

"The study is registered in Clinical Trials (NCT06296082) and Clinical Trials Information System (CTIS) under the number 2024-510866-18-00. Ethical approval was obtained in Spain, Canada, and Belgium. In Spain and Belgium, approval was granted by the Commissie Medische Ethiek GZA Ziekenhuizen (Oosterveldlaan 22, 2610 Wilrijk, Belgium; EU CT number: 2024-510866-18-00).

In Canada, ethical approval was obtained from the Research Ethics Committee of Rehabilitation and Physical Disability (RDP) of the CCSMTL, Integrated University Health and Social Services Centre for South-Central Montreal (approval number: MP-50-2023-1784; date: 30 May 2023).

All participants will provide written informed consent prior to enrolment. For individuals unable to provide consent due to cognitive or communication impairments, written informed consent will be obtained from a legally authorized representative in accordance with local regulations. No waiver of informed consent will be granted."

Decision: ***"If the reviewer comments include a recommendation to cite specific previously published works, please review and evaluate these publications to determine whether they are relevant and should be cited. There is no requirement to cite these works unless the editor has indicated otherwise."***

Author response: Thank you for this clarification. We have carefully reviewed the publications suggested by the reviewers and evaluated their relevance to our study. In particular, we considered the article proposed by Reviewer #3 (Whitehead et al., 2016) regarding sample size planning in pilot trials. As we deemed it methodologically pertinent to our proof-of-concept feasibility design, we have incorporated this reference into the revised Sample size calculation section to strengthen the transparency and justification of our approach.

We appreciate the editorial guidance on this matter.

Reviewer #1:

__Decision__: ***"The authors present a protocol for an interesting study of BTX-A in post-stroke lower limb spasticity. To be a contribution, a pre-enrollment protocol must be sufficiently rich in background information, precise in inclusion criteria, and thorough enough in presenting the rationale for choosing outcome measures. I suggest that the authors explicitly use the SPIRIT 2025 guidelines in preparing a revised manuscript, if the Editor requests one."***

Author response: We thank the reviewer for this constructive comment.

The protocol was originally prepared in accordance with the SPIRIT guidelines, and a completed SPIRIT checklist was already included as supplementary material. In the revised manuscript, we have made the adherence to SPIRIT 2025 more explicit in the Methods section to further clarify alignment with current reporting standards.

We appreciate the reviewer's suggestion, which reinforces the importance of transparent and standardized reporting of clinical trial protocols.

The following sections of the manuscript have been revised to incorporate these updates:

Line 109

Design

"This is a prospective multicenter, proof-of-concept feasibility randomized controlled trial in Spain, Canada, and Belgium. It will include two parallel groups, a multiple-baseline design, and an assessor-blinded approach. The trial follows the SPIRIT 2025 guideline and the WHO Trial Registration Data Set (S1 File) [16], as well as Good Clinical Practice guidelines and the Declaration of Helsinki."

__Decision__: ***"Now for a disclaimer: Prior to my retirement, I had considerable experience in using BTX-A for treatment of spasticity and sometimes pain in children and adolescents with cerebral palsy (CP) and, less frequently, with stroke. I had no intentional experience with dry needling. However, I did notice an immediate reduction in tone immediately after injections many times. In retrospect, this was probably a consequence of dry needling, although it was not recognized as such at the time by me or by others."***

Author response: We sincerely thank you for sharing this insightful reflection based on your extensive clinical experience with BTX-A. Your observation regarding the immediate reduction in tone following injection procedures is highly relevant to the conceptual rationale of our study. As you suggest, immediate changes in muscle tone observed after injection may involve mechanisms beyond the pharmacological action of botulinum toxin, which typically requires several days to manifest. Mechanical stimulation related to needle insertion could plausibly contribute to early modulation of muscle tone.

While dry needling and BTX-A injections differ technically, with DN typically involving multiple insertions using solid, non-beveled filiform needles and BTX-A administered via a single beveled injection, both procedures entail mechanical interaction with muscle tissue. The

inclusion of immediate post-intervention assessments in our protocol allows exploration of potential short-term mechanical effects common to both approaches.

Furthermore, the longitudinal comparison between repeated weekly DN sessions and a single BTX-A injection may help characterize differential temporal patterns of change, potentially distinguishing immediate mechanically mediated effects and exploring whether repeated needling sessions produce cumulative mechanical modulation, in contrast to the delayed neurochemical effects of BTX-A.

We greatly appreciate your comment, which strengthens the mechanistic framework and clinical relevance of this trial.

The following sections of the manuscript have been revised to incorporate these updates:

Line 408-418

Discussion

“Although BTX-A exerts its primary effect through chemical denervation at the neuromuscular junction, both BTX-A injections and DN involve mechanical interaction with muscle tissue. Immediate changes in muscle tone observed after injection procedures may partially reflect mechanically mediated modulation. In contrast, DN involves repeated insertions using solid, non-beveled needles, potentially generating greater cumulative mechanical stimulation over time. The longitudinal design of this study may help explore differential temporal patterns between the immediate mechanical stimulation common to both procedures, the repeated mechanically mediated modulation induced by weekly dry needling sessions, and the delayed neurochemical effects of BTX-A. These patterns may manifest differently across muscular and functional outcomes, as well as central neurophysiological effects of changes in the muscle physiology that will be observed through examination of reflex excitability at the spinal level.”

<u>**Decision**</u>: ***“Considering study background first, a thorough review of the literature about dry needlingly would be helpful for the uninformed reader, like I was. Some specific questions that the authors might consider addressing include: What is the most likely mechanism of action of dry needling for tone reduction? How did you arrive at the weekly frequency of dry needling? Was it based on your personal experience? In your experience or the literature, what is the longest dry needling can be continued without muscle damage? A more thorough summary of the literature on BTX in strokes would also be helpful.”***

Author response: Thank you for this valuable suggestion. We agree that additional background information on dry needling and BTX-A would enhance clarity for readers less familiar with the field.

In the revised manuscript, we have expanded the Introduction to provide a more comprehensive overview of the proposed mechanisms of action of dry needling in neurological populations. Specifically, we now describe its potential effects at the neuromuscular endplate region and its possible modulation of spinal excitability. To support this, we have incorporated evidence from a recent meta-analysis in post-stroke populations (Fernández-de-las-Peñas et al.) as well as preliminary neurophysiological findings evaluating rate-dependent depression of the H-reflex in neurological conditions.

We have also clarified the rationale for the selected weekly dry needling frequency, noting that this schedule is consistent with prior clinical studies and established clinical practice in the management of hypertonia. Additionally, we have briefly addressed safety considerations related to repeated applications based on the available literature.

Finally, we have strengthened and structured the summary of the evidence supporting the use of BTX-A in post-stroke spasticity to provide a clearer context on its established efficacy and mechanism of action.

We believe these revisions provide a more balanced and comprehensive scientific framework for the present study.

The following sections of the manuscript have been revised to incorporate these updates:

Line 78-89

Introduction

"Botulinum Toxin type A (BTX-A) infiltration is currently considered the gold standard for managing post-stroke spasticity [6], while the clinical use of Dry Needling (DN) remains limited despite emerging evidence supporting its potential benefits [7]. BTX-A inhibits acetylcholine release at the neuromuscular junction, producing a temporary chemical denervation that reduces muscle overactivity and spasticity [8]. Consistent with this mechanism, BTX-A has demonstrated clinical effectiveness in reducing post-stroke spasticity and improving functional outcomes and gait [6]. Dry Needling (DN) has also demonstrated effectiveness in reducing spasticity in neurological populations, including post-stroke patients, as reported in a recent meta-analysis [7]. DN is proposed to act at the neuromuscular endplate, similar to BTX-A, but via mechanical rather than chemical mechanisms. Experimental histological studies in animal models have shown that although DN induces minor mechanical injury to muscle fibers after repeated needle insertions, the tissue follows the normal sequence of muscle regeneration, which was nearly complete within approximately one week [9]. Beyond these local effects, preliminary neurophysiological studies suggest that DN may also influence spinal excitability [10]. However, no randomized trial has directly compared their effects on impairment (muscular and spinal mechanisms), functional activity levels (gait and mobility), quality of life, and cost-effectiveness."

Line 139-143

Materials and methods

Interventions to be measured

"Regarding the treatment, BTX-A will be injected once by a clinician, whereas DN will be performed once per week for 12 weeks by an experienced physiotherapist in dry needling. This treatment schedule is consistent with previous clinical studies on dry needling for post-stroke spasticity, which have used repeated sessions over several weeks to achieve sustained effects [18]. Moreover, a once-weekly interval allows adequate time for tissue recovery between sessions, in accordance with proposed muscle repair timelines following needling interventions [9]. Mandatory muscles for treatments are the medial gastrocnemius, the lateral gastrocnemius, the soleus, and the tibialis posterior. Optional muscles are the flexor digitorum longus and brevis, the flexor and extensor hallucis longus [19]."

__Decision__: ***"It is possible that the duration of the interval between the stroke and treatment may affect outcomes. In my experience, patients with stroke sometimes developed dystonia as a late complication (although this may be more common in childhood than in your subjects). For subjects enrolled later in the enrollment period (which, for the Editor, is between the stroke and 12 months after the stroke), do you think that the development of dystonia accompanying spasticity may confound your functional outcomes? It certainly did in my patients with both spasticity and dystonia from stroke. How do you plan to handle this potential confounding variable? Separately, for subjects enrolled very early in the enrollment period, do you think that evolution of tone and/or neurological recovery independent of treatment will affect outcomes and if not, why not? Do you have a plan to identify and address this potential confounder? Although each subject will serve as his/her own control, it would be helpful to know about the effects on outcomes of both of these potential confounders to guide future studies."***

Author response: We thank you for this important and clinically insightful observation. We agree that the time since stroke may influence outcomes, both due to spontaneous neurological recovery in earlier phases and the possible emergence of dystonic features in later stages. The current protocol includes patients with post-stroke spasticity defined by Modified Ashworth Scale scores (1–2), following clinical assessment by experienced clinicians. Although dystonia is not formally evaluated as a separate outcome variable in this feasibility study, the clinical assessment process will allow identification of atypical movement patterns that could influence the interpretation of results. In addition, the use of the Tonic Stretch Reflex Threshold (TSRT) provides a more physiologically grounded assessment of stretch reflex excitability, which may help differentiate mechanisms underlying increased tone. Time since stroke (0–12 months) will be systematically recorded and described. The use of three repeated baseline assessments prior to intervention will help characterize pre-treatment stability and partially account for potential spontaneous evolution of tone in early-stage participants.

As this is a proof-of-concept feasibility trial, one of its objectives is to assess the potential impact of such heterogeneity to better inform stratification and eligibility criteria for future definitive studies.

The following sections of the manuscript have been revised to incorporate these updates:

Line 51 -52

Abstract

"Methods and analysis: This is a protocol of a proof-of-concept, feasibility randomized clinical trial including 90 participants from Canada, Belgium, and Spain who experienced a first stroke in the previous 12 months and present plantar flexor spasticity. Time since stroke (0–12 months) will be recorded and explored as a potential modifier of treatment response. Participants will be randomly assigned to receive either one session of BTX-A or 12 weekly sessions of DN. Blinded evaluators will assess outcomes before, during, and after treatment, with a 4-week follow-up. The primary outcome will be spinal mechanisms of spasticity, measured using the Tonic Stretch Reflex Threshold and its velocity sensitivity. Secondary outcomes will assess: a) muscular architecture and echotexture (measured with ultrasound); b) muscle tone/resistance using the Modified Ashworth Scale; c) gait and mobility (instrumented analysis, Timed Up and Go, 10-Meter Walk Test); d) muscle strength with dynamometry; e) quality of life with the EuroQoL questionnaire; and f) cost-effectiveness (analytic model). The findings will provide preliminary data to inform a future definitive trial."

Line 327 -328

Statistical analyses

"In addition, multivariable linear regression models will be used to explore associations between treatment allocation and changes in the primary outcome (TSRT), adjusting for clinically relevant covariates such as age, sex, time since stroke, and baseline spasticity severity, which will be predefined a priori. Study site (country) will also be included as a covariate to account for the multicenter design. Time since stroke may also be explored as a stratification variable in secondary analyses (e.g., early vs. later post-stroke phases within the 0–12 month window). If required by the data structure (e.g., missing observations or imbalance across time points), linear mixed-effects models may be considered to account for within-subject correlation and covariate adjustment."

Line 419 -428

Discussion

"The Modified Ashworth Scale (MAS) does not fully capture the neurophysiological mechanisms underlying increased tone, including distinctions between spasticity and spastic dystonia, which may limit its value for individualized treatment decisions in heterogeneous post-stroke populations. The Tonic Stretch Reflex Threshold (TSRT), by directly quantifying stretch reflex excitability, offers a more physiologically grounded assessment. This may be particularly relevant in a 0–12 month post-stroke population characterized by variable recovery trajectories and mixed motor presentations. Emerging evidence, including case series reporting differential EMG-based responses to dry needling in patients with spasticity and spastic dystonia [42], further supports the need for more objective physiological measurements to better interpret treatment effects and inform future stratification strategies."

Decision: ***"Considering outcome variables, do you think that your quality-of-life measure is going to be more useful for this lower extremity intervention than a more specific ambulation outcome, such steps per day in activities of daily living, assessed using an accelerometer? I think not. Please refute this suggestion if you decide to stick with a QoL outcome. Please also present studies that validate the outcomes measures obtained using the Montreal Spasticity Reflex Threshold System."***

Author response: We appreciate this thoughtful suggestion. You raise an important point regarding the potential value of real-world ambulation metrics such as steps per day measured with accelerometry.

In this protocol, we deliberately included multiple lower-extremity–specific outcome measures, including muscle architecture and echotexture (ultrasound), resistance to passive stretch (MAS), instrumented gait analysis, Timed Up and Go, 10-Meter Walk Test, and dynamometry. These provide detailed and objective assessments of muscular, biomechanical, and functional performance of the lower limb. In particular, the instrumented gait analysis implemented in this study relies on wearable inertial measurement units (IMUs), which include accelerometers and gyroscopes, allowing precise quantification of spatiotemporal and kinematic gait parameters under standardized conditions. For this reason, we opted for more precise biomechanical gait analysis instruments rather than relying solely on step-count metrics.

The inclusion of a quality-of-life measure (EuroQoL-5D) serves a complementary purpose. While ambulation-specific metrics capture performance capacity, health-related quality

of life reflects the patient's global perception of functional status, participation, and well-being. Given that spasticity affects not only gait but also pain, comfort, and daily functioning, we considered it important to include a standardized and widely validated quality-of-life instrument to capture broader patient-centered outcomes and to enable cost-effectiveness analyses.

We acknowledge that step-count measures obtained through accelerometry can provide valuable ecological information about daily activity levels. However, as this study follows a predefined protocol within a funded research project, modifications to the outcome framework were not feasible at this stage. Nevertheless, we agree that incorporating real-world activity metrics such as daily step counts could strengthen future research, and this suggestion will be considered in the design of subsequent trials.

We appreciate this thoughtful suggestion. It is important to clarify that our instrumented gait analysis is based on wearable inertial measurement units (IMUs), which include accelerometers and gyroscopes. Therefore, objective kinematic and spatiotemporal gait parameters are already captured in a comprehensive and technically robust manner.

While step count in daily life provides useful ecological information, the present protocol focuses on detailed biomechanical gait characterization under standardized conditions, allowing precise quantification of lower-limb performance changes. For this reason, we opted for more precise and mechanistically informative instruments for gait analysis rather than relying solely on step-count metrics obtained from daily activity monitoring.

The inclusion of a health-related quality-of-life measure (EuroQoL-5D) serves a complementary purpose. Beyond gait capacity, spasticity affects pain, comfort, participation, and perceived mobility. Importantly, EQ-5D enables calculation of quality-adjusted life years (QALYs), which are required for cost-effectiveness analyses. Previous economic evaluations in neuromusculoskeletal interventions, including dry needling, have used this instrument for that purpose.

Regarding the Montreal Spasticity Reflex Threshold (MSRT) system, we have now explicitly added references supporting its reliability and construct validity in neurological populations to improve clarity for readers.

The following sections of the manuscript have been revised to incorporate these updates:

Line 234-238

Materials and methods

"Primary outcomes

The MSRT system has been previously used to measure TSRTs in stroke populations. Studies have demonstrated its reliability and clinical applicability in determining the degree of post-stroke spasticity, as well as its construct validity as a motor-control–based measure that also distinguishes spasticity from other forms of hypertonia[27, 28, 29]. These findings support the use of TSRT-derived measures as objective and physiologically grounded outcomes in clinical research."

Line 250

Materials and methods

"They will be measured: 1) Muscle architecture [30] and echotexture [31] with ultrasound imaging, using a portable B-mode system (Butterfly iQ+, Butterfly Network, Inc.) with a 50% gain and 5 cm depth. Participants will be seated with the knee flexed at 90°, and the probe placed at 30% of the distance between the medial tibial condyle and the medial malleolus at a 35–45° angle relative to the horizontal plane. Three transverse and three longitudinal images will be acquired for each participant by a physiotherapist trained in musculoskeletal ultrasound; 2) resistance to passive stretching with MAS [32]. Participants will lie in a supine position, and the examiner will manually move the ankle into dorsiflexion at a consistent speed, maintaining hand contact at the calcaneus and along the plantar surface of the foot to control the movement and perceive the resistance level; 3) gait analysis will be conducted with two precise 3D motion capture systems [33] depending on the country: Xsens system (MVN Awinda, Movella, Hendersen, USA) [34] will be used in Canada and Belgium, and the Move Human Sensors MoCap System [35] will be used in Spain. Participants will walk at a self-selected speed over a 10-meter walkway, with inertial sensors placed on the pelvis, thighs, shanks, and feet to capture kinematic data and spatiotemporal gait parameters; 4) functional mobility and walking speed will be assessed by the 10 Meter Walk Test (10MWT) [36] and the Timed Up & Go (TUG) test [37], both administered by a physiotherapist; 5) muscle strength will be measured with a hand-held dynamometry (MicroFET 2) [38]. Plantarflexion will be tested in seated position with 90° of knee flexion to minimize coactivation, with the dynamometer placed against the metatarsal heads and stabilized against the floor; dorsiflexion will be tested with the examiner holding the device manually in the same position; 6) QoL will be evaluated using the EuroQoL-5D questionnaire [39] and 7) the economic impact of the interventions will be analyzed through a cost-effectiveness assessment by computing the Incremental Cost-Effectiveness Ratio (ICER) expressed in €/QALY. Direct and indirect costs related to treatment, medication, and patient care will be collected throughout the study, including self-reported expenses such as transportation, medical visits, complementary tests, and healthcare resource use. The analysis will compare the clinical effectiveness of BTX-A and DN relative to their associated costs. A non-parametric bootstrapping approach will be used to estimate and represent the uncertainty surrounding the ICER distribution. The final cost-effectiveness evaluation will be performed at the end of the study, although costs will be continuously collected during the intervention period."

Decision: ***"Do you expect to see much clinically significant change from BTX-A on muscle ultrasound at 1month? In CP at least, it takes time to damage the muscle from BTX-A injections. As one BTX-A skeptic memorably said at an international meeting, "Botox injections will eventually swap a muscle condition for a muscle disease"."***

Author response: We sincerely appreciate this insightful and thought-provoking comment. The concern regarding potential long-term structural consequences of BTX-A injections reflects an important ongoing discussion in the field.

Structural muscle changes following BTX-A administration have been documented in previous human studies, particularly at medium- and long-term follow-up intervals (Schroeder et al., 2009; Tok et al., 2011; Mathevon et al., 2015). However, most clinically evident alterations have been described beyond the one-month timeframe.

In the present feasibility study, ultrasound assessment at one month is not intended to assume overt muscle damage, but rather to objectively characterize potential early architectural and echotextural adaptations in a controlled and comparative framework. Clinical scales are often insufficient to detect subtle structural modifications. Quantitative ultrasound biomarkers,

as explored in previous work (e.g., Asadi et al. 2025), may allow identification of early changes not readily apparent on clinical examination.

Therefore, the rationale for early imaging is to explore whether subclinical structural responses can be detected, which may help inform the optimal timing and duration of follow-up imaging in future definitive trials.

The following sections of the manuscript have been revised to incorporate these updates:

Line 430-441

Discussion

"Further evidence is needed to understand the effects of different treatments on muscle architecture and echotexture in post-stroke patients [43] and to clarify their potential architectural and echotextural adaptations. Although major degenerative changes after BTX-A have primarily been described following longer follow-up periods or repeated injections, muscle adaptations detectable by ultrasound have also been reported after single administrations. In post-stroke populations, ultrasound analyses have shown reductions in gastrocnemius thickness and pennation angle following BTX-A injection [13]. Similarly, imaging and histological studies conducted in other populations have reported persistent muscular alterations months after injection [11, 12]. Incorporating ultrasound assessment in the present study allows objective characterization of early muscular responses within a controlled comparative framework. Unlike categorical clinical scales like Heckmatt, which classify muscle involvement into broad severity levels, quantitative ultrasound enables granular assessment of tissue properties [44, 45]. These exploratory data will help determine the optimal timing and relevance of muscle imaging outcomes in future trials with longer follow-up periods."

<u>**Decision**</u>: ***"You state that your multivariate regression models will" include significant variables". What specific covariates do you plan to measure and then adjust for in your regression analyses? Finally, you will likely have done too many outcome analyses for any marginally significant difference to withstand Bonferroni correction. Thoughts about this?"***

Author response: Thank you for this important methodological comment. We have now explicitly specified the covariates to be included in the multivariable regression models: age, sex, time since stroke, baseline spasticity severity, and study site. These variables were selected a priori based on their established clinical relevance and potential influence on treatment response in post-stroke populations.

Regarding multiple comparisons, we acknowledge that the study includes several secondary outcomes. However, this is a proof-of-concept feasibility study primarily designed to evaluate signal detection and inform the design of a future definitive trial. The primary outcome (TSRT) will be analyzed with confirmatory intent, whereas secondary outcomes will be considered exploratory. Rather than relying solely on strict multiplicity correction procedures such as Bonferroni adjustment, interpretation will emphasize effect sizes, confidence intervals, and consistency of direction across related measures. These exploratory findings will guide endpoint prioritization and sample size estimation in future adequately powered trials.

The following sections of the manuscript have been revised to incorporate these updates:

Line 323-327 and 333-334

Materials and methods

"To evaluate treatment effects over time, repeated-measures analyses will be conducted. For normally distributed continuous outcomes, repeated-measures ANOVA will be performed to examine whether changes over time differ between treatment groups. If the assumption of sphericity is violated, Greenhouse–Geisser corrections will be applied. For non-normally distributed outcomes, appropriate non-parametric repeated-measures approaches will be used.

In addition, multivariable linear regression models will be used to explore associations between treatment allocation and changes in the primary outcome (TSRT), adjusting for clinically relevant covariates such as age, sex, time since stroke, and baseline spasticity severity, which will be predefined a priori. Study site (country) will also be included as a covariate to account for the multicenter design. Time since stroke may also be explored as a stratification variable in secondary analyses (e.g., early vs. later post-stroke phases within the 0–12 month window). If required by the data structure (e.g., missing observations or imbalance across time points), linear mixed-effects models may be considered to account for within-subject correlation and covariate adjustment.

Effect sizes (Cohen's d or partial eta squared, as appropriate) and 95% confidence intervals will be reported to support interpretation of exploratory findings and to inform the design of future definitive trials. A two-sided significance level of 5% will be adopted for the primary outcome analyses. Secondary outcomes will be interpreted in an exploratory manner."

Reviewer #2

Decision: ***"BTX-A was administered as a single injection, while DN was given once weekly for 12 weeks. Could this design affect the comparability of the results?"***

Author response: Thank you for this important methodological consideration. The difference in treatment frequency reflects standard clinical practice for each intervention rather than an attempt to equalize the number of sessions. BTX-A is typically administered as a single injection with a sustained pharmacological effect lasting severa weeks up to 3 months (12 weeks), whereas Dry Needling requires repeated applications to achieve cumulative neuromuscular modulation, while the effect of each individual treatment may be resolved by 1 week. Since we want to compare the effects of each treatment over a 12 week period, the single injection of BTX-A is expected to be equivalent to 12 weekly DN treatments.

Our intention was to compare two clinically realistic treatment strategies. This design enhances external validity by reflecting how both interventions are delivered in routine care.

As this is a proof-of-concept feasibility study, the primary aim is to explore mechanisms and estimate effect sizes rather than to establish definitive superiority under strictly dose-matched conditions. Findings from this study will inform the design of future trials, where dose-matching or alternative dosing paradigms could be explored if warranted.

Line 408-418

Discussion

"Although BTX-A exerts its primary effect through chemical denervation at the neuromuscular junction, both BTX-A injections and DN involve mechanical interaction with muscle tissue. Immediate changes in muscle tone observed after injection procedures may partially reflect mechanically mediated modulation. In contrast, DN involves repeated insertions

using solid, non-beveled needles, potentially generating greater cumulative mechanical stimulation over time. The longitudinal design of this study may help explore differential temporal patterns between the immediate mechanical stimulation common to both procedures, the repeated mechanically mediated modulation induced by weekly dry needling sessions, and the delayed neurochemical effects of BTX-A. These patterns may manifest differently across muscular and functional outcomes, as well as central neurophysiological effects of changes in the muscle physiology that will be observed through examination of reflex excitability at the spinal level."

Decision: ***"While assessor blinding is feasible, have the potential effects of the lack of blinding for both patients and operators been considered"***

Author response: Thank you for raising this important methodological point. Due to the nature of the interventions, blinding of participants and treating clinicians is not feasible. We acknowledge that this may introduce potential performance bias, particularly for subjective outcomes.

However, several measures have been implemented to mitigate this risk. Outcome assessments are performed by blinded evaluators, and data analysis, including ultrasound image processing and instrumented gait analysis, is conducted by researchers blinded to treatment allocation.

Importantly, most outcomes in this study are objective and instrument-based, including TSRT measured via the MSRT system, ultrasound imaging, instrumented gait analysis, and dynamometry. Once standardized acquisition protocols are followed, these measurements are not subject to therapist interpretation.

The Modified Ashworth Scale, although examiner-dependent, is included as a secondary outcome and is administered under standardized conditions by trained assessors. Quality of life is assessed using the validated EuroQoL-5D questionnaire, which consists of structured, standardized questions recorded verbatim, thereby minimizing interpretative bias. Cost-effectiveness analyses are based on recorded healthcare utilization and standardized economic modeling procedures.

We have clarified this limitation and the mitigation strategies in the revised manuscript.

The following sections of the manuscript have been revised to incorporate these updates:

Line 279-285

Materials and methods

"Masking

To minimize potential performance and detection bias, outcome assessments are performed by blinded evaluators, and data analysis, including ultrasound image processing and instrumented gait analysis, is conducted by researchers blinded to treatment allocation. Importantly, most primary and secondary outcomes are objective and instrument-based (e.g., TSRT, ultrasound imaging, instrumented gait analysis, dynamometry), reducing susceptibility to expectation-related bias once standardized acquisition protocols are followed. The Modified Ashworth Scale, although examiner-dependent, is administered under standardized conditions and constitutes a secondary outcome"

Reviewer #3

<u>Decision</u>: *"(a) Details on randomization are needed. How are allocation concealment and blinding done? They are not the same thing; the distinction needs to be articulated, keeping in the context."*

Author response: Thank you for this important methodological clarification. We agree that allocation concealment and blinding are distinct procedures and have revised the manuscript to articulate this distinction clearly.

Participants will be randomly assigned to study groups using a computer-generated randomization sequence created in Microsoft Excel. Randomization will be stratified by gender and trial site to ensure balanced allocation across these variables. The randomization sequence was generated by a member of the research team who was not involved in participant recruitment or enrollment. To ensure allocation concealment, the randomization list will be implemented within REDCap (Harris et al., 2009), which automatically assigns participants to the corresponding study group after confirmation of eligibility and entry of baseline data. Study personnel responsible for participant enrolment will not have access to the randomization sequence, thereby preventing prediction of upcoming allocations. Due to the nature of the intervention, blinding of participants and investigators is not feasible; however, allocation will remain concealed during enrolment, and analyses will be conducted using coded group identifiers where applicable.

Harris PA, Taylor R, Thielke R, Payne J, Gonzalez N, Conde JG. Research electronic data capture (REDCap). A metadata-driven methodology and workflow process for providing translational research informatics support. Journal of Biomedical Informatics. 2009;42(2):377–381.

The following sections of the manuscript have been revised to incorporate these updates:

Line 289 and 291-293

Materials and methods

Randomization

Patients will be stratified by spasticity severity, sex, and country and block-randomized (1:1) into 15 pairs per site and assigned to one of the two interventions. The allocation sequence will be generated using a computer-based randomization module within the REDCap platform (Research Electronic Data Capture, Vanderbilt University Medical Center, Nashville, TN, USA).

Allocation concealment will be ensured through centralized electronic assignment within REDCap, such that the allocation sequence is not accessible to recruiting clinicians prior to participant enrollment.

<u>Decision</u>: *"(b) PoC trials are mostly exploratory; hence a formal sample size/power calculation can be excused. Having said that, some kind of formal sample size justification, using targeted effect sizes (say, moderate), is still necessary (see here: https://pmc.ncbi.nlm.nih.gov/articles/PMC4876429/), which the manuscript is lacking. The corresponding sample size/power statements should mention the name of the statistical test, level of significance, and other necessary parameters. It should also be justified using the primary endpoint."*

Author response: We thank the reviewer for this helpful methodological comment. We agree that, even in the context of a proof-of-concept feasibility trial, it is important to provide a more explicit justification of the planned sample size. In the revised manuscript, we have expanded the *Sample size calculation* section to clarify that the target sample of 90 participants (30 per country; 15 per group in each country) follows methodological recommendations for pilot and feasibility studies in clinical research.

We now explicitly state that the sample size is primarily intended to provide reasonably precise estimates of feasibility parameters and variability in the primary outcome (TSRT), rather than to detect definitive efficacy effects. As a plausibility check, we indicate that, assuming a moderate between-group effect size for change in TSRT (Cohen's d ≈ 0.5), a two-sided comparison with α = 0.05 and 80% power would typically require on the order of 60–70 participants in total. Our planned sample of 90 participants therefore represents a conservative choice that allows for potential attrition and the multicenter structure of the trial, while fulfilling the feasibility objectives.

We also reviewed the article suggested by the reviewer (Whitehead et al., 2016) and note that our planned sample size is consistent with its general guidance for pilot trials. We appreciate this comment, which has helped us to strengthen the transparency of our sample size justification.

The following sections of the manuscript have been revised to incorporate these updates:

Line 199-210

Materials and methods

Sample size calculation

"90 participants (30 per country) will be recruited following the Recommendations for Planning Pilot Studies in Clinical and Translational Research [22, 23]. Each group in each country will have 15 patients. This proof-of-concept feasibility randomized controlled trial is not primarily designed to test definitive efficacy hypotheses, but rather to obtain reliable estimates of recruitment, retention, adherence, safety, and preliminary effect sizes to inform the design and sample size of a future definitive trial.

In line with these recommendations, the sample size was chosen to ensure reasonable precision in estimating variability in the primary outcome (Tonic Stretch Reflex Threshold, TSRT) and other key feasibility parameters, rather than to achieve a specific level of statistical power for hypothesis testing. As a plausibility check, assuming a moderate between-group effect size for change in TSRT (Cohen's d ≈ 0.5), a two-sided comparison with an alpha level of 0.05 and 80% power would typically require on the order of 60–70 participants in total. The planned sample of 90 participants therefore provides a conservative margin that allows for potential attrition and the multicenter structure of the trial, while still fulfilling its primary feasibility objectives"

Decision: ***"(c) Statistical/Quantitative Analysis: (c1) Analysis needs to be conducted separately (at each time point), and then combining all points, to assess the differences between the two treatments. The writeup should mention which test will be appropriate for which situation.". "(c2) It's not clear, what the authors meant by ANOVA test. There are 2 groups, where a Student-t, or a Wilcoxon rank-sum will suffice. ANOVA will be helpful for testing > 2 groups, which is likely not the case as per my understanding, at a specific time point. For testing 2 groups (combining multiple time points), a repeated measures ANOVA (or, it's nonparametric counterpart) will be helpful.". "(c3) During the trial, repeated***

measures ANOVA (or a Friedman's test) can be employed. However, additional subject-level covariates will also be collected. Why was a formal linear mixed model, or a GEE not planned, since the total proposed sample size of 90 doesn't look so bad. The ANOVA precludes assessment of group differences without factoring in the covariates, which the mixed models, or GEE can factor in easily.", "(c4) It's appealing to mention the name of the software to be used to conduct the analysis." and "(c5) The sentence "Multivariate regression models will include significant variables..." appears vague. First, the word multivariate is used, when one analyzes multiple outcomes at the same time, adjusted for multiple predictors. Are the authors doing that? Guess not. The correct word can be "multivariable" regression, which implies one response/outcome, regressed upon multiple predictors.
Furthermore, it's not clear what the authors meant by significant variables? Will they be conducting a variable selection? It's not clear. Even before that, how many variables/covariates the trial may generate, prompting them to conduct variable selection? More details needed."

Author response: We thank the reviewer for the detailed and highly constructive statistical comments. We agree that greater clarity and methodological precision were needed.

Primary longitudinal analysis
The primary analysis will be conducted using linear mixed-effects models (LMMs), with fixed effects for treatment group, time, and their interaction (group × time), and a random intercept for participants to account for within-subject correlation. This approach allows simultaneous assessment of between-group differences at each time point and overall longitudinal treatment effects, while appropriately handling repeated measures and potential missing data.

Adjustment for covariates
Prespecified clinically relevant covariates (age, sex, time since stroke, baseline spasticity severity, and study site) will be included as fixed effects in multivariable models. These variables were selected a priori based on theoretical and clinical relevance rather than statistical significance.

Secondary outcomes
Secondary outcomes will be analyzed using analogous mixed-effects modeling strategies. Given the feasibility nature of the trial, these analyses will be considered exploratory, with emphasis placed on effect sizes and confidence intervals rather than strict multiplicity-adjusted hypothesis testing.

Terminology
The term "multivariate" has been corrected to "multivariable," as the analyses involve single outcomes modeled with multiple predictors.

Software
All analyses will be performed using R (R Foundation for Statistical Computing, Vienna, Austria).

The following sections of the manuscript have been revised to incorporate these updates:

Line 307-336

Statistical analyses

"Descriptive statistics will summarize baseline demographic and clinical characteristics by treatment group. Continuous variables will be presented as means and standard deviations or

medians and interquartile ranges, as appropriate, while categorical variables will be described using frequencies and percentages. Normality will be assessed using the Shapiro–Wilk test and visual inspection of histograms.

The primary analysis will focus on changes in the primary outcome (TSRT and its velocity sensitivity). Between-group comparisons at individual time points (baseline, during treatment, post-intervention, and 4-week follow-up) will be performed using independent-samples Student's t-tests for normally distributed continuous variables and Mann–Whitney U tests for non-normally distributed data. Categorical variables will be compared using the Chi-square test or Fisher's exact test, as appropriate.

To evaluate treatment effects over time, repeated-measures analyses will be conducted. For normally distributed continuous outcomes, repeated-measures ANOVA will be performed to examine whether changes over time differ between treatment groups. If the assumption of sphericity is violated, Greenhouse–Geisser corrections will be applied. For non-normally distributed outcomes, appropriate non-parametric repeated-measures approaches will be used.

In addition, multivariable linear regression models will be used to explore associations between treatment allocation and changes in the primary outcome (TSRT), adjusting for clinically relevant covariates such as age, sex, time since stroke, and baseline spasticity severity, which will be predefined a priori. Study site (country) will also be included as a covariate to account for the multicenter design. Time since stroke may also be explored as a stratification variable in secondary analyses (e.g., early vs. later post-stroke phases within the 0–12 month window). If required by the data structure (e.g., missing observations or imbalance across time points), linear mixed-effects models may be considered to account for within-subject correlation and covariate adjustment.

Effect sizes (Cohen's d or partial eta squared, as appropriate) and 95% confidence intervals will be reported to support interpretation of exploratory findings and to inform the design of future definitive trials. A two-sided significance level of 5% will be adopted for the primary outcome analyses. Secondary outcomes will be interpreted in an exploratory manner.

All statistical analyses will be performed using R software (R Foundation for Statistical Computing, Vienna, Austria)."

Decision: ***"(d) Conclusions/Discussion: This section should mention that the expected results from this study are only valid for this pilot sample of patients recruited, and should allude to future trials with much larger sample sizes varying with geographical locations to assess the effectiveness of the competing treatments. The authors need to be commended for at least conducing a 3 country RCT, the scope of which can be extended to multiple countries, depending on resources. At that future stage, innovative analysis are required, alluding to multi-level modeling (say, putting country-level random effects). This can also be part of the Conclusion/Discussion sections."***

Author response: We thank the reviewer for this thoughtful and forward-looking comment. We fully agree that, as a proof-of-concept feasibility trial, the findings will be limited to the recruited pilot sample and should not be interpreted as definitive evidence of comparative effectiveness.

The Discussion has been revised to explicitly acknowledge this limitation and to clarify that the primary aim of this study is to generate preliminary estimates of variability, feasibility metrics, and effect sizes to inform the design of a future adequately powered randomized controlled trial.

We have also emphasized the added value of conducting the study across three countries, which provides important insights into cross-country feasibility and implementation. Future larger-scale trials with broader geographic representation will be required to formally assess comparative effectiveness. At that stage, multilevel modeling approaches incorporating

country-level random effects may be appropriate to account for clustering and inter-country variability.

We appreciate the reviewer's perspective, which has helped position this study within a broader international research trajectory.

The following sections of the manuscript have been revised to incorporate these updates:

Line 451-462

Discussion

"As a proof-of-concept feasibility study, the expected findings will be limited to the pilot sample recruited across the three participating countries and should therefore be interpreted cautiously. The primary aim is not to establish definitive comparative effectiveness, but to generate preliminary estimates of variability, feasibility parameters, and effect sizes to inform the design of a future adequately powered trial. Although participant and operator blinding is not feasible, the use of blinded assessors and predominantly objective, instrument-based outcome measures reduces the risk of bias.

The multinational structure of this study represents an important strength, as it enhances external validity and provides insight into cross-country feasibility. Future definitive randomized controlled trials with larger sample sizes and broader geographic representation will be required to formally evaluate the effectiveness of BTX-A and DN. At that stage, advanced statistical approaches, such as multilevel modeling with country-level random effects, may be appropriate to account for clustering and contextual variability across healthcare systems."

Reviewer #4

Decision: ***"This protocol paper presents a multicenter, randomized clinical trial comparing Dry Needling (DN) and Botulinum Toxin Type A (BTX-A) for post-stroke lower limb spasticity. The study aims to elucidate spinal mechanisms, feasibility, safety, and exploratory effects at muscular, functional, quality-of-life, and cost-effectiveness levels. Ninety participants from Canada, Belgium, and Spain will be randomized to receive either a single BTX-A session or twelve weekly DN sessions, with outcomes assessed by blinded evaluators.***
Strengths of the study
The trial addresses a significant gap: no previous studies have directly compared DN and BTX-A across spinal, muscular, functional, and economic domains in post-stroke spasticity. This is highly relevant given the prevalence of spasticity and the limitations of current treatments. The inclusion of cost-effectiveness and quality-of-life outcomes is forward-thinking, reflecting real-world concerns for patients and healthcare systems. The protocol follows SPIRIT and WHO guidelines, with ethical approval in all participating countries and registration in major trial registries. Blinded outcome assessment and stratified randomization enhance internal validity. The use of objective measures (e.g., Tonic Stretch Reflex Threshold, instrumented gait analysis, ultrasound imaging) is a methodological strength.

Weaknesses of the study: The comparison between a single BTX-A session and twelve DN sessions may introduce bias due to unequal treatment exposure. While this reflects clinical practice, it complicates direct efficacy comparisons and may affect cost-effectiveness analyses. The sample size (90 participants) is justified for feasibility but is not powered for efficacy. This limits the ability to draw definitive conclusions about clinical effectiveness, especially for secondary outcomes. Outcome assessors are blinded, participants and clinicians are not, due

to the nature of the interventions. This could introduce performance and detection bias, particularly for subjective outcomes like quality of life. Inclusion criteria (e.g., first stroke, MAS 1-2, independent ambulation) may exclude more severe cases, limiting applicability to the broader stroke population. The protocol references a Standard Operating Procedure (SOP) and Data Management Plan, but these documents are not included. Full transparency would benefit from their publication as supplementary materials."

Author response: We thank the reviewer for this balanced and constructive appraisal of the study's potential limitations. We address each point below.

Unequal treatment exposure (single BTX-A session vs. 12 DN sessions)

We acknowledge that the difference in treatment frequency may introduce asymmetry in exposure. However, this design reflects real-world clinical practice and was intentionally chosen to preserve ecological validity. BTX-A is typically administered as a single injection with a sustained pharmacological effect lasting several weeks, whereas Dry Needling requires repeated applications to achieve cumulative neuromuscular modulation. Importantly, outcome assessments are performed weekly in both groups, allowing us to characterize the temporal trajectory of treatment effects across time. This repeated-measure structure will enable us to examine patterns of response and onset of effect rather than relying solely on endpoint comparisons. We agree that this may complicate direct efficacy comparisons and cost-effectiveness analyses, and this limitation has been acknowledged in the revised Discussion.

Sample size and lack of power for definitive efficacy

We fully agree that the sample size is not powered for definitive efficacy testing. As explicitly stated in the manuscript, this is a proof-of-concept feasibility randomized controlled trial. The primary purpose is to obtain reliable estimates of feasibility parameters, variability, safety, and preliminary effect sizes to inform the design of a future adequately powered trial. We have further clarified this positioning in the Discussion to avoid any overinterpretation of effectiveness findings.

Blinding limitations and potential bias

Due to the nature of the interventions, participant and clinician blinding is not feasible. However, outcome assessment is performed by blinded evaluators, and data analysis (including ultrasound image processing and instrumental gait analysis) is conducted by researchers blinded to treatment allocation.

Most outcomes are objective and instrument-based (TSRT via MSRT system, ultrasound imaging, instrumented gait analysis, dynamometry). These measurements are not subject to therapist interpretation once data acquisition protocols are followed. The Modified Ashworth Scale is indeed examiner-dependent; however, it is a secondary outcome and is administered under standardized conditions.

Quality of life is assessed using the validated EuroQoL-5D questionnaire, which consists of structured, standardized questions recorded verbatim, thereby minimizing interpretative bias. Cost-effectiveness analyses are based on recorded healthcare utilization and standardized economic modeling.

Restrictive inclusion criteria and generalizability

We agree that restricting inclusion to first stroke, MAS 1–2, and independently ambulatory participants limits generalizability to more severe stroke populations. However, these criteria were selected to:

- Reduce clinical heterogeneity in this initial feasibility phase.
- Allow reliable execution of functional mobility assessments (TUG and 10MWT), which require independent ambulation.
- Avoid excessive variability associated with severe spasticity grades in a pilot sample.

We have acknowledged this limitation in the Discussion and indicated that future definitive trials could expand eligibility criteria to include broader stroke populations.

SOP and Data Management Plan transparency

We appreciate this suggestion. The Standard Operating Procedure (SOP) and the Data Management Plan have now been included as supplementary materials to enhance transparency and reproducibility.

We are grateful for the reviewer's detailed evaluation, which has strengthened the clarity, positioning, and methodological transparency of the protocol.

Decision: ***"Future Studies: The study's findings will provide valuable preliminary data for larger, definitive trials. Future research should consider: Powering for efficacy and including broader patient populations; Exploring long-term outcomes and repeated treatment cycles; Investigating patient-centered outcomes and preferences in greater depth; Publishing detailed SOPs and data management plans for reproducibility."***

Author response: We thank the reviewer for these constructive suggestions. We agree that this proof-of-concept feasibility trial is intended to inform larger, adequately powered definitive studies. In the revised Discussion, we now explicitly acknowledge the need for future trials including broader patient populations, longer-term outcomes, repeated treatment cycles, and deeper evaluation of patient-centered outcomes.

We have also included the SOP and Data Management Plan as supplementary materials to enhance transparency and reproducibility.

Sincerely,

Pablo Herrero Gallego
Department of Physiotherapy, Faculty of Health Sciences, Universidad de Zaragoza
50009 Zaragoza, Spain
Tel + 34-646168248
